# Convergent Evolution of Mitochondrial Genes in Deep-Sea Fishes

**DOI:** 10.3389/fgene.2019.00925

**Published:** 2019-10-03

**Authors:** Xuejuan Shen, Zhiqing Pu, Xiao Chen, Robert W. Murphy, Yongyi Shen

**Affiliations:** ^1^College of Veterinary Medicine, South China Agricultural University, Guangzhou, China; ^2^Centre for Biodiversity and Conservation Biology, Royal Ontario Museum, Toronto, ON, Canada; ^3^Key Laboratory of Zoonosis Prevention and Control of Guangdong Province, South China Agricultural University, Guangzhou, China; ^4^Joint Influenza Research Centre (SUMC/HKU), Shantou University Medical College, Shantou, China

**Keywords:** deep-sea adaptation, mtDNA, adaptive evolution, positive selection, convergent evolution

## Abstract

Deep seas have extremely harsh conditions including high hydrostatic pressure, total darkness, cold, and little food and oxygen. The adaptations of fishes to deep-sea environment apparently have occurred independently many times. The genetic basis of adaptation for obtaining their energy remains unknown. Mitochondria play a central role in aerobic respiration. Analyses of the available 2,161 complete mitochondrial genomes of 1,042 fishes, including 115 deep-sea species, detect signals of positive selection in mitochondrial genes in nine branches of deep-sea fishes. Aerobic metabolism yields much more energy *per* unit of source material than anaerobic metabolism. The adaptive evolution of the mtDNA may reflect that aerobic metabolism plays a more important role than anaerobic metabolism in deep-sea fishes, whose energy sources (food) are extremely limited. This strategy maximizes the usage of energy sources. Eleven mitochondrial genes have convergent/parallel amino acid changes between branches of deep-sea fishes. Thus, these amino acid sites may be functionally important in the acquisition of energy, and reflect convergent evolution during their independent invasion of the harsh deep-sea ecological niche.

## Introduction

Oceans constitute the largest habitat on Earth. Most marine organisms live in the shallower, illuminated depths, and only a few live in the vast darkness of the deep seas (Randall and Farrell, 1997). Deep-sea creatures live below the photic zone and experience hundreds of atmospheres of hydrostatic pressure and constant extreme cold (Robison, 2004). Photosynthesis occurs only down to about 100–200 m, and sunlight disappears altogether at 1,000 m or less. At this depth, there is no light for photosynthesis and animals depend on very limited food floating down from the photic zone. Oxygen is also a limited resource in the deep sea (Childress and Seibel, 1998). Thus, deep-sea organisms must survive in extremely harsh conditions.

Fishes are the charismatic megafauna of the deep sea. Evolutionary adaptations to deep-sea life apparently have occurred independently in at least 22 orders of fishes (Randall and Farrell, 1997). Genetic adaptations for vision to the dark environment of the deep sea is studied well (Hunt et al., 2001; Davies et al., 2009), yet other adaptations remain largely unknown. Biomass available as energy at depths exceeding 1,000 m drops to less than 5% of that available in surface waters (Marshall, 1980). Aerobic metabolism yields much more energy per unit of source material than anaerobic metabolism. Thus, aerobic metabolism should play a greater role than anaerobic metabolism to maximize the use of limited energy sources. However, paradoxically, the level oxygen is low in the deep sea (Childress and Seibel, 1998). Furthermore, high hydrostatic pressure affects the functioning of lipid membranes, enzymes, and other macromolecules (Macdonald, 1997; Robison, 2004). Therefore, proteins involved in the aerobic metabolism of deep-sea fishes must function efficiently to obtain energy in the absence of abundant food and oxygen.

Mitochondria play the most prominent role in aerobic metabolism of the cell by producing ATP through the electron transport chain. All 13 of the mitochondrial protein-coding genes are involved in this. Functional constraints on mitochondrial DNA (mtDNA) genes have been suggested to influence the evolution of locomotion (Shen et al., 2009; Shen et al., 2010), climatic adaptation (Sun et al., 2011), high elevation adaptation (low-oxygen and cold climate) (Luo et al., 2008; Gering et al., 2009; Scott et al., 2011; Wang et al., 2011; Gu et al., 2012; Zhou et al., 2014), adaptive evolution in mammals (da Fonseca et al., 2008), and high hydrostatic pressure adaption of deep-sea animals (Siebenaller and Garrett, 2002). Considering the important role played by mtDNA in aerobic respiration, the environment of deep-sea fishes, and the independent occupation of the deep sea by lineages of fishes, herein we test the hypothesis that adaptive evolution of mitochondrial genes plays a role in deep-sea adaptation.

## Materials and Methods

### Source of Data and Primary Treatments

A total of 2,161 complete mitochondrial genomes of 1,042 fish species were downloaded from NCBI. Considering that human mtDNA is widely studied, we used Cambridge reference sequence for human mtDNA (NC_012920) as the reference to standardize our data. All sequences were aligned by MAFFT with option (FFT-NS-2) (Katoh and Toh, 2010). Information of the depth ranges of these fishes was collected from FishBase (http://www.fishbase.org). Deep-sea fishes often have been considered as those maximum living depth below 1,000 m (Angel, 1997; Pradillon and Gaill, 2007). The depth ranges of 115 species of 1,042 bony fishes fell below 1,000 m (**Supplementary Table 1**). For some orders of fishes, such as Ateleopodiformes, Lophiiformes, Myctophiformes, and Notacanthiformes, either few data was available, or all of the fishes in the orders were deep-sea dwelling without shallow water-dwelling sister taxa; herein, these fishes were not considered further. Finally, the remaining 77 deep-sea fishes were used in this study. They were classed into nine groups according their phylogenetic positions (Betancur et al., 2013): 1) Anguilliformes; 2) Beryciformes and Stephanoberyciformes; 3) Ophidiiformes; 4) Osmeriformes and Stomiiformes; 5) Perciformes; 6) Carangimorpha; 7) Scombriformes; 8) Pleuronectiformes; and 9) Scorpaeniformes.

### Phylogenetic Analysis

Phylogenetic analyses were conducted from 13 concatenated protein-coding genes (*ATP6*, *ATP8*, *COX1*, *COX2*, *COX3*, *CytB*, *ND1*, *ND2*, *ND3*, *ND4*, *ND4L*, *ND5*, and *ND6*) for nine groups separately by ML and Bayesian inference (BI) analysis as implemented in Phyml v3.0 (Guindon et al., 2010). The best-fit models for nucleotide substitutions were selected by jModelTest v0.1.1 (Posada, 2008; Posada, 2009) (**Table 1**).

**Table 1:** Best-fit models for nine deep-sea groups used for phylogenetic reconstruction.

	Groups	Best-fit model	Among-site rate variation	
			I	α
1	Anguilliformes	GTR+I+G	0.3317	0.5895
2	Beryciformes and Stephanoberyciformes	GTR+I+G	0.3941	0.8536
3	Ophidiiformes	GTR+I+G	0.2293	0.5730
4	Osmeriformes and Stomiiformes	GTR+I+G	0.3826	0.7774
	Perciformes (*Thunnus maccoyii*)	TrN+I+G	0.7784	0.7134
5	Perciformes (*Anarhichas denticulatus*)	GTR+I+G	0.4948	1.0584
	Perciformes (*Tetrapturus angustirostris*)	TVM+I+G	0.3400	0.3908
6	Carangimorpha	TVM+I+G	0.5949	1.6423
7	Scombriformes	GTR+I+G	0.7067	87.4913
8	Pleuronectiformes	GTR+I+G	0.3135	0.6203
9	Scorpaeniformes	GTR+I+G	0.4530	1.0906

I, proportion of invariable sites; and α, gamma distribution shape parameter.

### Selection Analyses

Alignments and consensus trees were used for subsequent molecular evolutionary analyses. Positive selection analyses were restricted to those branches leading to the deep-sea fishes. We used a gene-level approach based on the ratio (ω) of nonsynonymous (*Ka*) to synonymous (*Ks*) substitutions rates (ω = *Ka/Ks*) to identify potential positive signals of selection. This analysis employed likelihood ratio tests in the CODEML algorithm of the PAML package (Yang, 1997). Topologies based on single mitochondrial protein-coding genes did not stabilize, while phylogenetic trees that based on combined 13 mitochondrial protein-coding genes were robust. Therefore, we used the combined trees (13 mitochondrial protein-coding genes) for nine groups as guide trees for PAML analyses. The following tests were conducted for 13 genes for nine groups respectively: 1) one-ratio model, which assumes an identical ω value for all branches, was used to detect the overall ω of a gene; and 2) branch-site model was used to determine if these genes had undergone positive selection on a foreground branch. In order to detect whether the positive selection signals were significant, LRT statistics were calculated between branch-site model vs. branch-site model with fixed ω_1_ = 1 (null model).

### Analyses of Convergent and Parallel Evolution

The sequence reconstruction of the ancestor was carried out using the CODEML program implemented in PAML (Yang, 1997). Convergent and parallel amino acid substitutions along deep-sea branches were detected. The statistical significance of the convergent/parallel evolution between two branches was tested by using the method of Zhang and Kumar (1997). Intracellular domains, TM domains, and the extracellular domains of 11 mitochondrial genes were delineated by TMHMM (Sonnhammer et al., 1998; Krogh et al., 2001). Convergent/parallel amino acid sites were mapped to the three-dimensional (3D) protein structures of *ND* (PDB:5LDX) (Zhu et al., 2016), *CytB* (PDB:1PPJ) (Huang et al., 2005), and *COX* (PDB:1V54) (Tsukihara et al., 2003) by using VMD v1.9.3 (Humphrey et al., 1996).

## Results

### Positive Selection on Nine of 13 Mitochondrial Genes in Deep-Sea Fishes

**Figure 1:**
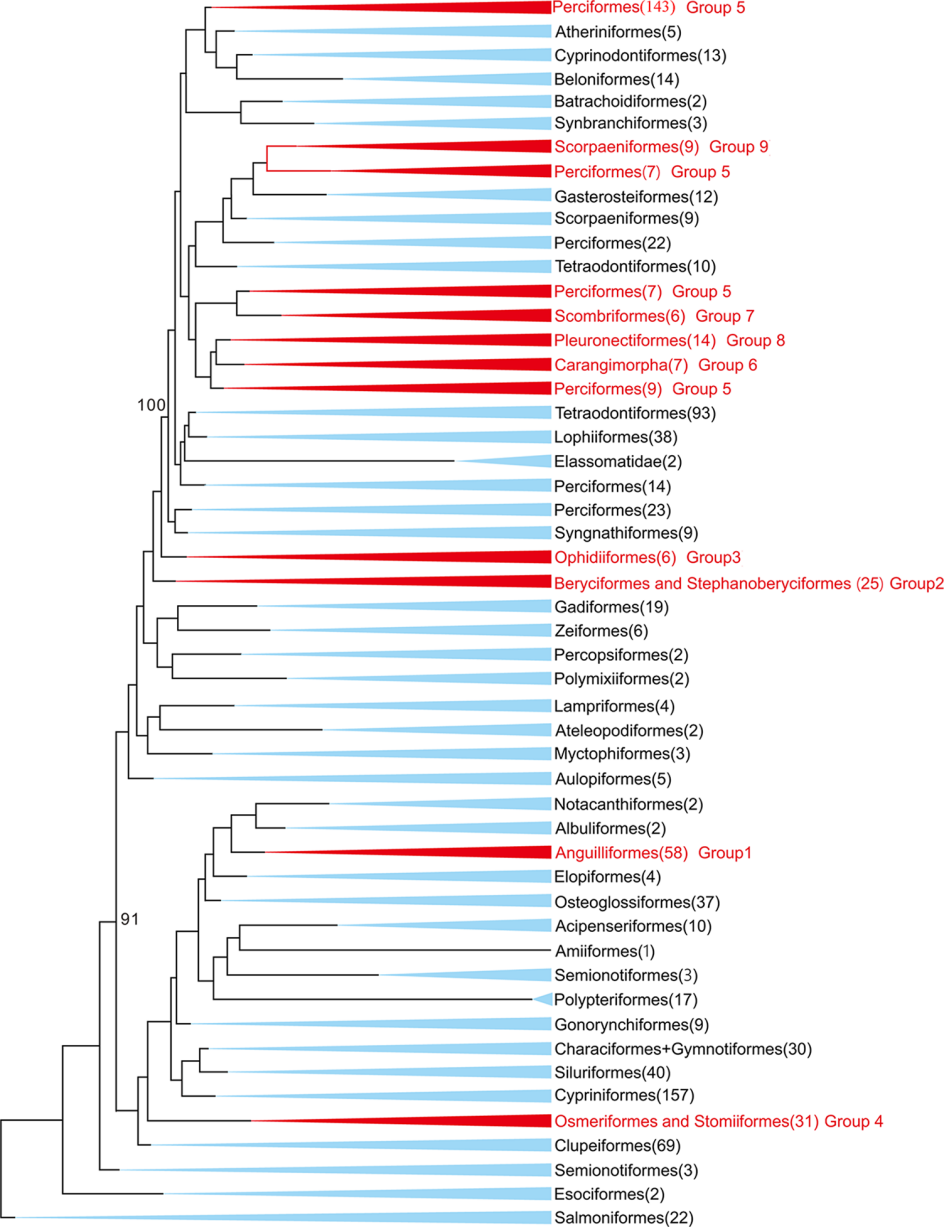
Maximum-likelihood (ML) phylogenetic tree of 1,042 bony fishes. The tree displays 47 orders. The nine groups studied in this study were marked in red. Numbers in the brackets denote available mitochondrial genomes.

**Figure 2:**
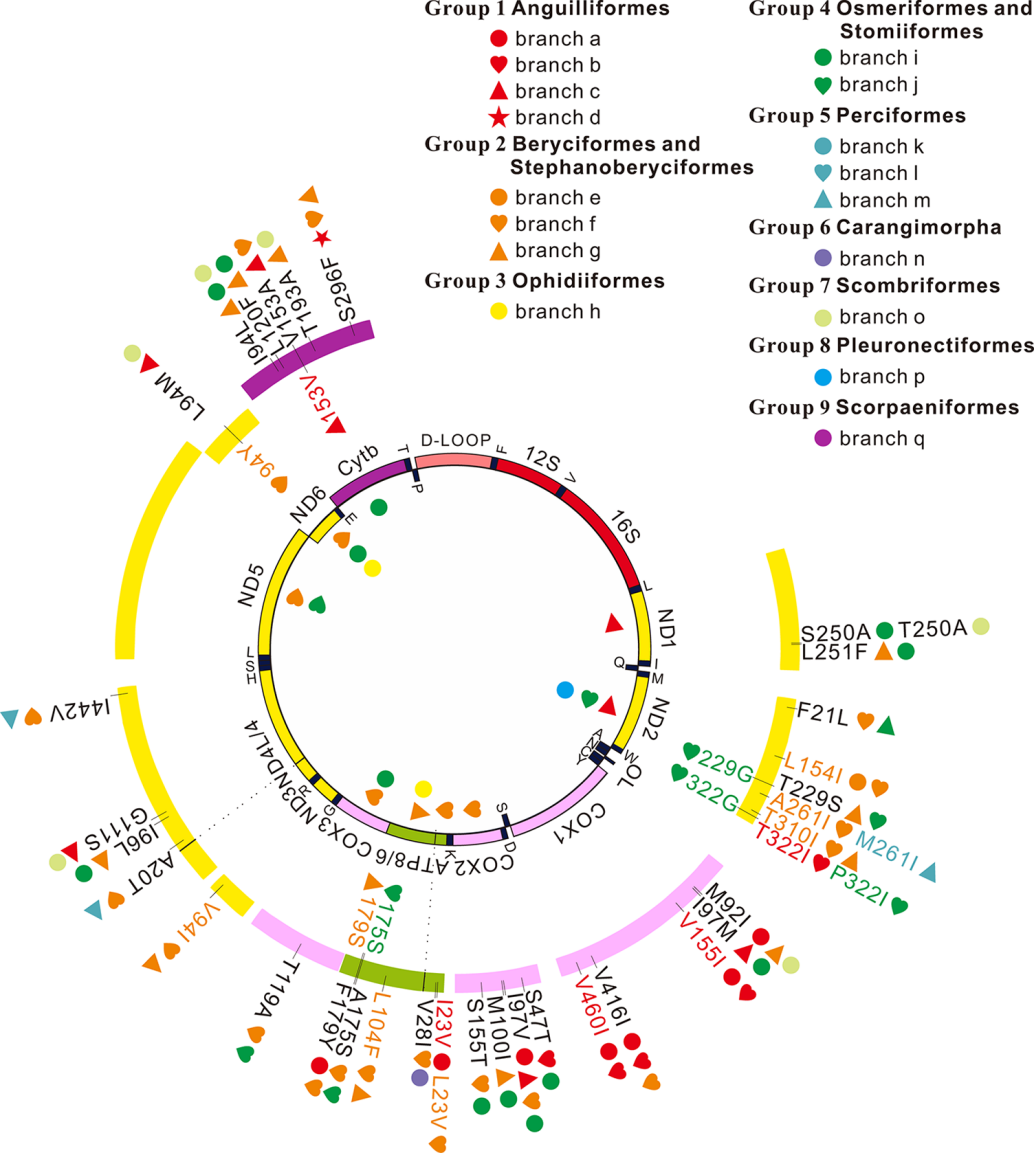
Positive selection and convergent/parallel evolution on 13 mitochondrial protein-coding genes in deep-sea fishes. Symbols inside the interior track denote signals of positive selection and symbols inside the outer track represent those sites that have both signals of positive selection and convergent/parallel evolution. Symbols outside the outer track show convergent/parallel amino acid sites.

In this study, 77 of 115 deep-sea fishes, which divided into nine taxonomic groups, were selected for further analysis (**Figure 1**). The topologies of these nine groups were further constructed and used as the guide tree for positive selection analyses. Analyses using PAML (Yang, 1997) found significant signals of positive selection in the following nine deep-sea branches (**Figure 2**).

Group 1, the true eels (Anguilliformes). Four independent branches of the phylogeny were suggested to have experienced deep-sea adaptation (**Supplementary Figure 1**). Significant signals of positive selection occurred in *ND1* and *ND2* in a cluster of deep-sea eels (**Supplementary Table 2**).

Group 2, Beryciformes and Stephanoberyciformes. At least three independent deep-sea branches were identified (**Supplementary Figure 2**). *ATP6* on branch g (*Anoplogaster cornuta*) and *ATP8*, *COX2*, *COX3*, *ND5*, and *ND6* on branch f (*Diretmoides veriginae* and *Diretmus argenteus*) showed significant signals of positive selection (**Supplementary Table 3**).

Group 3 (Ophidiiformes), which included a cluster of deep-sea fishes (**Supplementary Figure 3**). Selection analyses showed that *ATP6* and *ND6* had significant signals of positive selection on this branch (**Supplementary Table 4**).

Group 4 (Osmeriformes and Stomiiformes) had three branches of deep-sea fishes (**Supplementary Figure 4**). Branch i (*Lipolagus ochotensis*) had significant positive selection signals in *COX3*, *CytB*, and *ND6*, and branch j had significant signals in *ND2* and *ND5* genes (**Supplementary Table 5**).

Group 5 (Perciformes). This group had at least three independent events of deep-sea adaptation (**Supplementary Figure 5**). However, no deep-sea branch had significant signals of positive selection (**Supplementary Table 6**).

Group 6 (Carangimorpha). The group had one deep-sea branch (**Supplementary Figure 6**), but no significant signal of positive selection (**Supplementary Table 7**).

Group 7 (Scombriformes). As Group 6, it had one deep-sea branch (**Supplementary Figure 7**), but no significant signal of positive selection (**Supplementary Table 8**).

Group 8 (Pleuronectiformes) had a deep-sea branch p (*Hippoglossus* and *Reinhardtius*; **Supplementary Figure 8**). *ND2* showed significant signals of positive selection in this branch (**Supplementary Table 9**).

Group 9 (Scorpaeniformes). No any signal of positive selection was detected on its deep-sea branch (**Supplementary Figure 9**, and **Supplementary Table 10**).

### Convergent/Parallel Evolution on 11 of 13 Mitochondrial Genes in Deep-Sea Fishes

Among the 13 mitochondrial protein-genes, 11 genes had convergent/parallel amino acid sites between deep-sea fishes, including 5 convergent amino acid changes and 29 parallel amino acid changes (**Figure 2**; **Table 2**). *ND1* had one statistically significant (*P* < 0.01) parallel amino acid change (L251F) and one convergent change (S/T250A, *P* < 0.001). *ND2* had four significant sites of parallel evolution (L154I, T310I, T229S, and F21L), and two sites of significant convergent evolution (A/M261I and T/P322I). *ND3* had one statistically significant (*P* < 0.05) parallel-evolved site (V94I) on branches f and g. *ND4* had four parallel amino acid substitutions on deep-sea branches: A20T and I442V on branches f and m (*P* < 0.001); G111S on branches c and o (*P* < 0.01); and I96L on branches g and i (*P* < 0.05). For *ND6*, branches c and o shared statistically significant (*P* < 0.01) convergent mutation L/I94M.

**Table 2:** Convergent/parallel sites in 11 of 13 mitochondrial protein-coding genes in deep-sea fishes.

Gene	Parallel evolution			Convergent evolution		
	Amino acid change	Branches	Significance	Amino acid change	Branches	Significance
*ND1*	L251F	g, i	*P* < 0.01**	S/T250A	i, o	*P* < 0.001***
*ND2*	L154I	e, f	*P* < 0.05*	A/M261I	f, m	*P* < 0.01**
	T310I	f, g	*P* < 0.05*	T/P322I	b, j	*P* < 0.001***
	T229S	g, j	*P* < 0.05*			
	F21L	f, i	*P* < 0.05*			
*ND3*	V94I	f, g	*P* < 0.05*			
*ND4*	A20T	f, m	*P* < 0.001***			
	I442V	f, m	*P* < 0.001***			
	G111S	c, o	*P* < 0.01**			
	I96L	g, i	*P* < 0.05*			
*ND6*				L/I94M	c, o	*P* < 0.01**
*COX1*	V155I	a, b	*P* < 0.001***			
	M92I	a, g a, o g, o	*P* < 0.01 **P < 0.01 **P < 0.01**			
	I97M	c, i	*P* < 0.001***			
	V416I	a, b a, f b, f	*P* < 0.001 ***P < 0.05 *P < 0.001***			
	V460I	a, b	*P* < 0.001***			
*COX2*	S47T	b, i	*P* < 0.001***			
	I97V	a, c a, f a, i c, f c, i f, i	*P* < 0.01 **P < 0.05 *P < 0.001 ***P > 0.05 *P* < 0.001 ***P < 0.05*			
	M100I	g, i	*P* < 0.001***			
	S155T	f, i	*P* < 0.05*			
*COX3*	T119A	f, j	*P* < 0.01**			
*ATP6*	L104F	f, g	*P* > 0.05			
	A175S	f, j	*P* > 0.05			
	F179Y	a, f	*P* > 0.05			
*ATP8*	V28I	f, n	*P* < 0.01**	I/L23V	a, f	*P* < 0.01**
*CytB*	I94L	g, i g, o i, o	*P* < 0.001 ***P < 0.001 ***P < 0.001***			
	L120F	g, i	*P* < 0.001***			
	V153A	c, f	*P* < 0.05*			
	T193A	g, o	*P* < 0.001***			
	S296F	d, f d, g f, g	*P* < 0.05 *P < 0.05 *P < 0.05*			

*P < 0.05; **P < 0.01; ***P < 0.001.

*COX1* had the following five parallel amino acid sites: V155I and V460I on branches a and b (*P* < 0.001); I97M on branches c and i (*P* < 0.001); V416I on branches a, b, and f; and M92I on branches a, g, and o. *COX2* had four parallel amino acid changes: I97V was shared by branches a, c, f, and i; S47T on branches b and i (*P* < 0.001); M100I on branches g and i (*P* < 0.001); S155T on branches f and i (*P* < 0.05). *COX3* had significant (*P* < 0.01) parallel substitution T119A on branches f and j.

*ATP6* had three parallel amino acid changes (L104F, A175S, and F179Y), but they were not statistically significant (*P* > 0.05). *ATP8* had statistically significant (*P* < 0.01) parallel site V28I on branches f and n, and a convergent change I/L23V on branches a and f.

*CytB* had five parallel amino acid changes: I94L on branches g, I, and o; S296F on branches d, f, and g; L120F on branches g and i (*P* < 0.001); V153A on branches c and f (*P* < 0.05); and T193A on branches g and o (*P* < 0.001).

**Figure 3:**
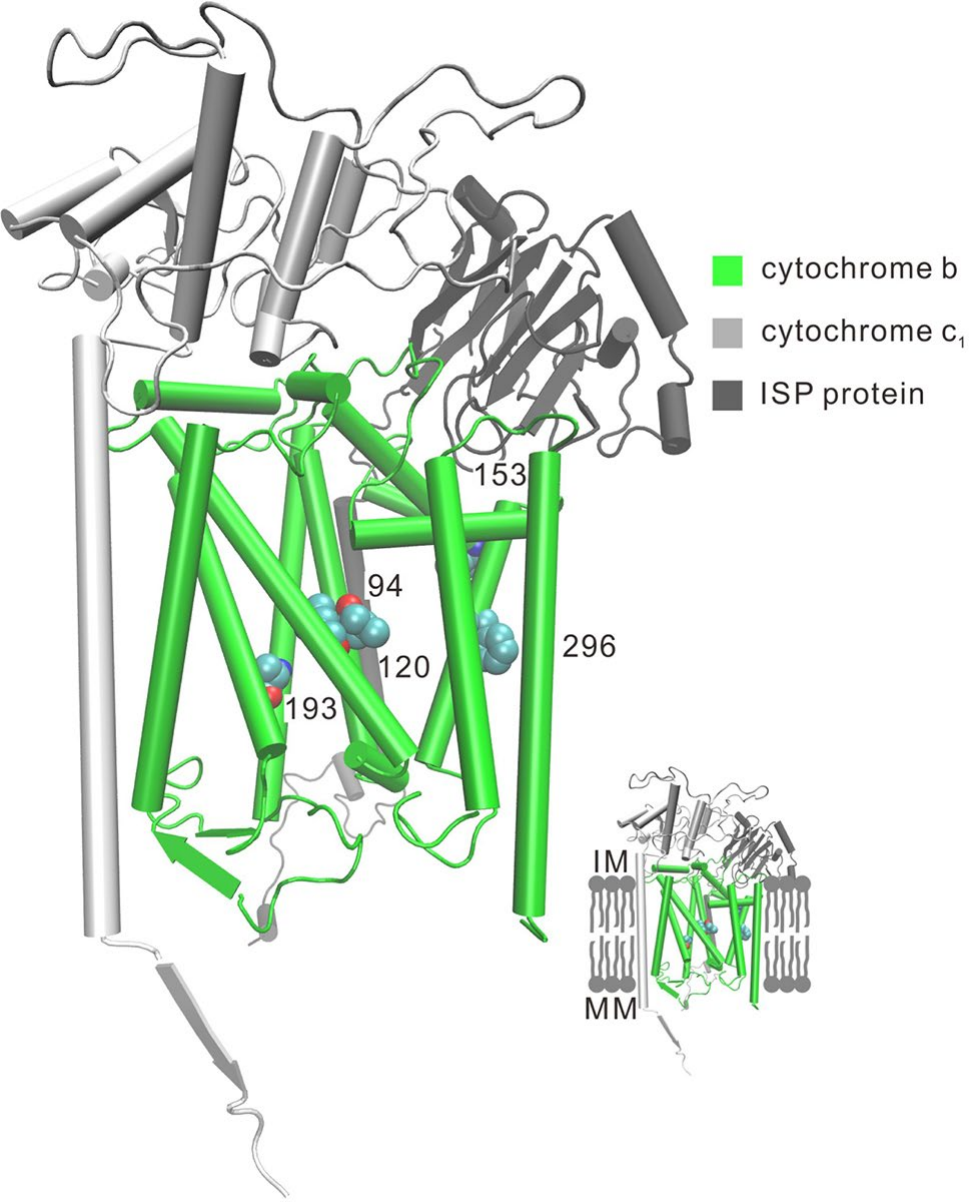
Three-dimensional representation of parallel amino acid sites in deep-sea fishes in cytochrome *b*. Five parallel amino acid sites I94L, L120F, V153A, T193A, and S296F that occur in the TM subunit in *CytB* were mapped to the bovine *CytB* structure (PDB:1PPJ). MM, mitochondria matrix; IM, intermembrane space.

Convergent/parallel amino acid sites in deep-sea fishes were mapped to the available 3D structure of *CytB* (**Figure 3**), *ND* (**Supplementary Figure 10**), and *COX* (**Supplementary Figure 11**) to facilitate interpretations of their positions. Eighteen convergent/parallel amino acid changes located in the transmembrane (TM) domain and the remaining 16 changes were located in other domains. However, all five parallel amino acid changes in *CytB* protein occurred in TM subunits; two of them (T193A and S296F) changed from neutral polarity to nonpolar amino acids, while the other three did not change their polarity.

## Discussion

Due to the limited sources of food, deep-sea fishes appear to have maximized their usage of energy sources. Aerobic metabolism yields much more energy *per* unit of source material than anaerobic metabolism. Our study reveals signals of adaptive selection (positive selection and convergent/parallel evolution) in mitochondrial genes of deep-sea fishes (**Figure 2**). This result corresponds with our hypothesis that aerobic metabolism plays an important role in deep-sea fishes to maximize the usage of limited energy sources.

Positive selection drives the accumulation of advantageous mutations, and thus is associated with the adaptation of new environment and the evolution of new function (Nielsen, 2005; Xiang et al., 2018). During the deep-sea invasions, nine genes (*ND1*, *ND2*, *ND5*, *ND6*, *COX2*, *COX3*, *ATP6*, *ATP8*, and *CytB*) appear to have experienced positive selection. A previous study suggested that *ND4*, *CytB*, and *ATP8* played a role in the origin of flight in bats to fit for the huge change in energy demand (Shen et al., 2010). Further, *ATP6*, *ND2*, and *ND4* genes were suggested to associate with high-elevation adaptation in galliform birds (Zhou et al., 2014). All of the 13 mitochondrial genes play important roles in aerobic metabolism, and this may explain why different studies discover different adaptive genes.

Deep-sea fishes have independently invaded deep-sea habitats several times. Due to the similar environmental pressures, convergent/parallel evolution in key genes likely occurs, when similar morphological or physiological changes occur in multiple lineages (Zhang and Kumar, 1997; Shen et al., 2012). To identify genes and amino acid sites that are important for the multiple deep-sea adaptations, our analyses of convergent/parallel evolution detect 5 convergent amino acid changes and 29 parallel amino acid changes in 11 mitochondrial genes (**Figure 2**, **Table 2**). Some parallel amino acid changes occur on several branches. For example, I97V in *COX2* occurs on four deep-sea branches, and V416I and M92I of *COX1* and I94L and S296F of *CytB* occur on three. Some deep-sea branches have many parallel amino acid changes in one gene. For example, in *COX1*, branch a has four parallel amino acid changes and branch b has three. Previous assays revealed that convergent/parallel amino acid changes were responsible for convergent/parallel functional changes (Yokoyama and Radlwimmer, 1998; Zhang, 2006). The multiple occurrences of convergent/parallel evolution in mitochondrial proteins in deep-sea fishes suggest that mitochondria play important roles in adaptation of fishes to deep sea. This may also reflect that only a few amino acid sites are critical for mitochondrial adaptation to deep seas.

For the complex of electron transport chains, TM and other domains have approximately the same numbers of convergent/parallel amino acid changes (18 vs. 16, respectively). For the former changes, two of the five parallel amino acid changes (T193A and S296F) in *CytB* occur in TM subunits of the protein and they change polarity of the amino acids from polar to nonpolar (**Figure 3**). *CytB* catalyzes reversible electron transfer from ubiquinol to cytochrome c coupled to proton translocation (Trumpower, 1990). Nonpolar amino acids are mainly hydrophobic. Thus, the parallel hydrophobic changes in the TM subunits likely make the protein more stable. For deep-sea animals, the high hydrostatic pressure orders phospholipid bilayers, causing the fatty acyl chains to pack together more tightly. This property can have extremely detrimental effects on the functioning of the lipid membrane of cells, influencing membrane enzymatic processes (Siebenaller and Garrett, 2002). Proteins of the oxidative phosphorylation (OXPHOS) system are all membrane proteins. The parallel hydrophobic changes in the TM subunits are likely a better fit of the protein to the more viscous membrane fluidity due to high hydrostatic pressure.

Except for *ND4L* and *ND5*, the other 11 mitochondrial genes exhibit either positive selection or convergent/parallel signals. Six sites appear to have undergone both positive selection and convergent/parallel evolution (**Figure 2**). However, further investigation is necessary to determine the roles these genes and sites play in deep-sea adaptation.

Some deep-sea branches do not exhibit signals of positive selection or convergent/parallel evolution. However, adaptive evolution has other mechanisms such as increased densities of mitochondria (Mahalingam et al., 2017), larger mitochondria, changes of gene expression levels, and phenotypic plasticity.

Deep-sea creatures are among the most amazing forms of life. They survive in extremely harsh conditions, such as hundreds of atmospheres of hydrostatic pressure, small amounts of oxygen, very little food, no sunlight, and constant extreme cold. The genetic basis of their adaptation to the deep-sea ecological niche remains a mystery. Our study reveals multiple signals of adaptive evolution (positive selection and convergent/parallel evolution) on mitochondrial genes in deep-sea fishes. Positive selection on mitochondrial genes may help deep-sea fishes to maximize the usage of limited energy sources, and thus drive energetic survival in hash deep-sea environment. In addition, multiple convergent/parallel changes in mitochondrial genes may reflect that these amino acid sites are functional importance for the mitochondria to acquire energy, and reflect convergent evolution during their independently invaded harsh deep-sea habitats.

## Data Availability Statement

Publicly available datasets were analyzed in this study. These data can be found here: All the accession numbers were listed in **Supplementary Table 1**.

## Author Contributions

YS conceived and designed the research. XS, ZP, and XC collected and analyzed the data. YS and XS wrote the manuscript. RM revised the manuscript.

## Funding

This work was supported by National Natural Science Foundation of China (31822056 and 41666008), Guangdong Natural Science Funds for Distinguished Young Scholar (2014A030306046), start-up funding from South China Agricultural University for YS, and a Visiting Professorship for Senior International Scientists from the Chinese Academy of Sciences to RM.

## Conflict of Interest

The authors declare that the research was conducted in the absence of any commercial or financial relationships that could be construed as a potential conflict of interest.

## Supplementary Material

Figure S1 Phylogeny of Anguilliformes used for the detection of positive selection. Deep-sea species were marked in red.

Figure S2 Phylogeny of Beryciformes and Stephanoberyciformes used for detection of positive selection. Deep-sea species were marked in red.

Figure S3 Phylogeny of Ophidiiformes used for detection of positive selection. Deep-sea species were marked in red.

Figure S4 Phylogeny of Osmeriformes and Stomiiformes used for detection of positive selection. Deep-sea species were marked in red.

Figure S5 Phylogeny of Perciformes used for detection of positive selection. Deep-sea species were marked in red.

Figure S6 Phylogeny of Carangimorpha used for detection of positive selection. Deep-sea species were marked in red.

Figure S7 Phylogeny of Scombriformes used for detection of positive selection. Deep-sea species were marked in red.

Figure S8 Phylogeny of Pleuronectiformes used for detection of positive selection. Deep-sea species were marked in red.

Figure S9 Phylogeny of Scorpaeniformes used for detection of positive selection. Deep-sea species were marked in red.

Figure S10 Three-dimensional representation of convergent/parallel amino acid sites in deep-sea fishes in the NADH-ubiquinone oxidoreductase chain 1, 2, 3, 4, 4L, 5 and 6. Convergent/parallel amino acid sites within NADH subunits are mapped to the bovine ND structure [PDB:5LDX]. Parallel amino acid sites S250A and L251F occur in ND1; Parallel amino acid sites F21L, L154I, T229S and T310I and convergent amino acid sites A/M261I and T/P322I occur in ND2. ND3 has one parallel amino acid site V94I. Parallel amino acid sites A20T, I96L, G111S and I442V occur in ND4 and parallel amino acid site L94M is in ND6. Red indicates mutations located in intracellular domain, TM subunit sites are in black, and blue shows extracellular domain sites. MM: mitochondrial matrix; IM: intermembrane space.

Figure S11 Three-dimensional representation of parallel amino acid sites in deep-sea fishes in cytochrome c oxidase subunits I, II and III. Ten parallel amino acid sites map to the bovine COX structure [PDB:1V54]. Sites M92I, I97M, V155I, V416I and V460I occur in COX1, sites S47T, I97V, M100I and S155T are in COX2, and site T119A is in COX3. Mutation sites located in the intracellular domain are shown in red while TM subunit sites are shown in black. MM: mitochondria matrix; IM: intermembrane space.

Table S1 The depth-range of 115 species of 1,042 bony fishes.

Table S2 Selective pressure analyses for the 13 genes in Anguilliformes.

Table S3 Selective pressure analyses for the 13 genes in Beryciformes and Stephanoberyciformes.

Table S4 Selective pressure analyses for the 13 genes in Ophidiiformes.

Table S5 Selective pressure analyses for the 13 genes in Osmeriformes and Stomiiformes.

Table S6 Selective pressure analyses for the 13 genes in Perciformes.

Table S7 Selective pressure analyses for the 13 genes in Carangimorpha.

Table S8 Selective pressure analyses for the 13 genes in Scombriformes.

Table S9 Selective pressure analyses for the 13 genes in Pleuronectiformes.

Table S10 Selective pressure analyses for the 13 genes in Scorpaeniformes.
