# Supplementary material for: Convergent Evolution of Mitochondrial Genes in Deep-Sea Fishes
Source: Front Genet. 2019 Oct 3;10:925. doi: 10.3389/fgene.2019.00925 (PMC6785628; doi:10.3389/fgene.2019.00925)

Supplementary Figure 1

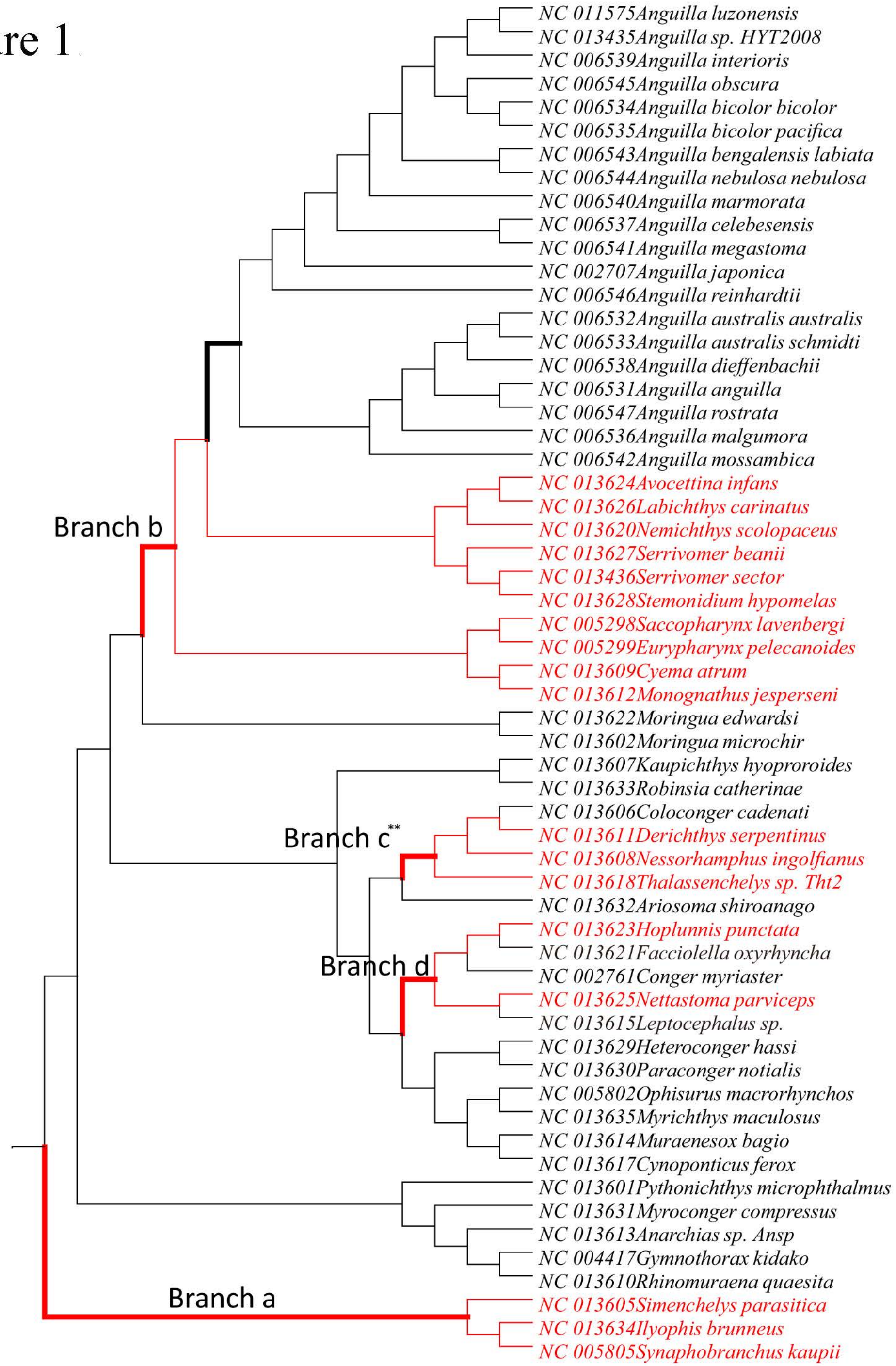

Supplementary Figure 2

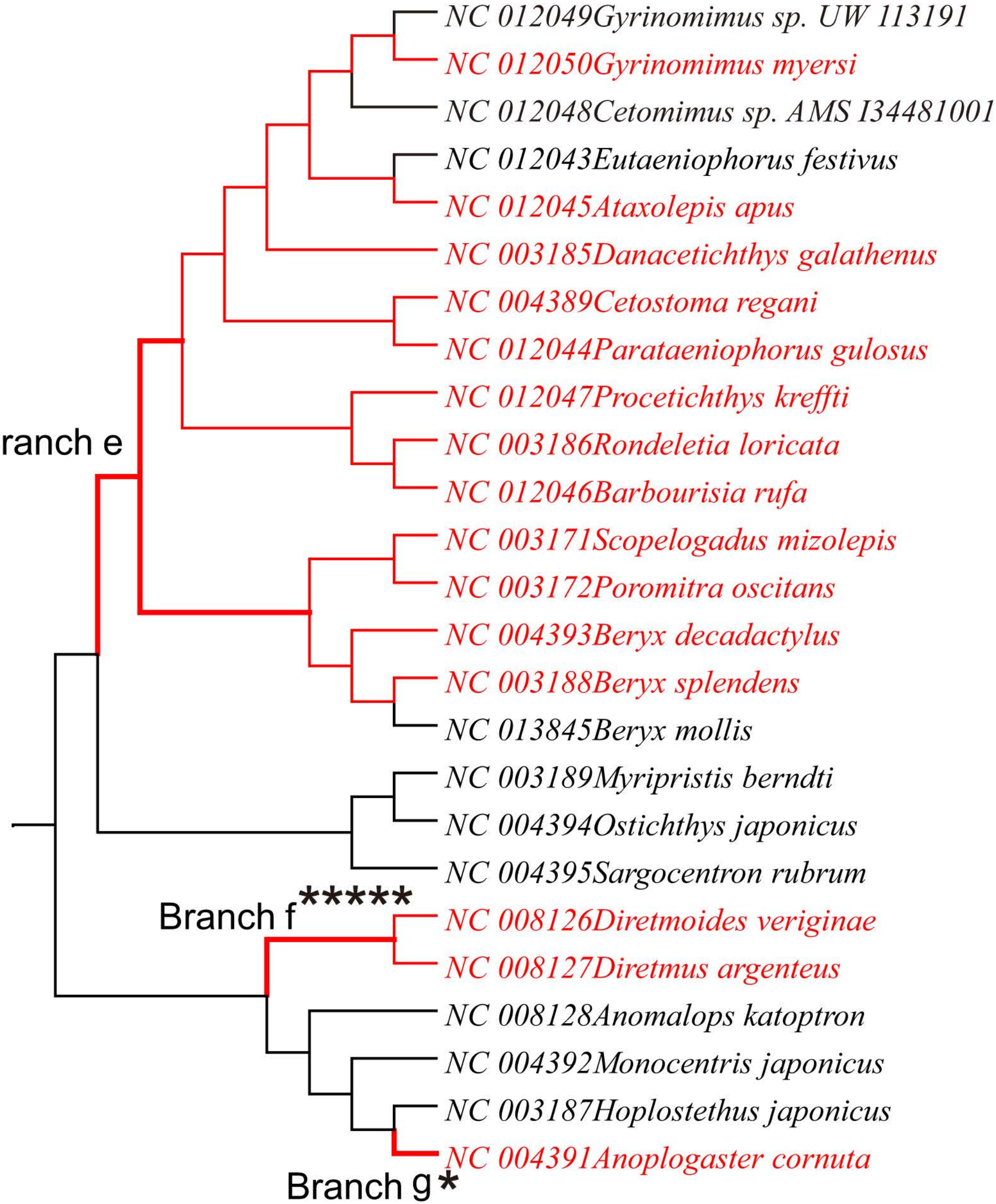

Supplementary Figure 3

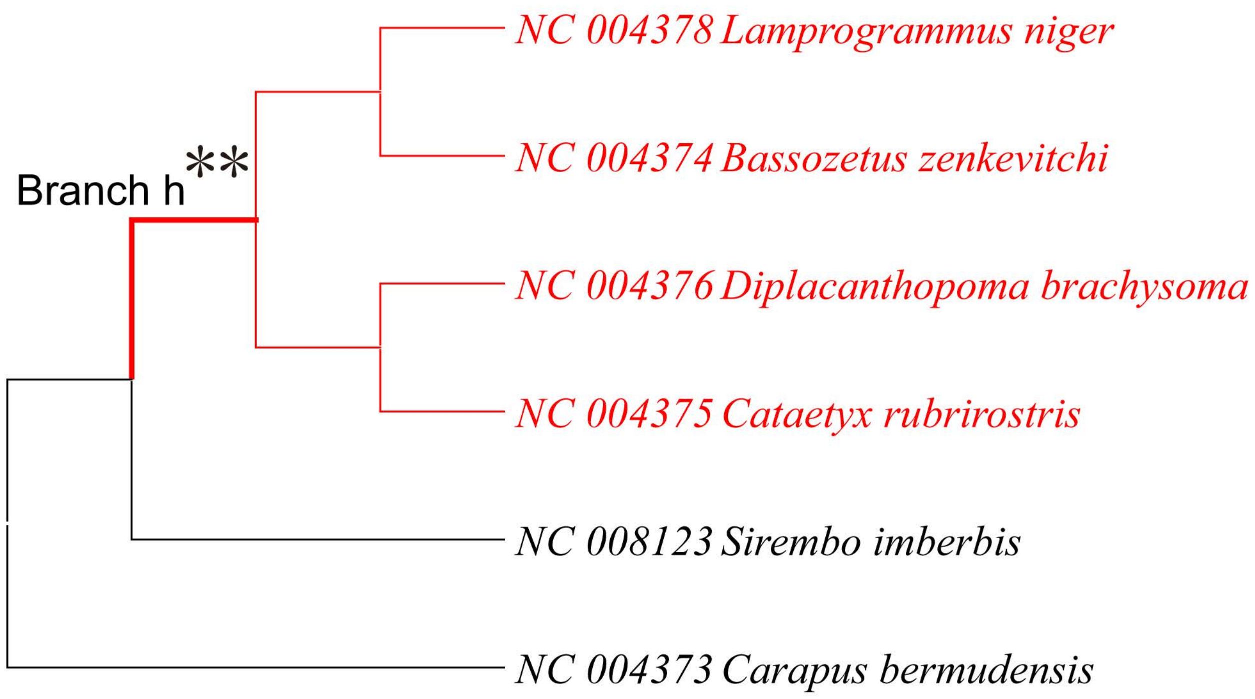

Supplementary Figure 4

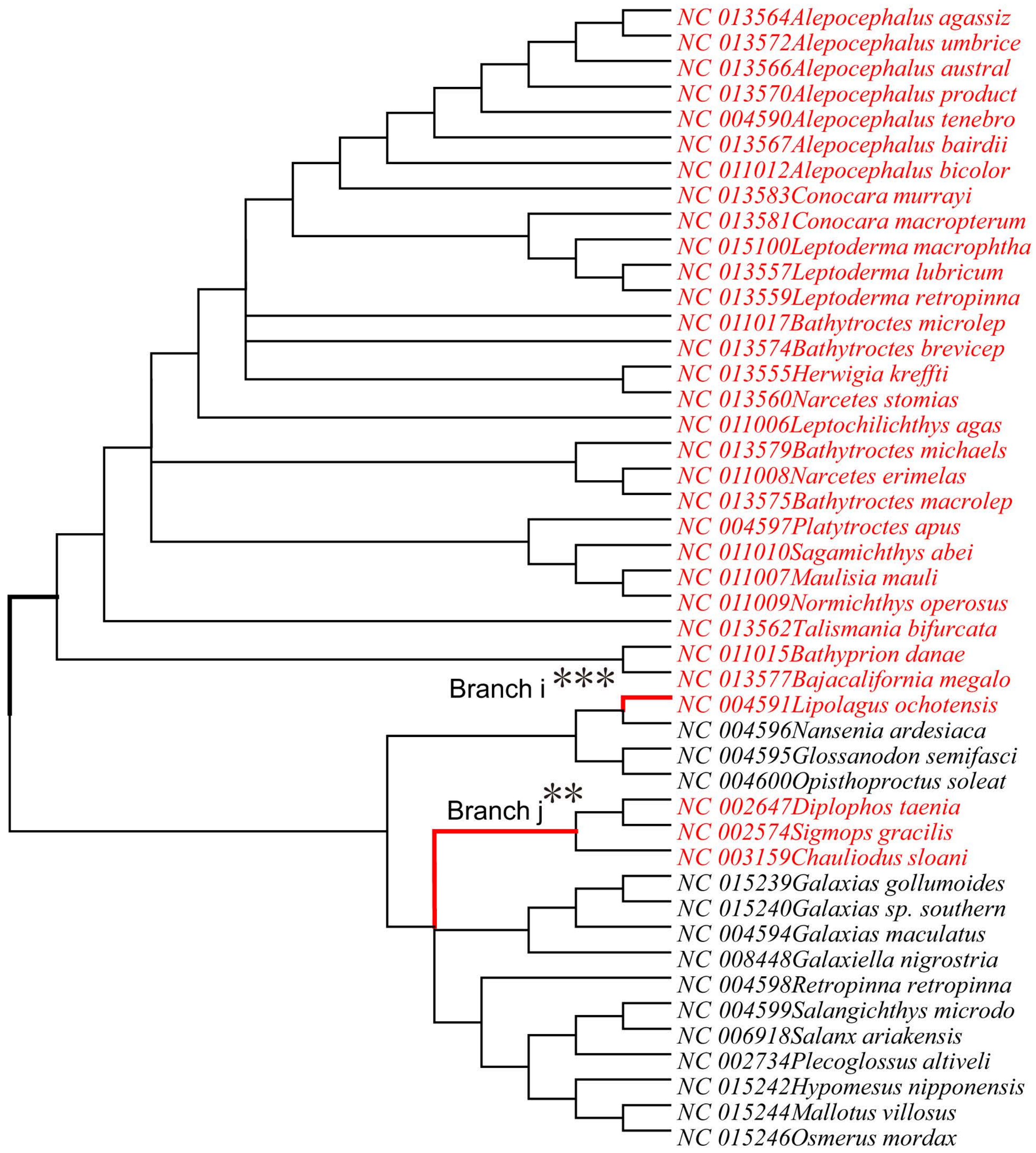

Supplementary Figure 5.

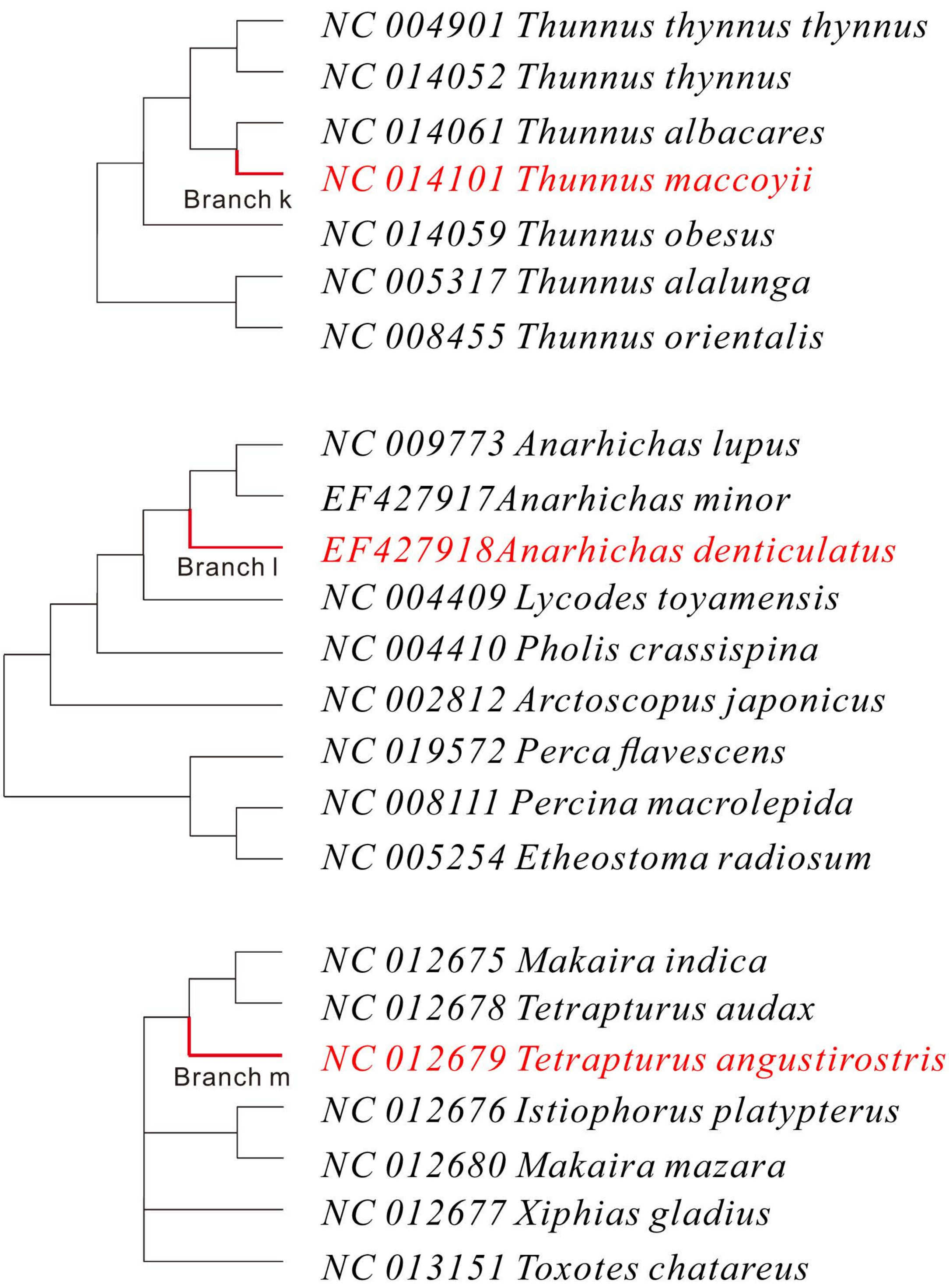

Supplementary Figure 6.

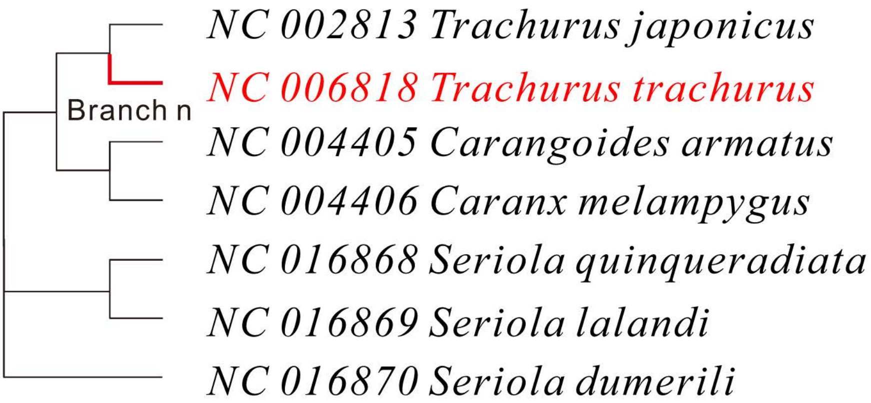

Supplementary Figure 7

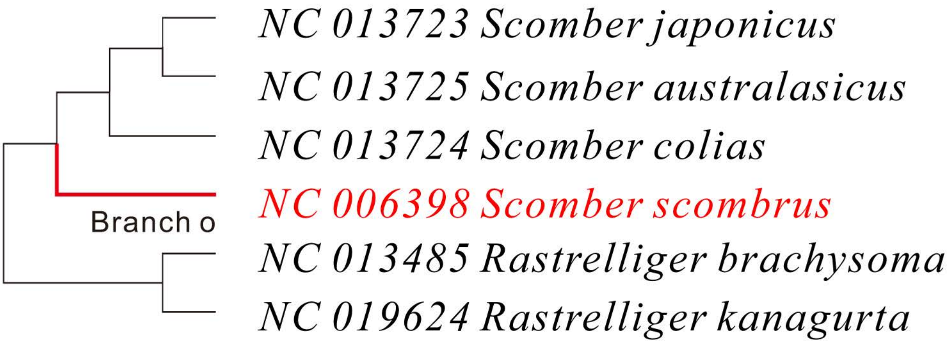

Supplementary Figure 8

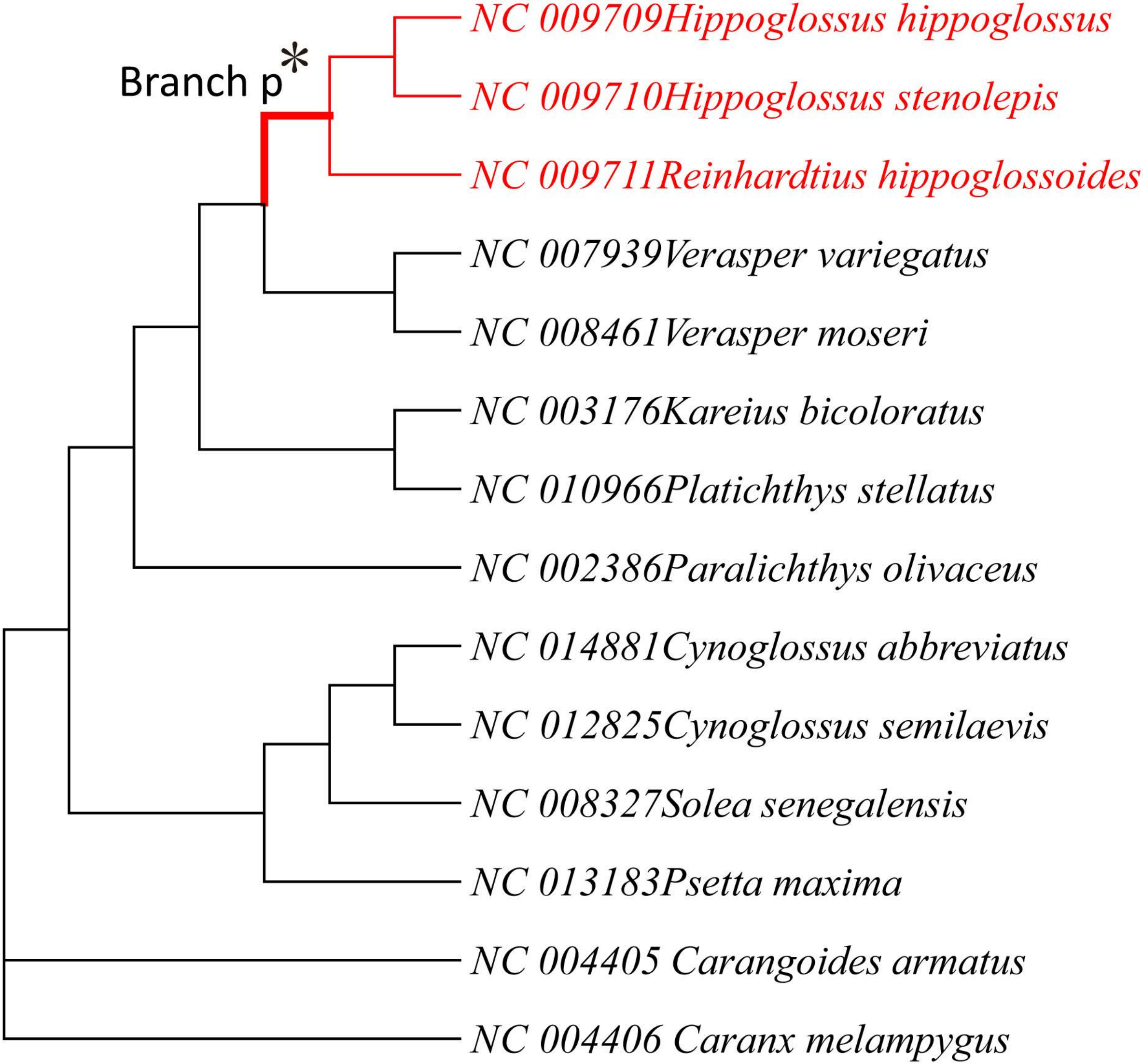

Supplementary Figure 9

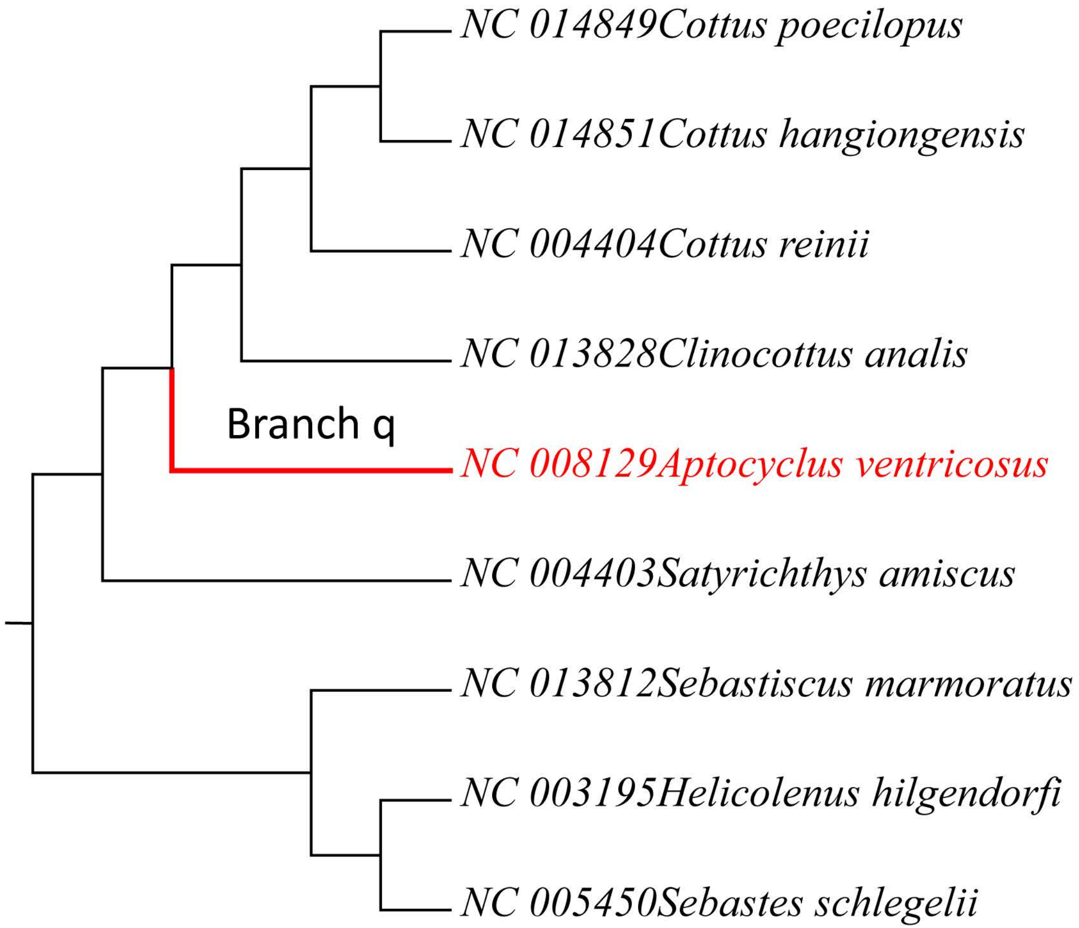

Supplementary Figure 10

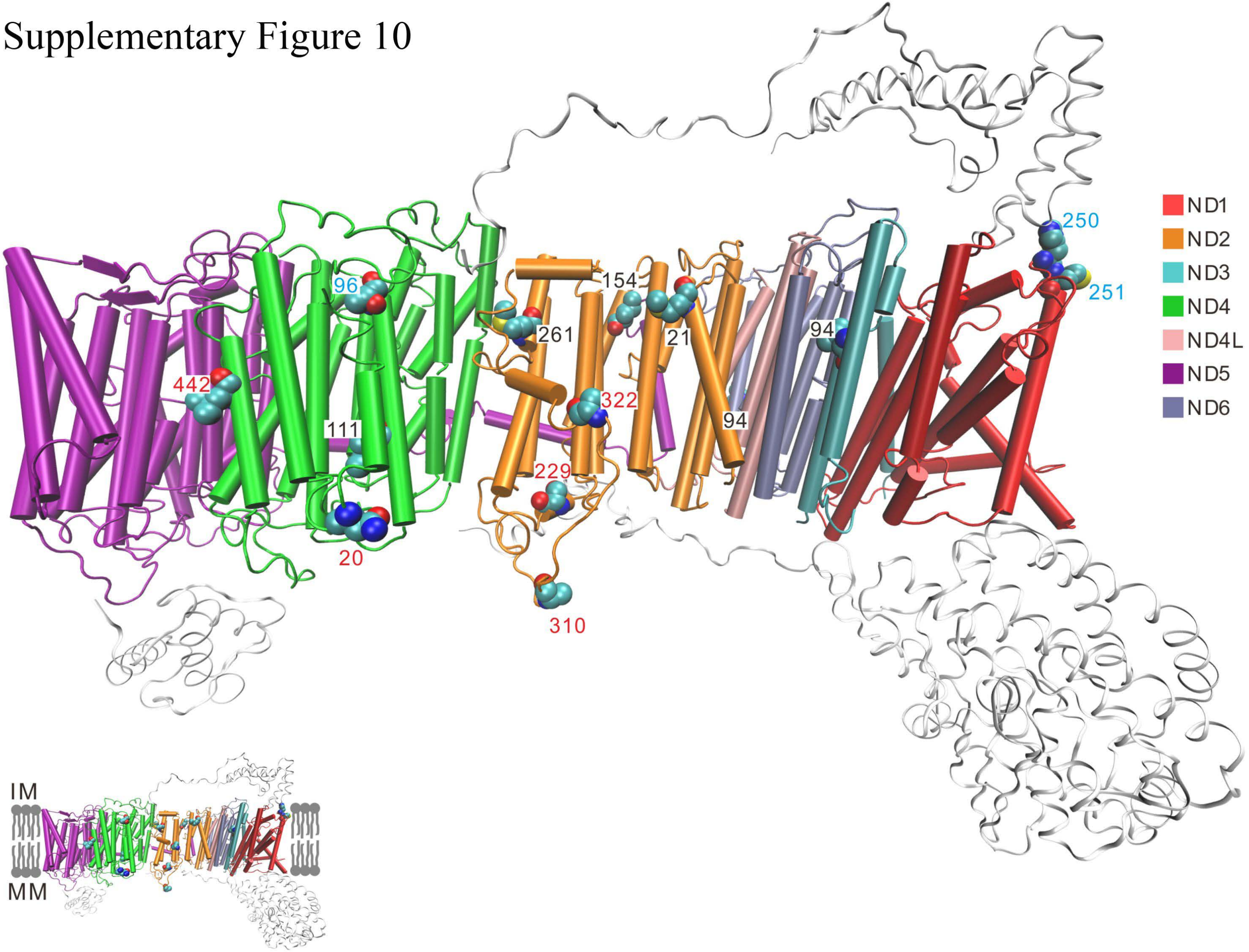

Supplementary Figure 11

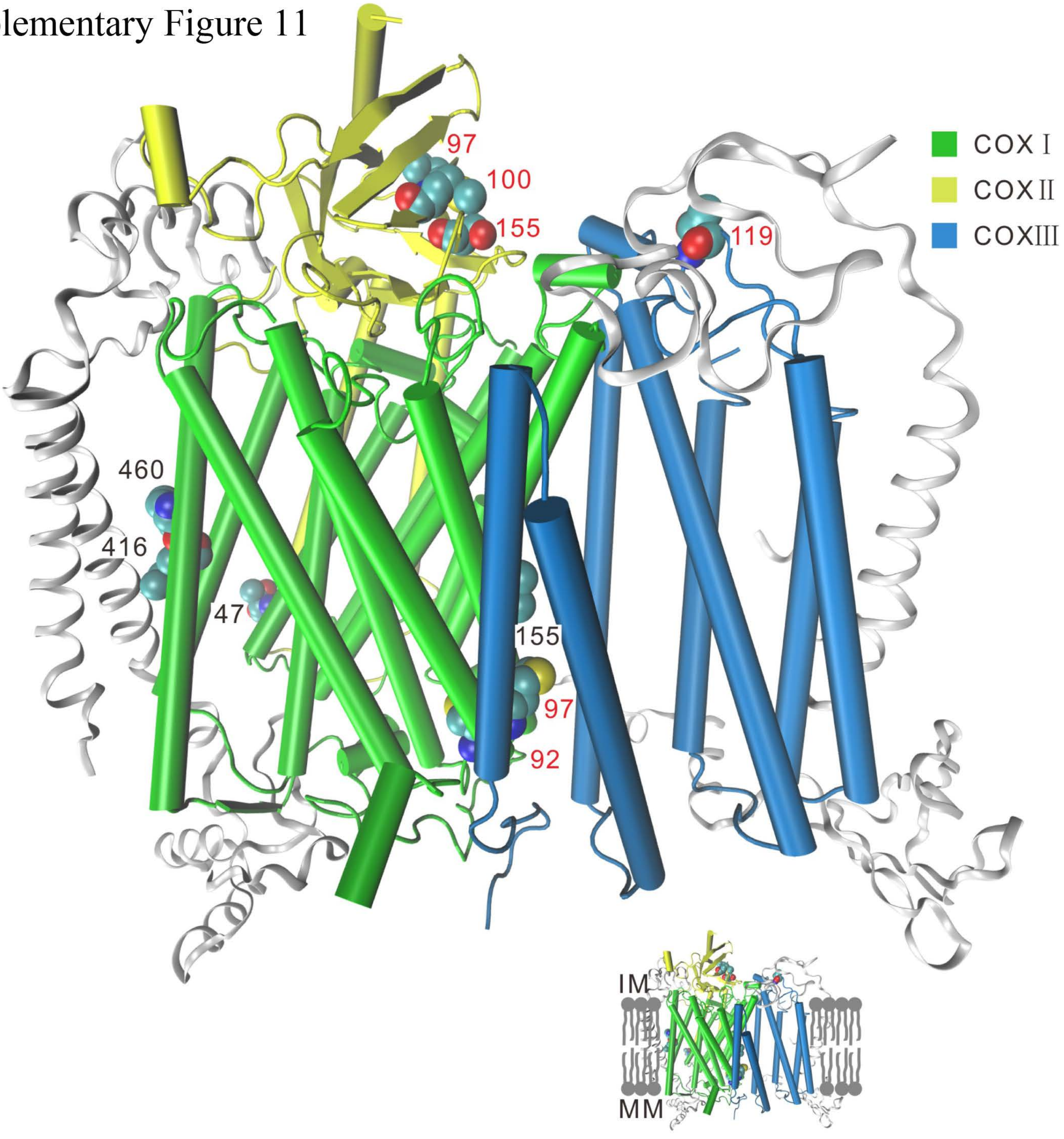

Supplement: Figure S1 — Phylogeny of Anguilliformes used for the detection of positive selection. Deep-sea species were marked in red. [file Presentation_1.pdf]
