# Supplementary material for: Convergent Evolution of Mitochondrial Genes in Deep-Sea Fishes
Source: Front Genet. 2019 Oct 3;10:925. doi: 10.3389/fgene.2019.00925 (PMC6785628; doi:10.3389/fgene.2019.00925)
Supplement: Table S1 — The depth-range of 115 species of 1,042 bony fishes. [file DataSheet_1.pdf]

Supplementary Table 1: The depth-range of 115 species of 1,042 bony fishes.

Species (number of genomes)	Accession numbers	Environment	Depth range	Usually depth
**Perciformes (467)**				
Pagellus bogaraveo (2)	AB305023<br>NC_009502	Marine	?-700m	150-300m
Oreochromis niloticus (8)	GU238433<br>NC_013663<br>GU370126<br>GU477624-GU477628	Fresh water	5-?m	?-20m
Siniperca scherzeri (6)	JN084101<br>NC_015815<br>JQ010985-JQ010988	Fresh water		
Thunnus thynnus (3)	GU256522<br>NC_014052<br>JN086149	Marine	?-985m	?-100m
Micropterus salmoides (2)	DQ536425<br>NC_008106	Fresh water	?-7m	
Oreochromis aureus (4)	GU370125<br>NC_013750<br>GU477629<br>GU477630	Fresh water	5-?m	
Pseudolabrus sieboldi (3)	AP006019<br>NC_009067<br>EU560727	Marine		2-5m
Thunnus alalunga (4)	AB101291<br>NC_005317<br>GU256526<br>JN086151	Marine	?-600m	
Katsuwonus pelamis (4)	AB101290<br>NC_005316<br>GU256527<br>JN086155	Marine	?-260m	
Auxis rochei (4)	AB103467<br>AB103468<br>AB105165	Marine	10-?m	

	NC_005313			
Micropterus dolomieu (3)	AB378749<br>AB378750<br>NC_011361	Fresh water		
Larimichthys polyactis (3)	FJ618559<br>NC_013754<br>GU586227	Marine		
Larimichthys crocea (3)	EU339149<br>NC_011710<br>FJ595214	Marine	10-?m	
Collichthys niveatus (3)	HM219223<br>NC_014263<br>JN678726	Marine		
Collichthys lucidus (3)	HM447239<br>NC_014350<br>JN857362	Marine	?-90m	
Oplegnathus fasciatus (3)	DQ872160<br>NC_010968<br>AP006010	Marine	1-10m	
Epinephelus bruneus (3)	FJ594964<br>NC_013820<br>JQ518289	Marine	20-200m	
Epinephelus lanceolatus (3)	FJ472837<br>NC_011715<br>HQ660062	Marine	4-100m	?-50m
Tropheus duboisi (2)	AP006015<br>NC_009063	Fresh water		
Oreochromis sp. 'red tilapia' (3)	GU477631<br>HM067614<br>NC_014060	Fresh water		
Thunnus obesus (3)	GU256525<br>NC_014059<br>JN086152	Marine	?-250m	

Thunnus albacares (3)	GU256528<br>NC_014061<br>JN086153	Marine	1-250m	1-100m
**Thunnus maccoyii (3)**	**GU256523<br>NC_014101<br>JN086150**	**Marine**	**50-2743m**	
Thunnus orientalis (2)	NC_008455<br>GU256524	Marine	1-200m	
Scarus forsteni (2)	FJ619271<br>NC_011928	Marine	3-30m	
Scarus ghobban (2)	FJ449707<br>NC_011599	Marine	3-36m	
Abudefduf vaigiensis (2)	AP006016<br>NC_009064	Marine	1-15m	
Halichoeres melanurus (2)	AP006018<br>NC_009066	Marine	1-?m	
Halichoeres tenuispinis (2)	EU082205<br>NC_011191	Marine	?-4m	
Odax cyanomelas (2)	AP009130<br>NC_009061	Marine	1-15m	
Anarhichas lupus (2)	EF427916<br>NC_009773	Marine	1-600m	18-110<br>m
Lycodes toyamensis (2)	AP004448<br>NC_004409	Marine		
Pholis crassispina (2)	AP004449<br>NC_004410	Marine	?-5m	
Pagrus major (2)	AP002949<br>NC_003196	Marine		
Pagrus auriga (2)	AB124801<br>NC_005146	Marine	?-170m	
Parargyrops edita (2)	EF107158<br>NC_008616	Fresh water		
Acanthopagrus latus (2)	EF506764	Marine	?-50m	

	NC_010977			
Sciaenops ocellatus (2)	JQ286004<br>NC_016867	Marine	10-?m	
Miichthys miiuy (2)	HM447240<br>NC_014351	Marine	15-100m	
Kuhlia mugil (2)	AP011065<br>NC_013142	Marine	3-18m	
Scorpis lineolata (2)	AP011063<br>NC_013140	Marine	1-30m	
Labracoglossa argentiventris (2)	AP011062<br>NC_013139	Marine		
Kyphosus cinerascens (2)	AP011061<br>NC_013138	Marine	1-45m	
Girella punctata (2)	AP011060<br>NC_013137	Marine		
Microcanthus strigatus (2)	AP006009<br>NC_013182	Marine	1-140m	
Pterocaesio tile (2)	AP004447<br>NC_004408	Marine	?-60m	
Lutjanus argentimaculatus (2)	JN182927<br>NC_016661	Marine	10-120m	
Lutjanus russellii (2)	EF514208<br>NC_010963	Marine	10-80m	20-50m
Lutjanus sebae (2)	FJ824742<br>NC_012737	Marine	5-180m	
Lutjanus malabaricus (2)	FJ824741<br>NC_012736	Marine	12-100m	
Lutjanus bengalensis (2)	FJ171339<br>NC_011275	Marine	10-30m	
Lutjanus rivulatus (2)	AP006000<br>NC_009869	Marine	50-100m	
Lutjanus kasmira (2)	FJ416614	Marine	3-265m	30-150 m

	NC_011578			
Siniperca chuatsi (2)	JF972568<br>NC_015822	Fresh water	10-?m	
Siniperca knerii (2)	JN378751<br>NC_015987	Fresh water		
Coreoperca kawamebari (2)	AP005990<br>NC_009868	Fresh water		
Nannoperca obscura (2)	JF519733<br>NC_015545	Fresh water		
Nannoperca australis (2)	JF519732<br>NC_015542	Fresh water		
Epinephelus akaara (2)	EU043377<br>NC_011113	Marine		
Micropterus floridanus (2)	HQ391897<br>NC_014689	Fresh water		
Lepomis macrochirus (2)	JN389795<br>NC_015984	Fresh water		
Plectropomus leopardus (4)	DQ101270<br>NC_008449<br>JQ420073<br>JQ420074	Marine	3-100m	
Epinephelus coioides (2)	EU043376<br>NC_011111	Marine	?-100m	
Epinephelus septemfasciatus (2)	FJ594966<br>NC_013829	Marine	5-30m	
Anyperodon leucogrammicus (2)	GQ131336<br>NC_012709	Marine	1-80m	5-50m
Hypoplectrus gemma (2)	FJ848375<br>NC_013832	Marine		
Doederleinia berycoides (2)	AP009181<br>NC_009867	Marine	80-200m	
Lethrinus obsoletus (2)	AP009165<br>NC_009855	Marine	?-30m	

Monotaxis grandoculis (2)	AP009166 NC_010957	Marine	1-100m	5-30m
Parapristipoma trilineatum (2)	AP009168 NC_009857	Marine		
Hapalogenys nigripinnis (2)	HM754620 NC_014404	Marine		
Diagramma picta (2)	AP009167 NC_009856	Marine	1-170m	1-50m
Chaetodon auripes (2)	AP006004 NC_009870	Marine		
Heniochus diphreutes (2)	AP006005 NC_009871	Marine	15-210m	
Chaetodontoplus septentrionalis (2)	AP006007 NC_009873	Marine	5-15m	
Centropyge loricula (2)	AP006006 NC_009872	Marine	15-60m	
Sillago sihama (2)	JQ048935 NC_016672	Marine	?-60m	?-20m
Branchiostegus japonicus (2)	EU861052 NC_012904	Marine	30-200m	
Branchiostegus argentatus (2)	EU861054 NC_012907	Marine	51-65m	
Pennahia argentata (2)	HQ890946 NC_015202	Marine	20-140m	40-?m
Nibea albiflora (2)	HQ890947 NC_015205	Marine	25-80m	
Chrysochir aureus (2)	JQ692068 NC_016987	Marine		
Emmelichthys struhsakeri (2)	AP004446 NC_004407	Marine	180-428m	
Monodactylus argenteus (2)	AP009169 NC_009858	Marine		

Enoplosus armatus (2)	AP006008 NC_013181	Marine	?-90m	
Rhynchopelates oxyrhynchus (2)	AP011064 NC_013141	Marine		
Oplegnathus punctatus (2)	AP011066 NC_013143	Marine	3-135m	
Etheostoma radiosum (2)	AY341348 NC_005254	Fresh water		
Percina macrolepida (2)	DQ536430 NC_008111	Fresh water		
Arripis trutta (2)	AP006810 NC_015787	Marine	30-39m	
Morone saxatilis (2)	HM447585 NC_014353	Marine	30-?m	
Spicara maena (2)	AP009164 NC_009854	Marine	30-130m	
Lates calcarifer (2)	DQ010541 NC_007439	Marine	10-40m	
Labracinus cyclophthalmus (2)	AP009125 NC_009054	Marine	2-20m	10-15m
Toxotes chatareus (2)	AP006806 NC_013151	Fresh water		
Branchiostegus albus (2)	EU861053 NC_012905	Marine	30-100m	
Histiopterus typus (2)	AP006807 NC_015786	Marine	40-421m	
Scarus rubroviolaceus (2)	FJ227899 NC_011343	Marine		
Scarus schlegeli (2)	FJ595020 NC_011936	Marine	1-50m	
Chlorurus sordidus (2)	AP006567 NC_006355	Marine	?-50m	

Cymatogaster aggregata (2)	AP009128 NC_009059	Marine;Fresh water	?-146m
Ditrema temminckii (2)	AP009129 NC_009060	Marine	
Astronotus ocellatus (2)	AP009127 NC_009058	Fresh water	
Hypselecara temporalis (2)	AP009506 NC_011168	Fresh water	
Sarotherodon melanotheron (2)	JF894132 NC_015611	Fresh water	3-?m
Oreochromis sp. KM-2006 (2)	AP009126 NC_009057	Fresh water	
Oreochromis mossambicus (2)	AY597335 NC_007231	Fresh water	1-12m
Tylochromis polylepis (2)	AP009509 NC_011171	Fresh water	
Neolamprologus brichardi (2)	AP006014 NC_009062	Fresh water	
Paratilapia polleni (2)	AP009508 NC_011170	Fresh water	
Paretroplus maculatus (2)	AP009504 NC_011177	Fresh water	
Etroplus maculatus (2)	AP009505 NC_011179	Fresh water	
Ptychochromoides katria (2)	AP009507 NC_011169	Fresh water	
Pteragogus flagellifer (2)	EF409976 NC_010205	Marine	
Parajulis poecilepterus (2)	EF192032 NC_009459	Marine	
Cheilinus undulatus (2)	GU296101 NC_013842	Marine	1-100m

Pseudolabrus eoethinus (2)	EU560728<br>NC_012055	Marine		
Halichoeres trimaculatus (2)	EU087704<br>NC_011199	Marine	2-30m	
Amphiprion ocellaris (2)	AP006017<br>NC_009065	Marine	1-15m	3-15m
Amphiprion bicinctus (2)	JQ030887<br>NC_016701	Marine	1-30m	
Auxis thazard (2)	AB105447<br>NC_005318	Marine	50-?m	
Thunnus tonggol (2)	JN086154<br>HQ425780	Marine	10-?m	
Euthynnus alletteratus (2)	AB099716<br>NC_004530	Marine	1-150m	
Scomberomorus cavalla (2)	DQ536428<br>NC_008109	Marine	5-140m	5-15m
Scomberomorus niphonius (2)	GU109281<br>NC_016420	Marine	?-200m	
Gasterochisma melampus (2)	JN086156<br>HQ425781	Marine	200-?m	
Xiphias gladius (2)	AB470301<br>NC_012677	Marine	?-800m	?-550m
Makaira mazara (2)	AB470304<br>NC_012680	Marine	?-200m	100-?m
Makaira indica (4)	AB470305<br>NC_012675<br>KJ510416<br>KJ510417	Marine	?-915m	?-200m
Kajikia audax (2)	NC_012678<br>AB470302	Marine	?-200m	
**Tetrapturus angustirostris (2)**	**AB470303<br>NC_012679**	**Marine**	**?-1830m**	
Istiophorus platypterus (2)	AB470306	Marine	?-200m	30-?m

	NC_012676			
Trichiurus japonicus (2)	EU339148<br>NC_011719	Marine	?-589m	100-350m
Notothenia coriiceps (2)	JF933906<br>NC_015653	Marine	?-550m	
Tridentiger bifasciatus (2)	JN244650<br>NC_015992	Marine		
Gillichthys seta (2)	FJ211846<br>NC_012908	Marine		
Gillichthys mirabilis (2)	FJ211845<br>NC_012906	Marine		
Acanthogobius hasta (3)	AY486321<br>NC_006131<br>JX186192	Marine; Fresh water		
Gymnogobius petschiliensis (2)	AY525784<br>NC_008743	Marine; Fresh water		
Glossogobius olivaceus (2)	JQ001860<br>NC_016664	Marine; Fresh water		
Oxuderces dentatus (2)	JN831381<br>NC_016194	Fresh water		
Boleophthalmus pectinirostris (2)	JN631352<br>NC_016195	Marine; Fresh water		
Trypauchen vagina (2)	JQ027694<br>NC_016693	Marine		
Rhyacichthys aspro (2)	AP004454<br>NC_004414	Marine		
Odontobutis platycephala (2)	DQ010651<br>NC_010199	Fresh water		
Eleotris acanthopoma (2)	AP004455<br>NC_004415	Marine; Fresh water		
**Rachycentron canadum (2)**	**FJ154956**<br>**NC_011219**	**Marine**	**?-1200m**	

Antigonia capros (2)	AP002943<br>NC_003191	Marine	50-900m	100-30<br>0m
Capros aper (2)	AP009159<br>NC_010958	Marine	40-700m	
Petroscirtes breviceps (2)	AP004450<br>NC_004411	Marine	?-10m	
Salarias fasciatus (2)	AP004451<br>NC_004412	Marine	?-8m	
Arcos sp. KU-149 (2)	AP004452<br>NC_004413			
Aspasma minima (2)	AP004453<br>NC_008130	Marine		
Arctoscopus japonicus (2)	AP003090<br>NC_002812	Marine	?-550m	
Zanclus cornutus (2)	AP009162<br>NC_009852	Marine	3-182m	5-182m
Acanthurus leucosternon (2)	EU136032<br>NC_009830	Marine	?-25m	?-25m
Acanthurus lineatus (2)	NC_010108<br>EU273284	Marine	?-15m	1-3m
Zebrasoma flavescens (2)	AP006032<br>NC_009874	Marine	2-46m	3-46m
Naso lopezi (2)	AP009163<br>NC_009853	Marine	6-20m	
Siganus unimaculatus (2)	AP006031<br>NC_013148	Marine	?-30m	1-30m
Siganus fuscescens (2)	EF025185<br>NC_009572	Marine	?-50m	
Luvarus imperialis (2)	AP009161<br>NC_009851	Marine	?-200m	
Platax orbicularis (2)	AP006825<br>NC_013136	Marine	5-30m	

Channa argus (2)	JX978723<br>GU937112	Fresh water		
Channa maculata (2)	JX978724<br>NC_020011	Fresh water		
Chaenocephalus aceratus (2)	JF933907<br>NC_015654	Marine	5-770m	
Chionodraco myersi (2)	DQ526430<br>NC_010689	Marine	200-800m	
Pleuragramma antarctica (2)	JF933905<br>NC_015652	Marine	?-728m	
**Cubiceps pauciradiatus (2)**	**AP006038<br>NC_013150**	**Marine**	**58-1000m**	
Psenes cyanophrys (2)	AP011067<br>NC_013144	Marine	20-550m	
Pampus sp. LY-2009 (2)	EU357803<br>NC_011707			
Hyperoglyphe japonica (2)	AP006037<br>NC_013149	Marine	150-400m	
Sparus aurata (2)	NC_024236<br>LK022698	Marine	1-150m	1-30m
Lutjanus erythropterus (1)	GQ265897	Marine	5-100m	
Epinephelus moara (2)	JQ518290<br>NC_017891	Marine	20-200m	
Argyrosomus japonicus (2)	JQ728563<br>NC_017610	Marine		
Dendrophysa russelii (2)	JQ728562<br>NC_017606	Marine; Fresh water		
Kurtus gulliveri (2)	NC_022477<br>AP006030	Fresh water		
**Anarhichas denticulatus (1)**	**EF427918**	**Marine**	**60-1700m**	**100-900m**
Anarhichas minor (1)	EF427917	Marine	25-600m	100-400m

Scartelaos histophorus (2)	JQ654459 NC_017888	Marine		
Bostrychus sinensis (1)	NC_017880	Marine; Fresh water		
Acanthopagrus schlegelii (2)	JQ746035 NC_018553	Marine	15-?m	
Acentrogobius pflaumii (2)	JX029961 NC_018064	Marine		
Astatotilapia calliptera (2)	JN628855 NC_018560	Fresh water		
Bahaba taipingensis (2)	JX232404 NC_018347	Marine		
Champsocephalus gunnari (2)	GU217678 NC_018340	Marine	0-700m	30-250 m
Cheilochromis euchilus (2)	JN252050 NC_020672			
Chionodraco rastrospinosus (1)	DQ526431	Marine	0-1000m	200-400m
Cynotilapia afra (2)	JN628861 NC_018564	Fresh water		
Dimidiochromis compressiceps (2)	JN628856 NC_018561	Fresh water		
Diplotaxodon limnothrissa (2)	JN628851 NC_018556	Fresh water	20-220m	
Eleginops maclovinus (1)	DQ526429	Marine		
Epinephelus awoara (2)	JX109835 NC_018773	Marine	10-50m	
Epinephelus fuscoguttatus (2)	JX119192 NC_020046	Marine	1-60m	
Genyochromis mento (2)	JN628858 NC_018562	Fresh water	2-25m	
Glossogobius circumspectus (2)	JX536695 NC_018824	Fresh water		

Hapalogenys analis (2)	JX435822 NC_019646	Marine	20-100m	?-100m
Hyporthodus octofasciatus (2)	JX135579 NC_020047	Marine	150-300m	
Lateolabrax maculatus (2)	NC_018045 JQ860109	Marine; Fresh water	5-?m	
Luciogobius platycephalus (2)	JX971538 NC_019811	Marine		
Micropterus salmoides (2)	HQ391896 NC_014686	Fresh water	?-7m	
Nipponocypris sieboldii (2)	NC_008653 AB218898	Fresh water	2-61m	
Odontamblyopus rubicundus (2)	JX891626 NC_019647	Marine		
Opistognathus jacksoniensis (2)	JF911713 NC_017895	Marine	20-?m	30-50m
Pallidochromis tokolosh (1)	JN628859	Fresh water	Fresh water	
Perca flavescens (8)	JX629442-JX629448 NC_019572	Fresh water	?-56m	?-9m
Petrochromis trewavasae (1)	NC_018814	Fresh water		
Petrotilapia nigra (2)	JN628852 NC_018557	Fresh water	3-30m	
Pseudotropheus crabro (2)	JN628854 NC_018559	Fresh water	15-18m	
Repomucenus curvicornis (1)	NC_018567	Marine; Fresh water		
Rhamphochromis esox (2)	JN628860 NC_018563	Fresh water	2-65m	
Sicyopterus japonicus (2)	JX628620 NC_018826	Marine; Fresh water		
Stiphodon alcedo (1)	NC_018054	Fresh water		
Taeniolethrinops praeorbitalis (1)	JN628857	Fresh water		?-50m

Trematocranus placodon (2)	JN628850<br>NC_018555	Fresh water	?-32m
Tridentiger barbatus (2)	JX536694<br>NC_018823		
Tropheus moorii (1)	NC_018815	Fresh water	
**Cypriniformes (330)**			
Carassius auratus (3)	EF483931<br>AB006953<br>NC_002079	Fresh water	?-20m
Carassius auratus auratus (5)	GU086395-GU086397<br>AB111951<br>NC_006580	Fresh water	?-20m
Carassius gibelio (5)	GU138989<br>GU170401<br>NC_014177<br>JF496198<br>6JF496197	Fresh water	
Mylopharyngodon piceus (5)	EU979305-EU979307<br>NC_011141<br>DQ026435	Fresh water	5-30m
Myxocyprinus asiaticus (5)	NC_006401<br>AY526869<br>AB223007<br>AP006764<br>AY986503	Fresh water	
Rhodeus ocellatus (2)	DQ026430<br>NC_011211	Fresh water	
Ctenopharyngodon idella (4)	JQ231115<br>HQ891005<br>NC_010288<br>EU391390	Fresh water	?-30m
Carpiodes carpio (4)	NC_005257<br>AY366087<br>AB126083<br>AP006763	Fresh water	

Rhodeus uyekii (3)	NC_007885 EF483937 DQ155662	Fresh water	
Hypophthalmichthys nobilis (3)	NC_010194 HM162839 EU343733	Fresh water	5-?m
Hypophthalmichthys molitrix (3)	NC_010156 JQ231114 EU315941	Fresh water	?-20m
Carassius auratus x Cyprinus carpio (3)	NC_006136 GU186888 AY694420		
Carassius auratus x Megalobrama amblycephala pentaploid hybrid (3)	NC_013433 GQ903707 HQ401008		
Coreoleuciscus splendidus (3)	DQ347951 NC_007783 EU848546	Fresh water	
Megalobrama amblycephala (3)	NC_010341 DQ026433 EU434747	Fresh water	5-20m
Pseudopungtungia nigra (3)	NC_011161 EU597300 EU332752	Fresh water	
Biwia zezera (3)	NC_008324 AB250108 AB250107	Fresh water	
Minytrema melanops (3)	DQ536432 AB242166 NC_008113	Fresh water	
Cobitis choii (3)	NC_010649 EU656112 EU333980	Fresh water	
Cobitis striata (3)	AB054125 NC_004695 AP010782	Fresh water	
Misgurnus anguillicaudatus (3)	NC_011209 HM856629 DQ026434	Fresh water	5-?m

Koreocobitis naktongensis (3)	NC_015798<br>HM535625<br>JN607252	Fresh water	
Gyrinocheilus aymonieri (3)	AB242164<br>NC_008672<br>DQ026432	Fresh water	
Acheilognathus yamatsutae (2)	NC_013712<br>EF483936	Fresh water	
Acheilognathus macropterus (2)	NC_013711<br>EF483935	Fresh water	
Acheilognathus signifer (2)	NC_013702<br>EF483930	Fresh water	
Acheilognathus typus (2)	AB239602<br>NC_008668	Fresh water	
Acheilognathus koreensis (2)	EF483932<br>NC_013704	Fresh water	
Acheilognathus intermedia (2)	NC_013705<br>EF483933	Fresh water	
Acheilognathus somjinensis (2)	NC_014882<br>FJ515921	Fresh water	
Cyprinus carpio (2)	NC_001606<br>AP009047	Fresh water	
Cyprinella spiloptera (2)	DQ536422<br>NC_008103	Fresh water	
Cyprinella lutrensis (2)	AB070206<br>NC_008643	Fresh water	
Campostoma anomalum (2)	DQ536421<br>NC_008102	Fresh water	
Phoxinus eos (2)	AP009151<br>NC_015364	Fresh water	
Phoxinus steindachneri (2)	NC_015357<br>AP009148	Fresh water	
Notemigonus crysoleucas (2)	NC_008646<br>AB127393	Fresh water	10-?m
Labeo senegalensis (2)	NC_008657<br>AB238968	Fresh water	
Labeo bata (2)	NC_015193<br>AP011198	Fresh water	
Hypentelium nigricans (2)	NC_008676<br>AB242169	Fresh water	
Xyrauchen texanus (2)	NC_010234<br>EU265776	Fresh water	

Erimyzon oblongus (2)	NC_013064<br>AP011228	Fresh water	
Cycleptus elongatus (2)	NC_008645<br>AB126082	Fresh water	
Catostomus commersonii (2)	NC_008647<br>AB127394	Fresh water	?-30m
Moxostoma poecilurum (2)	NC_008674<br>AB242167	Fresh water	
Moxostoma congestum (2)	NC_013074<br>AP009317	Fresh water	
Ictiobus bubalus (2)	AP009316<br>NC_013071	Fresh water	
Cobitis sinensis (2)	NC_007229<br>AY526868	Fresh water	
Cobitis takatsuensis (2)	NC_015306<br>AP009306	Fresh water	
Niwaella delicata (2)	NC_015307<br>AP009308	Fresh water	
Acantopsis choirorhynchos (2)	AB242161<br>NC_008669	Fresh water	
Pangio anguillaris (2)	NC_008675<br>AB242168	Fresh water	
Misgurnus nikolskyi (2)	AB242171<br>NC_008678	Fresh water	
Leptobotia mantschurica (2)	NC_008677<br>AB242170	Fresh water	
Chromobotia macracanthus (2)	NC_008671<br>AB242163	Fresh water	
Lefua echigonia (2)	AB054126<br>NC_004696	Fresh water	
Barbatula toni (2)	NC_008670<br>AB242162	Fresh water	
Schistura balteata (2)	NC_008679<br>AB242172	Fresh water	
Vaillantella maassi (2)	NC_008680<br>AB242173	Fresh water	
Labeo batesii (2)	NC_008656<br>AB238967	Fresh water	
Catla catla (2)	NC_016892<br>JQ087872	Fresh water	5-?m
Alburnus alburnus (2)	NC_008659<br>AB239593	Fresh water	1-?m
Tinca tinca (2)	NC_008648<br>AB218686	Fresh water	1-?m

Opsariichthys bidens (2)	NC_008744<br>DQ367044	Fresh water	
Opsariichthys uncirostris (2)	AB218897<br>NC_008652	Fresh water	
Barbonymus gonionotus (2)	NC_008655<br>AB238966	Fresh water	15-?m
Tanichthys albonubes (2)	AP011397<br>NC_015539	Fresh water	
Chondrostoma lemmingii (2)	DQ536427<br>NC_008108	Fresh water	
Rasbora daniconius (2)	AP011285<br>NC_015527	Fresh water	
Rasbora vaterifloris (2)	AP011432<br>NC_015531	Fresh water	
Pseudorasbora parva (2)	NC_015614<br>JF802126	Fresh water	
Pseudorasbora pumila (2)	NC_008665<br>AB239599	Fresh water	
Carassius carassius (2)	AY714387<br>NC_006291	Fresh water	5-?m
Gymnocypris przewalskii (2)	NC_008661<br>AB239595	Fresh water	
Tribolodon nakamurai (2)	NC_008651<br>AB218896	Fresh water	
Carassius auratus x Cyprinus carpio x Carassius cuvieri (2)	NC_006387<br>AY771781		
Carassius auratus x Megalobrama amblycephala tetraploid hybrid (2)	GQ903705<br>NC_013430		
Carassius auratus x Megalobrama amblycephala triploid hybrid (2)	GQ903706<br>NC_013431		
natural gynogenetic Carassius auratus red var. (2)	NC_012980<br>GQ303444		
Hemibarbus mylodon (2)	DQ345787<br>NC_007786	Fresh water	
Hemibarbus longirostris (2)	DQ347952<br>NC_007784	Fresh water	
Hemibarbus labeo (2)	DQ347953<br>NC_007785	Fresh water	
Hemibarbus barbus (2)	AB070241	Fresh water	

	NC_008644		
Rhynchocypris lagowskii (2)	NC_015354<br>AP009147	Fresh water	
Pseudaspius leptocephalus (2)	NC_008681<br>AP009058	Fresh water	
Chanodichthys mongolicus (2)	NC_008683<br>AP009060	Fresh water	
Aphyocypris chinensis (2)	AB218688<br>NC_008650	Fresh water	
Distoechodon tumirostris (2)	NC_011208<br>DQ026431	Fresh water	10-20m
Psilorhynchus homaloptera (2)	DQ026436<br>NC_011210	Fresh water	
Psilorhynchus sucatio (2)	AP011288<br>NC_015532	Fresh water	
Gobiobotia macrocephala (2)	NC_014877<br>FJ515918	Fresh water	
Gobiobotia brevibarba (2)	FJ515919<br>NC_014878	Fresh water	
Pungtungia herzi (2)	NC_008664<br>AB239598	Fresh water	
Sarcocheilichthys variegatus microoculus (2)	NC_004694<br>AB054124	Fresh water	
Rhodeus suigensis (2)	EF483934<br>NC_013709	Fresh water	
Gnathopogon elongatus (2)	AB218687<br>NC_008649	Fresh water	
Pelecus cultratus (2)	NC_008663<br>AB239597	Fresh water	20-?m
Ischikauia steenackeri (2)	NC_008667<br>AB239601	Fresh water	
Hemigrammocypris rasborella (2)	NC_015548<br>AP011422	Fresh water	
Xenocypris argentea (2)	NC_008682<br>AP009059	Fresh water	
Xenocypris davidi (2)	GQ289558<br>NC_013072	Fresh water	
Puntius tetrazona (2)	NC_010110<br>EU287909	Fresh water	
Esomus metallicus (2)	NC_008660<br>AB239594	Fresh water	
Phenacobius mirabilis (2)	DQ536431<br>NC_008112	Fresh water	

Culter alburnus (2)	NC_013616<br>GU190362	Fresh water	
Pseudogobio esocinus (2)	AP009310<br>NC_013759	Fresh water	
Raiamas senegalensis (2)	AP010780<br>NC_013764	Fresh water	
Raiamas guttatus (2)	NC_015547<br>AP011222	Fresh water	
Danio dangila (2)	AP011235<br>NC_015525	Fresh water	
Sinocyclocheilus grahami (2)	GQ148557<br>NC_013189	Fresh water	
Sinocyclocheilus altishoulderus (2)	NC_013186<br>FJ984568	Fresh water	
Notropis stramineus (2)	DQ536429<br>NC_008110	Fresh water	
Yaoshanicus arcus (2)	NC_015540<br>AP011398	Fresh water	
diploid Megalobrama amblycephala x Xenocypris davidi (2)	GU949545<br>NC_013994		
diploid Xenocypris davidi x Megalobrama amblycephala (2)	HQ651067<br>NC_015838		
triploid Megalobrama amblycephala x Xenocypris davidi (2)	NC_013995<br>GU949546		
Barilius bendelisis (2)	AP011433<br>NC_015533	Fresh water	
Luciosoma bleekeri (2)	NC_015541<br>AP011399	Fresh water	
Horadandia atukorali (2)	AP011400<br>NC_015544	Fresh water	
Leptobarbus hoevenii (2)	NC_015528<br>AP011286	Fresh water	3-?m
Trigonostigma espei (2)	NC_015535<br>AP011449	Fresh water	
Salmostoma bacaila (2)	NC_015549<br>AP011223	Fresh water	
Pseudopungtungia tenuicorpus (2)	FJ515917<br>NC_014873	Fresh water	
Gila robusta (2)	DQ536424<br>NC_008105	Fresh water	

Gila conspersa (2)	AP009315<br>NC_013761	Fresh water	
Barbus barbus (2)	AB238965<br>NC_008654	Fresh water	10-?m
Barbus trimaculatus (2)	AB239600<br>NC_008666	Fresh water	
Gobio gobio (2)	AB239596<br>NC_008662	Fresh water	
Procypris rabaudi (2)	EU082030<br>NC_011192	Fresh water	
Mylocheilus caurinus (2)	AP010779<br>NC_013763	Fresh water	
Microdevario nana (2)	AP011402<br>NC_015546	Fresh water	
Aspidoparia morar (2)	AP011335<br>NC_015536	Fresh water	
Nicholsicypris normalis (2)	NC_015538<br>AP011396	Fresh water	
Microphysogobio koreensis (2)	FJ515920<br>NC_014880	Fresh water	
Paralaubuca typus (2)	NC_015194<br>AP011211	Fresh water	
Macrochirichthys macrochirus (2)	NC_015551<br>AP011234	Fresh water	
Sinogastromyzon puliensis (2)	NC_011922<br>FJ605359	Fresh water	?-10m
Homaloptera leonardi (2)	AB242165<br>NC_008673	Fresh water	
Formosania lacustris (2)	NC_001727<br>AP010774	Fresh water	1-10m
Sewellia lineolata (2)	AP011292<br>NC_015534	Fresh water	
Labeo rohita (1)	JQ231111	Fresh water	5-?m
Labeo calbasu (1)	JQ231113	Fresh water	10-10m
Luciosoma setigerum (1)	AP011423	Fresh water	
Boraras maculatus (1)	AP011420	Fresh water	
Leptocypris sp. CBM ZF 11419 (1)	AP011428		
Danio rerio (1)	NC_002333	Fresh water	
Carassius cuvieri (1)	NC_010768	Fresh water	
Trigonostigma heteromorpha (1)	AP011421	Fresh water	
Cirrhinus mrigala (1)	JQ231112	Fresh water	5-?m

Danionella mirifica (1)	AP011424	Fresh water
Microrasbora erythromicron (1)	AP011419	Fresh water
Rasbora cephalotaenia (1)	AP011430	Fresh water
Paedocypris sp. Banka Is. (1)	AP011429	
Paedocypris progenetica (1)	AP011287	Fresh water
Triplophysa stoliczkai (1)	JQ663847	Fresh water
Koreocobitis rotundicaudata (1)	JN607253	Fresh water
Pethia ticto (2)	NC_008658 AB238969	Fresh water
**Tetraodontiformes (208)**		
Takifugu rubripes (3)	NC_004299 AJ421455 AP006045	Marine;Fresh water
Takifugu obscurus (3)	AP009527 EF672695 NC_011626	Marine;Fresh water
Tetraodon nigroviridis (3)	NC_007176 DQ019313 AP006046	Fresh water
Takifugu xanthopterus (2)	AP009533 NC_011632	Marine
Takifugu pardalis (2)	AP009528 NC_011627	Marine
Takifugu fasciatus (2)	GQ409967 NC_013087	
Takifugu niphobles (2)	AP009526 NC_011625	Marine
Takifugu snyderi (2)	AP009531 NC_011630	Marine
Takifugu porphyreus (2)	AP009529 NC_011628	Marine
Takifugu chinensis (2)	AP009534 NC_011633	Marine
Takifugu vermicularis (2)	AP009532 NC_011631	Marine
Takifugu stictonotus (2)	AP009530 NC_011629	Marine
Takifugu exascurus (2)	AP009540	Marine

	NC_011622			
Takifugu chrysops (2)	AP009525<br>NC_011624	Marine		
Takifugu oblongus (2)	AP009535<br>NC_011634	Marine		
Takifugu ocellatus (2)	AP009536<br>NC_011635	Marine;Fresh water		
Takifugu poecilonotus (2)	AP009539<br>NC_011621	Marine;Fresh water		
Tetraodon biocellatus (2)	AP011921<br>NC_015358	Fresh water		
Tetraodon miurus (2)	AP011922<br>NC_015361	Fresh water		
Tetraodon mbu (2)	AP011923<br>NC_015363	Fresh water		
Mola mola (2)	NC_005836<br>AP006238	Marine	30-480m	
Masturus lanceolatus (2)	NC_005837<br>AP006239	Marine	?-670m	
Ranzania laevis (2)	NC_007887<br>AP006047	Marine	1-140m	
Lagocephalus sceleratus (2)	AP011932<br>NC_015340	Marine	18-100m	
Lagocephalus lunaris (2)	GQ461750<br>NC_013360	Marine		
Lagocephalus wheeleri (2)	AP009538<br>NC_011637	Marine		
Lagocephalus laevigatus (2)	AP011934<br>NC_015345	Marine	10-180m	
Lagocephalus lagocephalus (2)	AP011933<br>NC_015343	Marine	10-476m	10-100 m
Colomesus asellus (2)	AP011909<br>NC_015368	Fresh water		
Colomesus psittacus (2)	AP011910<br>NC_015370	Marine;Fresh water	?-40m	
Sphoeroides pachygaster (2)	AP006745<br>NC_010960	Marine	50-480m	50-250 m
Sphoeroides testudineus (2)	AP011916<br>NC_015346	Marine	?-48m	
Sphoeroides parvus (2)	AP011914<br>NC_015341	Marine		
Sphoeroides annulatus (2)	AP011915<br>NC_015344	Marine	?-11m	

Arothron hispidus (2)	AP011930<br>NC_015336	Marine	3-50m	
Arothron firmamentum (2)	AP006742<br>NC_010975	Marine	10-360m	
Arothron manilensis (2)	AP011929<br>NC_015371	Marine	2-20m	
Canthigaster coronata (2)	AP006743<br>NC_010978	Marine	6-165m	
Canthigaster jactator (2)	AP011911<br>NC_015372	Marine	1-30m	
Canthigaster valentini (2)	AP011912<br>NC_015337	Marine	1-55m	
Stephanolepis cirrhifer (2)	NC_003177<br>AP002952	Marine		
Monacanthus chinensis (2)	NC_011925<br>AP009219	Marine	3-12m	
Brachaluteres ulvarum (2)	NC_011940<br>AP009215	Marine	25-30m	
Chaetodermis penicilligerus (2)	AP009216<br>NC_011951	Marine		
Meuschenia hippocrepis (2)	NC_011956<br>AP009218	Marine	?-120m	
Nelusetta ayraudi (2)	NC_011921<br>AP009220	Marine	?-360m	?-200m
Paraluteres prionurus (2)	NC_011929<br>AP009222	Marine	1-25m	
Pervagor janthinosoma (2)	NC_011932<br>AP009224	Marine	8-20m	
Acreichthys tomentosus (2)	NC_011950<br>AP009213	Marine	2-15m	
Rudarius ercodes (2)	NC_011924<br>AP009227	Marine		
Oxymonacanthus longirostris (2)	NC_011927<br>AP009221	Marine	4-30m	
Amanses scopas (2)	NC_011952<br>AP009214	Marine	3-18m	
Pseudalutarius nasicornis (2)	NC_011955<br>AP009226	Marine	1-55m	
Cantherhines pardalis (2)	NC_011325<br>AP009184	Marine	2-20m	
Aluterus scriptus (2)	NC_011323<br>AP009183	Marine	3-120m	3-20m
Thamnaconus modestus (2)	NC_011327<br>AP009185	Marine		

Eubalichthys mosaicus (2)	NC_011953 AP009217	Marine	-140m
Paramonacanthus choirocephalus (2)	NC_011931 AP009223	Marine	?-8m
Pseudomonacanthus peroni (2)	NC_011933 AP009225	Marine	
Acanthaluteres brownii (2)	NC_011947 AP009212	Marine	?-50m
Triodon macropterus (2)	NC_009859 AP009170	Marine	50-300m
Torquigener pleurogramma (2)	AP011926 NC_015367	Marine	?-30m
Torquigener hypselogeneion (2)	AP011927 NC_015332	Marine	18-22m
Tetractenos glaber (2)	AP011935 NC_015347	Marine	
Carinotetraodon lorteti (2)	AP011918 NC_015350	Fresh water	
Carinotetraodon salivator (2)	AP011919 NC_015352	Fresh water	1-2m
Auriglobus modestus (2)	AP011917 NC_015348	Fresh water	
Polyspina piosae (2)	AP011913 NC_015339	Marine	
Marilyna darwinii (2)	AP011937 NC_015351	Marine	
Monotrete cochinchinensis (2)	AP011925 NC_015366	Fresh water	
Chelonodon pleurospilus (2)	AP011928 NC_015369	Marine	
Chelonodon patoca (2)	AP009541 NC_011623	Marine;Fresh water	4-?m
Tylerius spinosissimus (2)	AP011939 NC_015356	Marine	250-435m
Canthigaster rivulata (2)	AP006744 NC_010979	Marine	?-350m
Omegophora armilla (2)	AP011936 NC_015349	Marine	?-146m
Pelagocephalus marki (2)	AP011938 NC_015353	Marine	
Chilomycterus reticulatus (2)	NC_011331 AP009188	Marine	20-100m
Triacanthodes anomalus (2)	NC_009861 AP009172	Marine	

Macrorhamphosodes uradoi (2)	NC_009860 AP009171	Marine	50-675m	
Triacanthus biaculeatus (2)	NC_009863 AP009174	Marine	?-60m	
Trixiphichthys weberi (2)	NC_009862 AP009173	Marine	?-65m	
Ostracion immaculatus (2)	NC_009865 AP009176	Marine		
Lactoria diaphana (2)	NC_011330 AP009187	Marine	8-50m	
Kentrocapros aculeatus (2)	NC_009864 AP009175	Marine	100-200m	
Anoplocapros lenticularis (2)	NC_011319 AP009186	Marine	10-220m	
Balistapus undulatus (2)	NC_011946 AP009203	Marine	2-50m	
Balistoides conspicillum (2)	NC_011934 AP009205	Marine	1-75m	
Melichthys vidua (2)	NC_011937 AP009207	Marine	4-60m	
Pseudobalistes flavimarginatus (2)	NC_011939 AP009209	Marine	2-50m	
Xanthichthys auromarginatus (2)	NC_011945 AP009211	Marine	8-150m	24-147 m
Odonus niger (2)	NC_011938 AP009208	Marine	5-40m	
Abalistes stellaris (2)	NC_011943 AP009202	Marine		
Balistes vetula (2)	NC_011948 AP009204	Marine	2-275m	3-30m
Xenobalistes tumidipectoris (2)	NC_011321 AP009182	Marine		
Canthidermis maculata (2)	NC_011935 AP009206	Marine	1-110m	
Rhinecanthus aculeatus (2)	NC_011941 AP009210	Marine	?-50m	
Sufflamen fraenatum (2)	NC_004416 AP004456	Marine	8-186m	18-?m
Diodon holocanthus (2)	NC_009866 AP009177	Marine	2-200m	2-35m
Scobinichthys granulatus (2)	NC_011926 AP009228	Marine		
Arothron mappa (1)	AP011931	Marine	4-30m	

Leiodon cutcutia (2)	NC_015365<br>AP011924	Fresh water		
Pao palembangensis (2)	NC_015355<br>AP011920	Fresh water		
Torquigener brevipinnis (2)	AP009537<br>NC_011636	Marine	20-100m	
**Salmoniformes (149)**				
Coregonus lavaretus (81)	NC_002646<br>AB034824<br>JQ661382-JQ661397<br>JQ661419-JQ661481	Marine		
Coregonus oxyrinchus (21)	JQ661398-JQ661418	Marine;Fresh water		
Oncorhynchus masou formosanus (2)	DQ858456<br>NC_008745	Fresh water		
Oncorhynchus mykiss (6)	ONHMTCG<br>NC_001717<br>DQ288268-DQ288271	Marine;Fresh water	?-200m	
Coregonus clupeaformis (6)	JQ661482-JQ661487	Marine;Fresh water	18-128m	
Salmo salar (3)	U12143<br>NC_001960<br>AF133701	Marine;Fresh water	?-210m	?-10m
Oncorhynchus gorbuscha (2)	NC_010959<br>EF455489	Marine;Fresh water	?-250m	
Oncorhynchus kisutch (2)	NC_009263<br>EF126369	Marine;Fresh water	?-250m	
Oncorhynchus masou masou (2)	NC_008747<br>DQ864465	Marine;Fresh water	?-200m	
Oncorhynchus masou ishikawae (2)	NC_008746<br>DQ864464	Marine;Fresh water	?-200m	
Oncorhynchus masou 'Biwa' (2)	EF105342<br>NC_009262			
Oncorhynchus clarkii henshawi (2)	AY886762<br>NC_006897			
Oncorhynchus nerka (2)	NC_008615<br>EF055889	Marine;Fresh water	?-250m	

Oncorhynchus tshawytscha (2)	NC_002980 AF392054	Marine;Fresh water	?-375m
Salvelinus alpinus (2)	NC_000861 AF154851	Marine;Fresh water	30-70m
Salvelinus fontinalis (2)	NC_000860 AF154850	Marine;Fresh water	15-27m
Hucho taimen (2)	NC_016426 HQ897271	Fresh water	
Hucho bleekeri (2)	NC_015995 HM804473	Fresh water	
Thymallus thymallus (2)	NC_012928 FJ853655	Fresh water	15-?m
Thymallus arcticus (2)	NC_012929 FJ872559	Fresh water	30-?m
Oncorhynchus keta (1)	AP010773	Marine;Fresh water	?-250m
Salmo trutta trutta (1)	NC_010007	Marine;Fresh water	?-10m
**Anguilliformes (64)**			
Anguilla anguilla (1)	NC_006531	Marine;Fresh water	?-700m
Anguilla mossambica (1)	NC_006542	Marine;Fresh water	
Anguilla japonica (1)	NC_002707	Marine;Fresh water	1-400m
Anguilla marmorata (1)	NC_006540	Marine;Fresh water	1-400m
Anguilla reinhardtii (1)	NC_006546	Marine;Fresh water	?-1m
Anguilla bicolor pacifica (1)	NC_006535	Marine;Fresh water	
Anguilla bicolor bicolor (1)	NC_006534	Marine;Fresh water	
Anguilla australis australis (1)	NC_006532	Marine;Fresh water	
Anguilla australis schmidti (1)	NC_006533	Fresh water	
Anguilla malgumora (1)	NC_006536	Marine;Fresh water	
Anguilla bengalensis labiata (1)	NC_006543	Marine;Fresh water	
Anguilla obscura (1)	NC_006545	Marine;Fresh water	
Anguilla interioris (1)	NC_006539	Marine;Fresh water	
Anguilla nebulosa nebulosa (1)	NC_006544	Marine;Fresh water	
Anguilla dieffenbachii (1)	NC_006538	Marine;Fresh water	
Anguilla luzonensis (2)	NC_013435 NC_011575	Marine;Fresh water	
Anguilla rostrata (1)	NC_006547	Marine;Fresh water	?-464m

Anguilla celebesensis (1)	NC_006537	Marine;Fresh water		
Anguilla megastoma (1)	NC_006541	Marine;Fresh water		
Conger myriaster (1)	NC_002761	Marine	320-830m	
Paraconger notialis (1)	NC_013630	Marine	25-50m	25-50m
Ariosoma shiroanago (1)	NC_013632	Marine		
Heteroconger hassi (1)	NC_013629	Marine	7-45m	
**Nemichthys scolopaceus (1)**	**NC_013620**	**Marine**	**?-3656m**	**100-1000m**
**Avocettina infans (1)**	**NC_013624**	**Marine**	**?-4580m**	**1200-2000m**
**Labichthys carinatus (1)**	**NC_013626**	**Marine**	**?-2000m**	
**Serrivomer beanii (1)**	**NC_013627**	**Marine**	**?-5998m**	
**Serrivomer sector (1)**	**NC_013436**	**Marine**	**?-3243m**	**305-?m**
**Stemonidium hypomelas (1)**	**NC_013628**	**Marine**	**500-1229m**	
**Ilyophis brunneus (2)**	**NC_013634 AP010848**	**Marine**	**450-3120m**	
**Simenchelys parasitica (2)**	**NC_013605 AP010849**	**Marine**	**136-2620m**	**500-1800m**
**Synaphobranchus kaupii (1)**	**NC_005805**	**Marine**	**120-4800m**	**400-2200m**
Coloconger cadenati (1)	NC_013606	Marine	270-600m	
**Derichthys serpentinus (1)**	**NC_013611**	**Marine**	**?-2000m**	**200-700m**
**Nessorhamphus ingolfianus (1)**	**NC_013608**	**Marine**	**?-1800m**	
Cynoponticus ferox (1)	NC_013617	Marine	10-100m	
Muraenesox bagio (1)	NC_013614	Marine	?-100m	
Ophisurus macrorhynchos (1)	NC_005802	Marine		
Myrichthys maculosus (1)	NC_013635	Marine	?-262m	
Gymnothorax kidako (1)	NC_004417	Marine	2-350m	
Rhinomuraena quaesita (1)	NC_013610	Marine	1-57m	
Anarchias sp. Ansp (1)	NC_013613			
Kaupichthys hyoproroides (1)	NC_013607	Marine	1-95m	
Robinsia catherinae (2)	NC_013633 AP010846	Marine	140-243m	
**Thalassenchelys sp. Tht2 (2)**	**NC_013618 AP010867**		**>1000m**	
Myroconger compressus (1)	NC_013631	Marine		
Moringua edwardsi (1)	NC_013622	Marine		
Moringua microchir (1)	NC_013602	Marine	3-20m	
Pythonichthys microphthalmus (1)	NC_013601	Marine	40-150m	

**Saccopharynx lavenbergi (2)**	**NC_005298 AB047825**	**Marine**	**2000-3000m**	**2000-3000m**
**Monognathus jesperseni (1)**	**NC_013612**	**Marine**	**?-3000m**	
**Cyema atrum (1)**	**NC_013609**	**Marine**	**330-5100m**	
**Eurypharynx pelecanoides (1)**	**NC_005299**	**Marine**	**500-7625m**	**1200-1400m**
**Hoplunnis punctata (1)**	**NC_013623**	**Marine**		
Leptocephalus sp. 'type II larva' (1)	NC_013615			
**Nettastoma parviceps (1)**	**NC_013625**	**Marine**	**60-1190m**	
Facciolella oxyrhyncha (1)	NC_013621	Marine	30-731m	
Protoanguilla palau (1)	AP011809	Marine	35-?m	
**Clupeiformes (109)**				
Alosa sapidissima (2)	HQ331537 NC_014690	Marine;Fresh water	?-250m	
Alosa pseudoharengus (2)	NC_009576 AP009132	Marine;Fresh water	5-145m	56-110
Alosa alosa (2)	NC_009575 AP009131	Marine;Fresh water	?-300m	
Ethmalosa fimbriata (2)	NC_009582 AP009138	Marine;Fresh water	?-50m	
Brevoortia tyrannus (2)	NC_014266 AP009618	Marine	?-50m	
Clupea harengus (2)	NC_009577 AP009133	Marine	?-364m	?-200m
Clupea pallasii (2)	NC_009578 AP009134	Marine;Fresh water	?-475m	
Sardinops melanostictus (2)	NC_002616 AB032554	Marine	?-200m	
Sardina pilchardus (2)	NC_009592 AP009233	Marine;Fresh water	10-100m	25-100m
Sardinella maderensis (2)	NC_009587 AP009143	Marine	?-80m	
Sprattus sprattus (2)	NC_009593 AP009234	Marine	10-150m	
Clupeonella cultriventris (2)	NC_015109 AP009615	Marine;Fresh water	10-13m	
Etrumeus teres (1)	NC_009583	Marine	?-150m	
Spratelloides delicatulus (2)	NC_009588 AP009144	Marine	?-50m	

Spratelloides gracilis (2)	NC_009589<br>AP009145	Marine	10-?m
Jenkinsia lamprotaenia (2)	NC_006917<br>AP006230	Marine	?-50m
Jenkinsia sp. CL54 (2)	NC_015076<br>AP009496		
Dorosoma cepedianum (2)	DQ536426<br>NC_008107	Marine;Fresh water	?-33m
Dorosoma petenense (2)	NC_009580<br>AP009136	Marine;Fresh water	?-15m
Nematalosa japonica (2)	NC_009586<br>AP009142	Marine	
Engraulis japonicus (2)	NC_003097<br>AB040676	Marine	?-400m
Engraulis encrasicolus (2)	NC_009581<br>AP009137	Marine	?-400m
Amazonsprattus scintilla (2)	NC_014265<br>AP009617	Fresh water	
Lycengraulis grossidens (2)	NC_014279<br>AP011563	Marine;Fresh water	?-40m
Anchoviella sp. LBP 2297 (2)	NC_014269<br>AP011557		
Coilia nasus (2)	NC_009579<br>AP009135	Marine;Fresh water	?-50m
Coilia lindmani (2)	NC_014271<br>AP011558	Fresh water	
Coilia reynaldi (2)	NC_014276<br>AP011559	Marine;Fresh water	?-50m
Thryssa baelama (2)	NC_014264<br>AP009616	Marine	?-50m
Lycothrissa crocodilus (2)	NC_014277<br>AP011562	Fresh water	
Chirocentrus dorab (2)	NC_006913<br>AP006229	Marine	?-120m
Ilisha elongata (2)	NC_009585<br>AP009141	Marine	5-?m
Ilisha africana (2)	NC_009584<br>AP009140	Marine	?-25m
Pellona flavipinnis (2)	NC_014268<br>AP009619	Fresh water	
Odaxothrissa vittata (1)	NC_009590	Fresh water	
Pellonula leonensis (2)	NC_009591<br>AP009232	Marine;Fresh water	

Denticeps clupeoides (2)	NC_007889 AP007276	Fresh water		
Sundasalanx mekongensis (2)	NC_006919 AP006232	Fresh water		
Stolephorus chinensis (1)	AP011566	Marine	?-50m	
Stolephorus waitei (1)	AP011567	Marine;Fresh water	?-50m	
Konosirus punctatus (1)	NC_016694	Marine		
Escualosa thoracata (1)	NC_016706	Marine;Fresh water	?-50m	
Sprattus muelleri (1)	NC_016669	Marine	28-110m	
Sprattus antipodum (1)	NC_016673	Marine	?-72m	
Gudusia chapra (1)	NC_016715	Fresh water		
Harengula jaguana (1)	NC_016667	Marine	?-22m	1-5m
Ethmidium maculatum (1)	NC_016710	Marine	?-50m	
Clupeoides borneensis (1)	NC_016709	Fresh water		
Clupeoides sp. Chao Phraya (1)	NC_016714			
Ehirava fluviatilis (1)	NC_016717	Marine;Fresh water		
Potamalosa richmondia (1)	NC_016674	Marine;Fresh water		
Microthrissa congica (1)	NC_016695	Fresh water		
Odaxothrissa losera (2)	NC_016678 AP011595			
Rhynchocypris percnurus (2)	NC_015360 AP009149			
Hyperlophus vittatus (1)	NC_016671	Marine	10-13m	
Clupeichthys perakensis (1)	NC_016705	Fresh water		
Clupeichthys aesarnensis (1)	NC_016702	Fresh water		
Sundasalanx praecox (1)	NC_016725	Fresh water		
Sundasalanx sp. Chao Phraya (1)	NC_016663			
Tenualosa ilisha (1)	NC_016682	Marine;Fresh water	?-200m	
Tenualosa toli (2)	NC_016700 AP011600			
Tenualosa thibaudeaui (1)	NC_016719	Fresh water		
Setipinna melanochir (1)	AP011565	Marine;Fresh water		
Clupeichthys goniognathus (1)	NC_016721	Fresh water		
Etrumeus micropus (1)	AP009139	Marine		
Microthrissa royauxi (2)	NC_016683 AP011596	Fresh water		
Pellonula vorax (1)	AP009231	Marine; Fresh water		
Potamothrissa obtusirostris (1)	NC_016698	Fresh water		
Pseudopentaceros richardsoni	NC_020016	Fresh water		

(1)

Osmeriformes (93)

Plecoglossus altivelis (7)	NC_002734<br>AB047553<br>EU124679-<br>EU124683	Marine;Fresh water	10-?m
Galaxias maculatus (2)	NC_004594<br>AP004104	Marine;Fresh water	
Galaxias gollumoides (2)	NC_015239<br>HM106487	Fresh water	
Galaxias sp. 'southern' (2)	NC_015240<br>HM106488		
Galaxiella nigrostriata (2)	NC_008448<br>AP006853	Fresh water	
Retropinna retropinna (2)	NC_004598<br>AP004108	Marine;Fresh water	
Hypomesus nipponensis (2)	NC_015242<br>HM106489	Fresh water	
Salangichthys microdon (2)	NC_004599<br>AP004109	Marine;Fresh water	
Salanx ariakensis (2)	NC_006918<br>AP006231	Marine;Fresh water	
Mallotus villosus (2)	NC_015244<br>HM106491	Marine;Fresh water	?-725m
Osmerus mordax (2)	NC_015246<br>HM106493	Marine;Fresh water	?-425m
**Alepocephalus tenebrosus (2)**	**NC_004590<br>AP004100**	**Marine**	**46-5500m**
**Alepocephalus australis (2)**	**NC_013566<br>AP009571**	**Marine**	**1000-2600m**
**Alepocephalus umbriceps (2)**	**NC_013572<br>AP009575**	**Marine**	**500-2000m**
**Alepocephalus agassizii (2)**	**NC_013564<br>AP009570**	**Marine**	**600-2500m**
**Alepocephalus bairdii (2)**	**NC_013567<br>AP009572**	**Marine**	**365-1700m**
**Alepocephalus bicolor (2)**	**NC_011012<br>AP009399**	**Marine**	**439-1080m**
**Alepocephalus productus (2)**	**NC_013570<br>AP009574**	**Marine**	**1150-3500m**
**Bathyprion danae (2)**	**NC_011015<br>AP009400**	**Marine**	**100-3200m**

Narcetes erimelas (2)	NC_011008 AP009405	Marine	1300-2600m	
Nansenia ardesiaca (2)	NC_004596 AP004106	Marine	260-553m	
Lipolagus ochotensis (2)	NC_004591 AP004101	Marine	?-6100m	
Opisthoproctus soleatus (2)	NC_004600 AP004110	Marine	300-800m	500-700m
Glossanodon semifasciatus (2)	NC_004595 AP004105	Marine	70-430m	
Sagamichthys abei (2)	NC_011010 AP009495	Marine	37-1500m	
Maulisia mauli (2)	NC_011007 AP009404	Marine	400-1200m	
Normichthys operosus (2)	NC_011009 AP009406	Marine	?-1000m	
Platytroctes apus (2)	NC_004597 AP004107	Marine	385-5393m	
Leptochilichthys agassizii (2)	NC_011006 AP009403	Marine	2000-3100m	
Bathytroctes macrolepis (2)	NC_013575 AP009577	Marine	2500-5850m	2800-3300m
Bathytroctes michaelsarsi (2)	NC_013579 AP009579	Marine	2010-5057m	
Bathytroctes breviceps (2)	NC_013574 AP009576	Marine	1650-1650m	
Bathytroctes microlepis (2)	NC_011017 AP009401	Marine	?-4900m	?-1800m
Conocara murrayi (2)	NC_013583 AP009581	Marine	1200-2603m	2000-2600m
Conocara macropterum (2)	NC_013581 AP009580	Marine	800-2200m	1200-1800m
Leptoderma lubricum (2)	NC_013557 AP009583	Marine	?-1700m	
Leptoderma retropinna (2)	NC_013559 AP009584	Marine	600-1100m	
Leptoderma macrophthalmum (2)	AP011500 NC_015100	Marine	2107-2063m	
Narcetes stomias (2)	NC_013560 AP009585	Marine	1500-2300m	1800-2100m
Talismania bifurcata (2)	NC_013562 AP009587	Marine	300-2000m	
Bajacalifornia megalops (2)	NC_013577 AP009578	Marine	150-3200m	800-1400m

**Herwigia kreffti (2)**	**NC_013555 AP009582**	**Marine**	**1000-3200m**	
**Alepocephalus longiceps (1)**	**AP009573**	**Marine**	**1240-1300m**	
**Bathylaco nigricans (1)**	**AP009494**	**Marine**	**450-4376m**	**1750-2200m**
**Rinoctes nasutus (1)**	**AP009586**	**Marine**	**2000-4156m**	**3650-?m**
**Xenodermichthys copei (1)**	**AP009588**	**Marine**	**100-2650m**	**100-1230m**
Gadiformes (73)				
Gadus morhua (33)	EU877710-EU877741 NC_002081	Marine	?-600m	150-200m
**Gadus chalcogrammus (11)**	**AB094061 NC_004449 AB182300-AB182308**	**Marine**	**?-1280m**	
Gadus ogac (2)	DQ356941 NC_012323	Marine	?-200m	
Melanogrammus aeglefinus (2)	NC_007396 DQ020497	Marine	10-450m	10-200m
Merlangius merlangus (2)	NC_007395 DQ020496	Marine	10-200m	30-100m
Boreogadus saida (2)	NC_010121 DQ356936	Marine	?-400m	
Physiculus japonicus (2)	NC_004377 AP004409	Marine	150-350m	
Lota lota (2)	NC_004379 AP004412	Fresh water	1-700m	
**Trachyrincus murrayi (2)**	**NC_008224 AP008990**	**Marine**	**?-1630m**	
**Bathygadus antrodes (2)**	**NC_008222 AP008988**	**Marine**	**792-1200m**	
Ventrifossa garmani (2)	NC_008225 AP008991	Marine	200-720m	350-550m
Coelorinchus kishinouyei (2)	NC_003169 AP002929	Marine	250-450m	
**Squalogadus modificatus (2)**	**NC_008223 AP008989**	**Marine**	**600-1740m**	
Bregmaceros nectabanus (2)	NC_008124 AP004411	Marine	?-350m	
**Micromesistius poutassou (1)**	**NC_015102**	**Marine**	**150-3000m**	**300-400m**
Pollachius pollachius (1)	NC_015097	Marine	?-200m	40-100

				m
Pollachius virens (1)	NC_015094	Marine	37-364m	
**Arctogadus glacialis (1)**	**NC_010122**	**Marine**	**?-1000m**	
**Merluccius merluccius (1)**	**NC_015120**	**Marine**	**30-1075 m**	**70-400 m**
**Siluriformes (72)**				
Silurus meridionalis (3)	HQ907992 HM746661 NC_014866	Fresh water		
Silurus asotus (2)	NC_015806 AP012022	Fresh water		
Silurus lanzhouensis (2)	NC_015650 JF895472	Fresh water		
Heteropneustes fossilis (2)	NC_015827 AP012013	Fresh water		
Pangasianodon gigas (2)	NC_006381 AY762971	Fresh water		
Pangasius larnaudii (2)	NC_015839 AP012018	Fresh water		
Liobagrus obesus (2)	NC_008232 DQ321752	Fresh water		
Pelteobagrus fulvidraco (2)	NC_015888 HM641815	Fresh water		
Pelteobagrus nitidus (3)	HM746659 NC_014859 KC822643	Fresh water		
Pelteobagrus vachellii (3)	HM746660 NC_014862 KT202282	Fresh water		
Pseudobagrus brevicorpus (2)	HM355585 NC_015625	Fresh water		
Pseudobagrus tokiensis (2)	NC_004697 AB054127	Fresh water		
Leiocassis longirostris (2)	NC_014586 GU596454	Fresh water		
Auchenoglanis occidentalis (2)	NC_015809 AP012005	Fresh water		
Cranoglanis bouderius (2)	NC_008280 AY898626	Fresh water		
Corydoras rabauti (2)	NC_004698 AB054128	Fresh water		
Pimelodus pictus (2)	NC_015797 AP012019	Fresh water		

Synodontis schoutedeni (2)	NC_015808<br>AP012023	Fresh water	
Diplomystes nahuelbutaensis (2)	NC_015823<br>AP012011	Fresh water	
Malapterurus electricus (2)	NC_015833<br>AP012016	Fresh water	
Amphilius sp. NM-2010 (2)	NC_015746<br>AP012002		
Bunocephalus coracoideus (2)	NC_015811<br>AP012006	Fresh water	
Clarias sp. NM-2010 (2)	NC_015749<br>AP012010		
Centromochlus perugiae (2)	NC_015748<br>AP012024	Fresh water	
Tetranematichthys quadrifilis (2)	NC_015743<br>AP012025	Fresh water	
Amblydoras gonzalezi (2)	NC_015745<br>AP012001	Fresh water	
Ictalurus punctatus (2)	NC_003489<br>AF482987	Fresh water	15-?m
Pareutropius debauwi (2)	NC_015837<br>AP012017	Fresh water	
Pterygoplichthys disjunctivus (2)	NC_015747<br>AP012021	Fresh water	
Silurus glanis (1)	NC_014261	Fresh water	?-30m
Hara jerdoni (1)	AP012012	Fresh water	
Trichomycterus areolatus (1)	AP012026	Fresh water	
Helogenes marmoratus (1)	AP012014	Fresh water	
Astroblepus sp. NM-2010 (1)	AP012004		
Chaca bankanensis (1)	AP012008	Fresh water	
Plotosus japonicus (1)	AP012020	Marine	
Cetopsidium sp. NM-2010 (1)	AP012007		
Chrysichthys sp. NM-2010 (1)	AP012009		
Liobagrus reinii (1)	AP012015	Fresh water	
Ariopsis seemanni (1)	AP012003	Marine; Fresh water	
**Osteoglossiformes (67)**			
Gnathonemus petersii (3)	AP009611<br>AP008928<br>NC_012717	Fresh water	
Brevimyrus niger (3)	AP009612<br>AP008929<br>NC_012705	Fresh water	

Gymnarchus niloticus (3)	AP009610<br>AP008930<br>NC_012707	Fresh water
Notopterus notopterus (3)	AP008925<br>AP008924<br>NC_012713	Fresh water
Xenomystus nigri (3)	AP009503<br>NC_012715<br>AP008927	Fresh water
Arapaima gigas (3)	AP009497<br>NC_010570<br>EF523611	Fresh water
Pantodon buchholzi (3)	NC_003096<br>AP011564<br>AB043068	Fresh water
Hiodon alosoides (2)	AP004356<br>NC_005145	Fresh water
Hiodon tergisus (2)	NC_015082<br>AP009499	Fresh water
Paramormyrops gabonensis (2)	NC_015107<br>AP009614	Fresh water
Petrocephalus soudanensis (2)	NC_015092<br>AP009502	Fresh water
Petrocephalus microphthalmus (2)	NC_015098<br>AP009609	Fresh water
Genyomyrus donnyi (2)	NC_015086<br>AP009500	Fresh water
Marcusenius senegalensis (2)	NC_015090<br>AP011575	Fresh water
Chitala ornata (2)	AP008923<br>NC_012712	Fresh water
Chitala blanci (2)	AP008921<br>NC_012710	Fresh water
Chitala lopis (2)	AP008922<br>NC_012711	Fresh water
Papyrocranus congoensis (2)	AP008926<br>NC_012714	Fresh water
Osteoglossum bicirrhosum (2)	NC_003095<br>AB043025	Fresh water

Scleropages formosus (2)	NC_007012 DQ023143	Fresh water	
Heterotis niloticus (2)	NC_015081 AP009498	Fresh water	
Pollimyrus adspersus (1)	AP011582	Fresh water	
Ivindomyrus marchei (1)	AP011574	Fresh water	
Mormyrops anguilloides (1)	AP011576	Fresh water	
Boulengeromyrus knoepffleri (1)	AP011568	Fresh water	
Hyperopisus bebe (1)	AP011572	Fresh water	
Paramormyrops sp. CU 6239 (1)	AP011578		
Paramormyrops sp. CU 6246 (1)	AP011580		
Paramormyrops sp. CU 6227 (1)	AP011579		
Paramormyrops sp. CU 6230 (1)	AP011581		
Brienomyrus brachyistius (1)	AP011569	Fresh water	
Campylomormyrus numenius (1)	AP011571	Fresh water	
Isichthys henryi (1)	AP011573	Fresh water	
Mormyrus rume (1)	AP011577	Fresh water	
Stomatorhinus cf. ater CU 6164 (1)	AP011583		
Cyphomyrus discorhynchus (2)	NC_015105 AP009613	Fresh water	
Nimbochromis linni (2)	NC_018558 JN628853	Fresh water	
**Lophiiformes (62)**			
Chaunax tosaensis (2)	NC004382 AP004416	Marine	
Chaunax abei (2)	NC004381 AP004415	Marine	90-500m
Chaunax pictus (2)	NC013883 AB282833	Marine	200-978m
Lophius americanus (2)	NC004380 AP004414	Marine	?-668m
Sladenia gardineri (2)	NC013873 AB282827		
Lophiomus setigerus (2)	NC008125 AP004413	Marine	30-800m

Lophiodes caulinaris (2)	NC013872 AB282826	Marine	15-311m	
**Melanocetus murrayi (2)**	**NC004384 AP004418**	**Marine**	**100-6370m**	**1000-2500m**
**Melanocetus johnsoni (2)**	**NC013866 AB282838**	**Marine**	**100-4500m**	**100-1500m**
**Neoceratias spinifer (2)**	**NC013864 AB282837**	**Marine**	**?-1200m**	
Himantolophus albinares (2)	NC013867 AB282839	Marine	845-?m	
Himantolophus groenlandicus (2)	NC013868 AB282840	Marine	830-?m	
**Bufoceratias thele (2)**	**NC013869 AB282841**	**Marine**	**?-1500m**	
**Diceratias pileatus (2)**	**NC013870 AB282842**	**Marine**	**640-1430m**	
**Oneirodes thompsoni (2)**	**NC013871 AB282843**	**Marine**	**100-2014m**	**400-600m**
**Thaumatichthys pagidostomus (2)**	**NC013875 AB282847**	**Marine**	**1392-1393m**	
**Ceratias uranoscopus (2)**	**NC013882 AB282851**	**Marine**	**?-2091m**	**500-1000m**
**Cryptopsaras couesii (2)**	**NC013880 AB282850**	**Marine**	**?-3085m**	**500-1250m**
**Rhynchactis macrothrix (2)**	**NC013863 AB282853**	**Marine**	**?-2000m**	
**Gigantactis vanhoeffeni (2)**	**NC013885 AB282852**	**Marine**	**300-5300m**	
Tetrabrachium ocellatum (2)	NC013879 AB282831	Marine	?-51m	
**Coelophrys brevicaudata (2)**	**NC013886 AB282834**	**Marine**	**1024-1024m**	
**Haplophryne mollis (2)**	**NC013865 AB282856**	**Marine**	**?-2250m**	
**Caulophryne pelagica (2)**	**NC016020 AB282836**	**Marine**	**?-2500m**	
Histrio histrio (1)	AB282829	Marine	?-50m	?-2m
Antennarius striatus (1)	AB282830	Marine	10-219m	?-40m
Antennatus coccineus (1)	AB282828	Marine	?-104m	?-30m
Zalieutes elater (1)	AB282835	Marine	18-113m	
Halieutaea stellata (1)	AP005977	Marine	50-400m	
Malthopsis jordani (1)	AP005978	Marine	185-520m	
**Chaenophryne melanorhabdus (1)**	**AB282845**	**Marine**	**?-1250m**	

Puck pinnata (1)	AB282844	Marine	
**Bertella idiomorpha (1)**	**AB282846**	**Marine**	**?-3475m**
Centrophryne spinulosus (1)	AB282849	Marine	
**Acentrophryne dolichonema (1)**	**AB282855**	**Marine**	**201-1105m**
Linophryne bicornis (1)	AB282854	Marine	
**Caulophryne jordani (1)**	**AP004417**	**Marine**	**100-1510m**
Lasiognathus sp. NMMBP 9030 (1)	AB282848		
**Beloniformes (41)**			
Oryzias latipes (15)	AB498065<br>AB498066<br>AP004421<br>AP008938-AP008948<br>NC_004387	Fresh water	
Oryzias javanicus (3)	AB498067<br>GU013790<br>NC_012981	Fresh water	
Oryzias dancena (3)	AB498069<br>GU013789<br>NC_012976	Fresh water	
Oryzias luzonensis (2)	AB498064<br>NC_012979	Fresh water	
Oryzias minutillus (2)	AB498068<br>NC_012975	Fresh water	
Oryzias sinensis (2)	GU013788<br>NC_013434	Fresh water	
Exocoetus volitans (2)	AP002933<br>NC_003184	Marine	?-20m
Cypselurus hiraii (2)	AB182653<br>NC_007403	Marine	
Hyporhamphus sajori (2)	AB370892<br>NC_011173	Marine;Fresh water	30-?m
Cololabis saira (2)	AP002932<br>NC_003183	Marine	?-230m
Ablennes hians (2)	AB373007<br>NC_011180	Marine	?-3m
Oryzias celebensis (1)	AB498070	Fresh water	
Oryzias marmoratus (1)	AP005981	Fresh water	
Oryzias sarasinorum (2)	NC_011172<br>AB370891	Fresh water	
**Characiformes (39)**			

Pygocentrus nattereri (2)	AP012000<br>NC_015840	Fresh water
Myleus sp. NM-2010 (2)	AP011997<br>NC_015751	
Hydrolycus scomberoides (2)	AP011989<br>NC_015813	Fresh water
Chalceus macrolepidotus (2)	AB054130<br>NC_004700	Fresh water
Phenacogrammus interruptus (2)	AB054129<br>NC_004699	Fresh water
Micralestes sp. NM-2010 (2)	AP011996<br>NC_015753	
Distichodus sexfasciatus (2)	AB070242<br>NC_015836	Fresh water
Chilodus punctatus (2)	AP011984<br>NC_015801	Fresh water
Hemiodopsis gracilis (2)	AP011990<br>NC_015816	Fresh water
Apareiodon affinis (2)	AP011998<br>NC_015834	Fresh water
Lebiasina astrigata (2)	AP011995<br>NC_015750	Fresh water
Ichthyborus sp. NM-2010 (2)	AP011993<br>NC_015752	
Citharinus congicus (2)	AP011985<br>NC_015805	Fresh water
Hepsetus odoe (2)	AP011991<br>NC_015819	Fresh water
Acestrorhynchus sp. NM-2010 (2)	AP011981<br>NC_015755	
Astyanax mexicanus (1)	AP011982	Fresh water
Paracheirodon innesi (1)	AP011999	Fresh water
Boulengerella maculata (1)	AB070207	Fresh water
Ctenolucius hujeta (1)	AP011987	Fresh water
Hoplias malabaricus (1)	AP011992	Fresh water
Crenuchus spilurus (1)	AP011986	Fresh water
Curimatopsis evelynae (1)	AP011988	Fresh water
Leporinus affinis (1)	AP011994	Fresh water
Carnegiella strigata (1)	AP011983	Fresh water
**Cyprinodontiformes (34)**		
Fundulus heteroclitus (7)	FJ445398-FJ445403<br>NC_012312	Marine;Fresh water

Xiphophorus hellerii (3)	FJ226476<br>FJ234985<br>NC_013089	Fresh water	
Fundulus grandis (3)	FJ445396<br>FJ445397<br>NC_012377	Fresh water	
Fundulus diaphanus (3)	FJ445394<br>FJ445395<br>NC_012361	Fresh water	
Xiphophorus maculatus (2)	AP005982<br>NC_011379	Fresh water	
Gambusia affinis (2)	AP004422<br>NC_004388	Fresh water	
Fundulus olivaceus (2)	AP006776<br>NC_011380	Fresh water	
Jordanella floridae (2)	AP006778<br>NC_011387	Fresh water	
Cyprinodon rubrofluviatilis (2)	EF442803<br>NC_009125	Fresh water	
Xenotoca eiseni (2)	AP006777<br>NC_011381	Fresh water	
Kryptolebias marmoratus (2)	NC_003290<br>AF283503	Fresh water	
Aplocheilus panchax (2)	AB373005<br>NC_011176	Fresh water	
Nothobranchius furzeri (2)	EU650204<br>NC_011814	Fresh water	
**Pleuronectiformes (22)**			
Paralichthys olivaceus (2)	AB028664<br>NC_002386	Marine	10-200m
Cynoglossus semilaevis (3)	EU366230<br>GQ380409<br>NC_012825	Marine;Fresh water	
Scophthalmus maximus (2)	EU419747<br>NC_013183	Marine	20-70m
Verasper moseri (2)	EF025506<br>NC_008461	Marine	1-900m
Verasper variegatus (2)	DQ403797<br>NC_007939	Marine	
Kareius bicoloratus (2)	AP002951<br>NC_003176	Marine;Fresh water	?-150m
Platichthys stellatus (2)	EF424428<br>NC_010966	Marine;Fresh water	?-375m

Solea senegalensis (2)	AB270760 NC_008327	Marine	12-65m	
Cynoglossus abbreviatus (2)	GQ380410 NC_014881	Marine	20-85m	
**Reinhardtius hippoglossoides (1)**	**NC_009711**	**Marine**	**1-2000m**	**500-1000m**
**Hippoglossus hippoglossus (1)**	**NC_009709**	**Marine**	**50-2000m**	
**Hippoglossus stenolepis (1)**	**NC_009710**	**Marine**	**?-1200m**	
**Stephanoberyciformes (29)**				
**Cetostoma regani (3)**	**AP004423 AP010882 NC_004389**	**Marine**	**?-2250m**	
**Barbourisia rufa (2)**	**AP010879 NC_012046**	**Marine**	**120-2000m**	
**Rondeletia loricata (2)**	**AP002937 NC_003186**	**Marine**	**100-3500m**	
**Gyrinomimus myersi (2)**	**AP010884 NC_012050**	**Marine**	**1280-2791m**	
Gyrinomimus sp. UW 113191 (2)	AP010883 NC_012049			
**Procetichthys kreffti (2)**	**AP010880 NC_012047**	**Marine**	**?-2200m**	
Cetomimus sp. AMS I34481001 (2)	AP010881 NC_012048			
**Danacetichthys galathenus (2)**	**AP002936 NC_003185**	**Marine**	**?-1330m**	
**Parataeniophorus gulosus (2)**	**AP010886 NC_012044**	**Marine**	**?-1400m**	
Eutaeniophorus sp. 033-Miya (2)	NC_004390 AP004424			
Eutaeniophorus festivus (2)	AP010885 NC_012043	Marine	?-200m	
**Ataxolepis apus (2)**	**AP010887 NC_012045**	**Marine**	**1464-1464m**	
**Scopelogadus mizolepis (2)**	**AP002934 NC_003171**	**Marine**	**300-3385m**	
**Poromitra oscitans (2)**	**AP002935 NC_003172**	**Marine**	**645-5320m**	
**Gasterosteiformes (24)**				
Aulostomus chinensis (2)	AP009197 NC_010269	Marine	3-122m	

Gasterosteus aculeatus (2)	AP002944 NC_003174	Marine;Fresh water	?-100m	
Gasterosteus wheatlandi (2)	AB445129 NC_011570	Marine		
Pungitius pungitius (2)	AB445130 NC_011571	Marine;Fresh water	?-110m	70-77m
Pungitius kaibarae (2)	EU332749 NC_014893	Marine;Fresh water		
Pungitius sinensis (2)	EU332748 NC_014889	Marine;Fresh water		
Apeltes quadracus (2)	AB445126 NC_011580	Marine;Fresh water	?-3m	
Spinachia spinachia (2)	AB445128 NC_011582	Marine		
Culaea inconstans (2)	AB445125 NC_011577	Fresh water	?-55m	
Aulichthys japonicus (2)	AB445127 NC_011569	Marine		
Aulorhynchus flavidus (2)	AP009196 NC_010268	Marine	?-30m	
Hypoptychus dybowskii (2)	AP004437 NC_004400	Marine		
**Beryciformes (25)**				
**Beryx splendens (3)**	**AP002939 DQ996312 NC_003188**	**Marine**	**25-1300 m**	**400-600m**
**Diretmus argenteus (2)**	**AP004427 NC_008127**	**Marine**	**?-2000m**	**500-700m**
**Diretmoides veriginae (2)**	**AP004426 NC_008126**	**Marine**	**340-1300m**	
**Anoplogaster cornuta (2)**	**AP004425 NC_004391**	**Marine**	**2-4992m**	**500-2000**
Hoplostethus japonicus (2)	AP002938 NC_003187	Marine	336-605m	
Monocentris japonicus (2)	AP004429 NC_004392	Marine	10-200m	
Anomalops katoptron (2)	AP004428 NC_008128	Marine	2-400m	
**Beryx decadactylus (2)**	**AP004430 NC_004393**	**Marine**	**110-1000m**	**200-400m**
Beryx mollis (2)	DQ993168 NC_013845	Marine	100-500m	

Ostichthys japonicus (2)	AP004431<br>NC_004394	Marine		
Myripristis berndti (2)	AP002940<br>NC_003189	Marine	1-50m	3-15m
Sargocentron rubrum (2)	AP004432<br>NC_004395	Marine	1-84m	
**Polypteriformes (22)**				
Erpetoichthys calabaricus (4)	AP004350<br>AY442348<br>HM143925<br>NC_005251	Fresh water		
Polypterus ornatipinnis (2)	AP004351<br>NC_001778	Fresh water		
Polypterus endlicherii (1)	HM143931	Fresh water		
Polypterus senegalus senegalus (2)	AP004352<br>NC_004418	Fresh water		
Polypterus retropinnis (1)	HM143937	Fresh water		
Polypterus endlicherii congicus (1)	HM143929	Fresh water	?-10m	
Polypterus bichir bichir (1)	HM143927	Fresh water		
Polypterus bichir lapradei (1)	HM143928	Fresh water		
Polypterus sp. Koliba (1)	HM143933			
Polypterus palmas buettikoferi (1)	HM143935	Fresh water		
Polypterus palmas polli (1)	HM143936	Fresh water		
Polypterus weeksii (1)	HM143939	Fresh water		
Polypterus delhezi (1)	HM143930	Fresh water		
Polypterus ansorgii (1)	HM143926	Fresh water		
Polypterus sp. golden dust (1)	HM143932			
Polypterus mokelembembe (1)	HM143934	Fresh water		
Polypterus teugelsi (1)	HM143938	Fresh water		
**Acipenseriformes (20)**				
Polyodon spathula (4)	AP004353<br>AY442349<br>AY510086<br>NC_004419	Fresh water	2-?m	
Acipenser transmontanus (2)	AB042837<br>NC_004743	Marine;Fresh water	1-122m	
Acipenser sinensis (2)	EU719645<br>NC_012646	Marine;Fresh water		
Acipenser gueldenstaedtii (2)	FJ392605<br>NC_012576	Marine;Fresh water	1-?m	

Acipenser dabryanus (2)	AY510085<br>NC_005451	Fresh water		
Scaphirhynchus cf. albus (2)	AP004354<br>AP004354			
Huso huso (2)	AY442351<br>NC_005252	Marine;Fresh water	70-180m	
Psephurus gladius (2)	AY571339<br>NC_005834	Fresh water		
Acipenser baerii (1)	JQ045341	Marine;Fresh water	1-?m	
Acipenser stellatus (1)	NC_005795	Marine;Fresh water	10-100m	
**Gonorynchiformes (18)**				
Cromeria nilotica (1)	AP011560	Fresh water		
Phractolaemus ansorgii (3)	AB070243<br>AP007280<br>NC_007892	Fresh water		
Chanos chanos (2)	AB054133<br>NC_004693	Marine;Fresh water	1-30m	
Grasseichthys gabonensis (2)	AP007277<br>NC_007890	Fresh water		
Parakneria cameronensis (2)	AP007279<br>NC_007891	Fresh water		
Kneria sp. SL-2004 (2)	AP007278<br>NC_007882			
Gonorynchus abbreviatus (2)	AP009402<br>NC_011018	Marine	50-100m	
Gonorynchus greyi (2)	AB054134<br>NC_004702	Marine	?-160m	
Cromeria occidentalis (2)	NC_007881<br>AP007275	Fresh water		
**Syngnathiformes (18)**				
Aeoliscus strigatus (2)	AP009198<br>NC_010270	Marine	1-20m	
Macroramphosus scolopax (2)	AP005988<br>NC_010265	Marine	25-600m	50-350 m
Pegasus volitans (2)	AP005984<br>NC_010271	Marine	1-73m	9-27m
Eurypegasus draconis (2)	AP005983<br>NC_010264	Marine	3-91m	35-90m
Microphis brachyurus (2)	AP005986<br>NC_010273	Marine;Fresh water		

Hippocampus kuda (2)	AP005985<br>NC_010272	Marine	?-68m	
Indostomus paradoxus (2)	AP004438<br>NC_004401	Fresh water		
**Solenostomus cyanopterus (2)**	**AB277725**<br>**NC_010267**	**Marine**	**?-25m**	**?-25m**
Fistularia commersonii (2)	AP005987<br>NC_010274	Marine	?-132m	
**Scorpaeniformes (18)**				
Scalicus amiscus (2)	AP004441<br>NC_004403			
**Aptocyclus ventricosus (2)**	AP004443<br>NC_008129	**Marine**	**?-1700m**	
Clinocottus analis (2)	FJ848374<br>NC_013828	Marine	?-18m	
Cottus poecilopus (2)	EU332750<br>NC_014849	Fresh water	?-15m	
Cottus reinii (2)	AP004442<br>NC_004404	Fresh water		
Cottus hangiongensis (2)	EU332751<br>NC_014851	Marine;Fresh water		
Sebastes schlegelii (2)	NC_005450<br>AY491978	Marine	3-100m	
Helicolenus hilgendorfi (2)	AP002948<br>NC_003195	Marine	150-500m	
Sebastiscus marmoratus (2)	GU452728<br>NC_013812	Marine		
**Zeiformes (12)**				
Parazen pacificus (2)	AP004433<br>NC_004396	Marine	145-500m	
**Allocyttus niger (2)**	**AP004435**<br>**NC_004398**	**Marine**	**560-1300m**	
**Neocyttus rhomboidalis (2)**	**AP004436**<br>**NC_004399**	**Marine**	**200-1240m**	**450-800m**
Zenopsis nebulosus (2)	AP002942<br>NC_003173	Marine	30-800m	50-600m
Zeus faber (2)	AP002941<br>NC_003190	Marine	5-400m	50-150m
Zenion japonicum (2)	AP004434<br>NC_004397	Marine	200-400m	
**Ophidiiformes (12)**				

**Bassozetus zenkevitchi (2)**	**AP004405 NC_004374**	**Marine**	**?-6930m**	
**Lamprogrammus niger (2)**	**AP004410 NC_004378**	**Marine**	**741-2000m**	**?-1500m**
Sirembo imberbis (2)	AP004406 NC_008123	Marine	100-200m	
Carapus bermudensis (2)	AP004404 NC_004373	Marine	1-235m	
**Cataetyx rubrirostris (2)**	**AP004407 NC_004375**	**Marine**	**300-1000m**	
**Diplacanthopoma brachysoma (2)**	**AP004408 NC_004376**	**Marine**	**460-1670m**	
**Gymnotiformes (10)**				
Gymnorhamphichthys sp. NM-2010 (2)	AP011980 NC_015754			
Eigenmannia sp. CBM-ZF-10620 (2)	AB054131 NC_004701			
Apteronotus albifrons (2)	AB054132 NC_004692	Fresh water		
Electrophorus electricus (1)	AP011978	Fresh water		
Gymnotus carapo (1)	AP011979	Fresh water		
Brachyhypopomus occidentalis (2)	AP011570 NC_015078	Fresh water		
**Atheriniformes (10)**				
Melanotaenia lacustris (2)	NC_004385 AP004419	Fresh water		
Odontesthes sp. Odsp-001 (2)	NC_011175 AB370894			
Menidia menidia (2)	NC_011174 AB370893	Marine	?-3m	
Hypoatherina tsurugae (2)	NC_004386 AP004420	Marine		
Iso hawaiiensis (2)	NC_011178 AB373006	Marine	?-2m	
**Aulopiformes (10)**				
Harpadon microchir (2)	NC_003161 AP002919	Marine		
Saurida undosquamis (2)	NC_003162 AP002920	Marine	1-350m	
Synodus variegatus (2)	NC_007228 AY524977	Marine	3-121m	5-60m

Aulopus japonicus (2)	NC_002674 AB047821	Marine	85-510m	
Chlorophthalmus agassizi (2)	NC_003160 AP002918	Marine	50-1000m	
**Lampriformes (9)**				
Stylephorus chordatus (3)	NC_009948 AB280687 AB280688	Marine		
Lampris guttatus (2)	NC_003165 AP002924	Marine	100-500 m	?-366m
Zu cristatus (2)	NC_003167 AP002926	Marine	?-90m	
Trachipterus trachypterus (2)	NC_003166 AP002925	Marine	100-600m	
**Elopiformes (8)**				
Elops hawaiensis (2)	AB051070 NC_005798	Marine;Fresh water	1-30m	
Elops saurus (2)	AP004807 NC_005803	Marine	?-50m	
Megalops atlanticus (2)	NC_005804 AP004808	Marine;Fresh water	?-30m	?-15m
Megalops cyprinoides (2)	AB051110 NC_005799	Marine;Fresh water	50-?m	
**Semionotiformes (7)**				
Lepisosteus oculatus (3)	NC_004744 AB042861 AY442350	Fresh water		
Lepisosteus osseus (2)	DQ536423 NC_008104	Fresh water		
Atractosteus spatula (2)	NC_008131 AP004355	Fresh water		
**Myctophiformes (6)**				
**Diaphus splendidus (2)**	**NC_003164 AP002923**	**Marine**	**?-8000m**	
Myctophum affine (2)	NC_003163 AP002922	Marine	?-600m	
Neoscopelus microchir (2)	NC_003180 AP002921	Marine	250-700m	
**Stomiiformes (5)**				
**Chauliodus sloani (2)**	**NC_003159**	**Marine**	**200-470**	**494-10**

	**AP002915**		**0m**	**00m**
**Sigmops gracilis (2)**	**NC_002574 AB016274**	**Marine**	**?-4389m**	**?-800m**
**Diplophos taenia (1)**	**NC_002647**	**Marine**	**?-1594m**	**450-61 0m**
**Synbranchiformes (5)**				
Monopterus albus (2)	NC_003192 AP002945	Fresh water	3-?m	
Mastacembelus favus (2)	NC_003193 AP002946	Fresh water		
Synbranchus marmoratus (1)	AP004439	Fresh water		
**Esociformes (4)**				
Dallia pectoralis (2)	NC_004592 AP004102	Fresh water		
Esox lucius (2)	NC_004593 AP004103	Fresh water	?-30m	1-5m
**Dactylopteriformes (4)**				
Dactyloptena peterseni (2)	NC_003194 AP002947	Marine	50-210m	
Dactyloptena tiltoni (2)	NC_004402 AP004440	Marine	119-565m	
**Percopsiformes (4)**				
Aphredoderus sayanus (2)	NC_004372 AP004403	Fresh water		
Percopsis transmontana (2)	NC_003168 AP002928	Fresh water		
**Polymixiiformes (4)**				
Polymixia japonica (2)	NC_002648 AB034826	Marine	160-628m	
Polymixia lowei (2)	NC_003181 AP002927	Marine	50-600m	150-60 0m
**Ateleopodiformes (4)**				
**Ijimaia dofleini (2)**	**NC_003179 AP002917**	**Marine**	**?-1281m**	
Ateleopus japonicus (2)	NC_003178 AP002916	Marine	140-600m	

**Notacanthiformes (4)**				
**Notacanthus chemnitzii (2)**	**AP002975 NC_005144**	**Marine**	**125-3285m**	**125-1000m**
**Aldrovandia affinis (2)**	**NC_005801 AP002974**	**Marine**	**730-2560m**	
**Elassomatidae (4)**				
Elassoma zonatum (2)	NC_011388 AP006813	Fresh water		
Elassoma evergladei (2)	NC_003175 AP002950	Fresh water		
**Amiiformes (3)**				
Amia calva (3)	AB042952 AY442347 NC_004742	Fresh water		
**Mugiliformes (3)**				
Mugil cephalus (3)	NC_003182 AP002931 AP002930	Marine	?-120m	?-10m
**Batrachoidiformes (3)**				
Porichthys myriaster (2)	NC_006920 AP006739			
Batrachomoeus trispinosus (1)	AP006738	Marine		
**Albuliformes (3)**				
Albula glossodonta (2)	NC_005800 AP002973	Marine		
**Pterothrissus gissu (1)**	**NC_005796**	**Marine**	**?-1000m**	
**Carangimorpha (12)**				
Trachurus japonicus (3)	NC_002813 AP003092 AP003091	Marine	?-275m	50-275m
**Trachurus trachurus (2)**	**NC_006818 AB108498**	**Marine**	**?-1050m**	**100-200m**
Carangoides armatus (2)	NC_004405 AP004444	Marine		

Caranx melampygus (2)	NC_004406<br>AP004445	Marine	?-190m	
Seriola quinqueradiata (1)	NC_016868	Marine	100-?m	
Seriola lalandi (1)	NC_016869	Marine	3-825m	
Seriola dumerili (1)	NC_016870	Marine	1-360m	18-72m
**Scombriformes (11)**				
Scomber japonicus (2)	NC_013723<br>AB488405	Marine	?-300m	50-200 m
Scomber australasicus (2)	NC_013725<br>AB488407	Marine	87-200m	
Scomber colias (2)	NC_013724<br>AB488406	Marine		
**Scomber scombrus (1)**	**NC_006398**	**Marine**	**?-1000m**	**?-200m**
Rastrelliger brachysoma (2)	NC_013485<br>EU555283	Marine	15-200m	
Rastrelliger kanagurta (2)	NC_019624<br>JX524134	Marine	20-90m	

Supplementary Table 2: Selective pressure analyses for the 13 genes in Anguilliformes.

1. ATP6

Model	Parameter Estimated[a]	p	lnL	2⊿lnL[b]<br>*P* value[c]	Positively Selected Sites (BEB Analysis)[d]
**One ratio**	ω =0.0500	118	-15891.25		
**Branch-site Model**					
Branch a	site class 0 1 2a 2b<br>proportion 0.93146 0.06854 0.00000 0.00000<br>background w 0.04116 1.00000 0.04116 1.00000<br>foreground w 0.04116 1.00000 1.00000 1.00000	121	-15710.48	0<br>*P*=1	
Branch a<br>ω_a = 1	site class 0 1 2a 2b<br>proportion 0.93146 0.06854 0.00000 0.00000<br>background w 0.04116 1.00000 0.04116 1.00000<br>foreground w 0.04116 1.00000 1.00000 1.00000	120	-15710.48		
Branch b	site class 0 1 2a 2b<br>proportion 0.93146 0.06854 0.00000 0.00000<br>background w 0.04116 1.00000 0.04116 1.00000<br>foreground w 0.04116 1.00000 1.00000 1.00000	121	-15710.48	0<br>*P*=1	
Branch b<br>ω_b = 1	site class 0 1 2a 2b<br>proportion 0.93146 0.06854 0.00000 0.00000<br>background w 0.04116 1.00000 0.04116 1.00000<br>foreground w 0.04116 1.00000 1.00000 1.00000	120	-15710.48		
Branch c	site class 0 1 2a 2b<br>proportion 0.79528 0.05865 0.13604 0.01003<br>background w 0.04030 1.00000 0.04030 1.00000<br>foreground w 0.04030 1.00000 1.00000 1.00000	121	-15706.18	0<br>*P*=1	
Branch c<br>ω_c = 1	site class 0 1 2a 2b<br>proportion 0.79528 0.05865 0.13604 0.01003<br>background w 0.04030 1.00000 0.04030 1.00000<br>foreground w 0.04030 1.00000 1.00000 1.00000	120	-15706.18		
Branch d	site class 0 1 2a 2b<br>proportion 0.93146 0.06854 0.00000 0.00000<br>background w 0.04116 1.00000 0.04116 1.00000<br>foreground w 0.04116 1.00000 1.00000 1.00000	121	-15710.48	0<br>*P*=1	
Branch d<br>ω_d = 1	site class 0 1 2a 2b<br>proportion 0.93146 0.06854 0.00000 0.00000<br>background w 0.04116 1.00000 0.04116 1.00000<br>foreground w 0.04116 1.00000 1.00000 1.00000	120	-15710.48		

2. ATP8

Model	Parameter Estimated[a]	p	lnL	2⊿lnL[b]<br>*P* value[c]	Positively Selected Sites (BEB Analysis)[d]

**One ratio**	ω =0.1171	118	-3895.55		
**Branch-site Model**					
Branch a	site class 0 1 2a 2b<br>proportion 0.85251 0.12912 0.01596 0.00242<br>background w 0.10413 1.00000 0.10413 1.00000<br>foreground w 0.10413 1.00000 1.00000 1.00000	121	-3868.33	0<br>*P*=1	
Branch a<br>ω_a = 1	site class 0 1 2a 2b<br>proportion 0.85251 0.12912 0.01596 0.00242<br>background w 0.10413 1.00000 0.10413 1.00000<br>foreground w 0.10413 1.00000 1.00000 1.00000	120	-3868.33		
Branch b	site class 0 1 2a 2b<br>proportion 0.86862 0.13138 0.00000 0.00000<br>background w 0.10432 1.00000 0.10432 1.00000<br>foreground w 0.10432 1.00000 1.00000 1.00000	121	-3868.34	0<br>*P*=1	
Branch b<br>ω_b = 1	site class 0 1 2a 2b<br>proportion 0.86862 0.13138 0.00000 0.00000<br>background w 0.10432 1.00000 0.10432 1.00000<br>foreground w 0.10432 1.00000 1.00000 1.00000	120	-3868.34		
Branch c	site class 0 1 2a 2b<br>proportion 0.67102 0.10151 0.19758 0.02989<br>background w 0.10280 1.00000 0.10280 1.00000<br>foreground w 0.10280 1.00000 1.00000 1.00000	121	-3868.13	0<br>*P*=1	
Branch c<br>ω_c = 1	site class 0 1 2a 2b<br>proportion 0.67102 0.10151 0.19758 0.02989<br>background w 0.10280 1.00000 0.10280 1.00000<br>foreground w 0.10280 1.00000 1.00000 1.00000	120	-3868.13		
Branch d	site class 0 1 2a 2b<br>proportion 0.86862 0.13138 0.00000 0.00000<br>background w 0.10432 1.00000 0.10432 1.00000<br>foreground w 0.10432 1.00000 1.00000 1.00000	121	-3868.34	0<br>*P*=1	
Branch d<br>ω_d = 1	site class 0 1 2a 2b<br>proportion 0.86862 0.13138 0.00000 0.00000<br>background w 0.10432 1.00000 0.10432 1.00000<br>foreground w 0.10432 1.00000 1.00000 1.00000	120	-3868.34		

3. CoxI

Model	Parameter Estimated[a]	p	lnL	2⊿lnL[b]<br>*P* value[c]	Positively Selected Sites (BEB Analysis)[d]
**One ratio**	ω =0.0187	118	-26843.53		
**Branch-site Model**					
Branch a	site class 0 1 2a 2b<br>proportion 0.98849 0.01151 0.00000 0.00000<br>background w 0.01634 1.00000 0.01634 1.00000<br>foreground w 0.01634 1.00000 1.00000 1.00000	121	-26725.73	0<br>*P*=1	

Branch a<br>ω_b = 1	site class 0 1 2a 2b<br>proportion 0.98849 0.01151 0.00000 0.00000<br>background w 0.01634 1.00000 0.01634 1.00000<br>foreground w 0.01634 1.00000 1.00000 1.00000	120	-26725.73		
Branch b	site class 0 1 2a 2b<br>proportion 0.96527 0.01129 0.02317 0.00027<br>background w 0.01622 1.00000 0.01622 1.00000<br>foreground w 0.01622 1.00000 1.00000 1.00000	121	-26724.98	0<br>*P*=1	
Branch b<br>ω_b = 1	site class 0 1 2a 2b<br>proportion 0.96526 0.01129 0.02317 0.00027<br>background w 0.01622 1.00000 0.01622 1.00000<br>foreground w 0.01622 1.00000 1.00000 1.00000	120	-26724.98		
Branch c	site class 0 1 2a 2b<br>proportion 0.97934 0.01141 0.00915 0.00011<br>background w 0.01630 1.00000 0.01630 1.00000<br>foreground w 0.01630 1.00000 1.00000 1.00000	121	-26725.64	0<br>*P*=1	
Branch c<br>ω_b = 1	site class 0 1 2a 2b<br>proportion 0.97934 0.01141 0.00915 0.00011<br>background w 0.01630 1.00000 0.01630 1.00000<br>foreground w 0.01630 1.00000 1.00000 1.00000	120	-26725.64		
Branch d	site class 0 1 2a 2b<br>proportion 0.98849 0.01151 0.00000 0.00000<br>background w 0.01634 1.00000 0.01634 1.00000<br>foreground w 0.01634 1.00000 1.00000 1.00000	121	-26725.73	0<br>*P*=1	
Branch d<br>ω_b = 1	site class 0 1 2a 2b<br>proportion 0.98849 0.01151 0.00000 0.00000<br>background w 0.01634 1.00000 0.01634 1.00000<br>foreground w 0.01634 1.00000 1.00000 1.00000	120	-26725.73		

4. CoxII

Model	Parameter Estimated[a]	p	lnL	2⊿lnL[b]<br>*P* value[c]	Positively Selected Sites (BEB Analysis)[d]
**One ratio**	ω =0.0301	118	-11727.45		
**Branch-site Model**					
Branch a	site class 0 1 2a 2b<br>proportion 0.96603 0.01759 0.01608 0.00029<br>background w 0.02772 1.00000 0.02772 1.00000<br>foreground w 0.02772 1.00000 3.36860 3.36860	121	-11700.41	0.7<br>*P*=0.40	
Branch a<br>ω_b = 1	site class 0 1 2a 2b<br>proportion 0.94605 0.01723 0.03607 0.00066<br>background w 0.02770 1.00000 0.02770 1.00000<br>foreground w 0.02770 1.00000 1.00000 1.00000	120	-11700.76		
Branch b	site class 0 1 2a 2b	121	-11699.22	0	

	proportion 0.79755 0.01465 0.18441 0.00339<br>background w 0.02739 1.00000 0.02739 1.00000<br>foreground w 0.02739 1.00000 1.00000 1.00000			*P*=1	
Branch b<br>ω_b = 1	site class 0 1 2a 2b<br>proportion 0.79755 0.01465 0.18441 0.00339<br>background w 0.02739 1.00000 0.02739 1.00000<br>foreground w 0.02739 1.00000 1.00000 1.00000	120	-11699.22		
Branch c	site class 0 1 2a 2b<br>proportion 0.95608 0.01738 0.02607 0.00047<br>background w 0.02794 1.00000 0.02794 1.00000<br>foreground w 0.02794 1.00000 1.00000 1.00000	121	-11703.24	0<br>*P*=1	
Branch c<br>ω_b = 1	site class 0 1 2a 2b<br>proportion 0.95608 0.01738 0.02607 0.00047<br>background w 0.02794 1.00000 0.02794 1.00000<br>foreground w 0.02794 1.00000 1.00000 1.00000	120	-11703.24		
Branch d	site class 0 1 2a 2b<br>proportion 0.98218 0.01782 0.00000 0.00000<br>background w 0.02809 1.00000 0.02809 1.00000<br>foreground w 0.02809 1.00000 1.00000 1.00000	121	-11703.53	0<br>*P*=1	
Branch d<br>ω_b = 1	site class 0 1 2a 2b<br>proportion 0.98218 0.01782 0.00000 0.00000<br>background w 0.02809 1.00000 0.02809 1.00000<br>foreground w 0.02809 1.00000 1.00000 1.00000	120	-11703.53		

5. CoxIII

Model	Parameter Estimated[a]	p	lnL	2⊿lnL[b]<br>*P* value[c]	Positively Selected Sites (BEB Analysis)[d]
**One ratio**	ω =0.0258	118	-13881.43		
**Branch-site Model**					
Branch a	site class 0 1 2a 2b<br>proportion 0.94925 0.05075 0.00000 0.00000<br>background w 0.01818 1.00000 0.01818 1.00000<br>foreground w 0.01818 1.00000 1.00000 1.00000	121	-13765.88	0<br>*P*=1	
Branch a<br>ω_b = 1	site class 0 1 2a 2b<br>proportion 0.94925 0.05075 0.00000 0.00000<br>background w 0.01818 1.00000 0.01818 1.00000<br>foreground w 0.01818 1.00000 1.00000 1.00000	120	-13765.88		
Branch b	site class 0 1 2a 2b<br>proportion 0.94925 0.05075 0.00000 0.00000<br>background w 0.01818 1.00000 0.01818 1.00000<br>foreground w 0.01818 1.00000 1.00000 1.00000	121	-13765.88	0<br>*P*=1	
Branch b<br>ω_b = 1	site class 0 1 2a 2b<br>proportion 0.94925 0.05075 0.00000 0.00000<br>background w 0.01818 1.00000 0.01818 1.00000	120	-13765.88		

	foreground w 0.01818 1.00000 1.00000 1.00000				
Branch c	site class 0 1 2a 2b<br>proportion 0.94925 0.05075 0.00000 0.00000<br>background w 0.01818 1.00000 0.01818 1.00000<br>foreground w 0.01818 1.00000 1.00000 1.00000	121	-13765.88	0<br>*P*=1	
Branch c<br>ω_b = 1	site class 0 1 2a 2b<br>proportion 0.94925 0.05075 0.00000 0.00000<br>background w 0.01818 1.00000 0.01818 1.00000<br>foreground w 0.01818 1.00000 1.00000 1.00000	120	-13765.88		
Branch d	site class 0 1 2a 2b<br>proportion 0.94925 0.05075 0.00000 0.00000<br>background w 0.01818 1.00000 0.01818 1.00000<br>foreground w 0.01818 1.00000 1.00000 1.00000	121	-13765.88	0<br>*P*=1	
Branch d<br>ω_b = 1	site class 0 1 2a 2b<br>proportion 0.94925 0.05075 0.00000 0.00000<br>background w 0.01818 1.00000 0.01818 1.00000<br>foreground w 0.01818 1.00000 1.00000 1.00000	120	-13765.88		

6. CytB

**Model**	**Parameter Estimated**[a]	**p**	**lnL**	**2⊿lnL**[b]<br>***P* value**[c]	**Positively Selected Sites (BEB Analysis)**[d]
**One ratio**	ω =0.0301	118	-24182.00		
**Branch-site Model**					
Branch a	site class 0 1 2a 2b<br>proportion 0.97300 0.02700 0.00000 0.00000<br>background w 0.02760 1.00000 0.02760 1.00000<br>foreground w 0.02760 1.00000 1.00000 1.00000	121	-24101.70	0<br>*P*=1	
Branch a<br>ω_b = 1	site class 0 1 2a 2b<br>proportion 0.97300 0.02700 0.00000 0.00000<br>background w 0.02760 1.00000 0.02760 1.00000<br>foreground w 0.02760 1.00000 1.00000 1.00000	120	-24101.70		
Branch b	site class 0 1 2a 2b<br>proportion 0.96557 0.02660 0.00762 0.00021<br>background w 0.02748 1.00000 0.02748 1.00000<br>foreground w 0.02748 1.00000 10.74283 10.74283	121	-24099.69	1.02<br>*P*=0.31	
Branch b<br>ω_b = 1	site class 0 1 2a 2b<br>proportion 0.94580 0.02610 0.02735 0.00075<br>background w 0.02749 1.00000 0.02749 1.00000<br>foreground w 0.02749 1.00000 1.00000 1.00000	120	-24100.72		
Branch c	site class 0 1 2a 2b<br>proportion 0.80934 0.02286 0.16319 0.00461<br>background w 0.02703 1.00000 0.02703 1.00000<br>foreground w 0.02703 1.00000 4.15272 4.15272	121	-24091.51	0.52<br>*P*=0.47	74 N 0.999<br>153 V 0.831

Branch c<br>ω_b = 1	site class 0 1 2a 2b<br>proportion 0.68021 0.01926 0.29226 0.00828<br>background w 0.02706 1.00000 0.02706 1.00000<br>foreground w 0.02706 1.00000 1.00000 1.00000	120	-24091.77		
Branch d	site class 0 1 2a 2b<br>proportion 0.97300 0.02700 0.00000 0.00000<br>background w 0.02760 1.00000 0.02760 1.00000<br>foreground w 0.02760 1.00000 1.00000 1.00000	121	-24101.70	0<br>*P*=1	
Branch d<br>ω_b = 1	site class 0 1 2a 2b<br>proportion 0.97300 0.02700 0.00000 0.00000<br>background w 0.02760 1.00000 0.02760 1.00000<br>foreground w 0.02760 1.00000 1.00000 1.00000	120	-24101.70		

7. ND1

**Model**	**Parameter Estimated[a]**	**p**	**lnL**	**2⊿lnL[b]**<br>***P* value[c]**	**Positively Selected Sites (BEB Analysis)[d]**
**One ratio**	ω =0.0353	118	-20913.06		
**Branch-site Model**					
Branch a	site class 0 1 2a 2b<br>proportion 0.96543 0.03457 0.00001 0.00000<br>background w 0.03102 1.00000 0.03102 1.00000<br>foreground w 0.03102 1.00000 1.00000 1.00000	121	-20778.10	0<br>*P*=1	
Branch a<br>ω_b = 1	site class 0 1 2a 2b<br>proportion 0.96543 0.03457 0.00000 0.00000<br>background w 0.03102 1.00000 0.03102 1.00000<br>foreground w 0.03102 1.00000 1.00000 1.00000	120	-20778.10		
Branch b	site class 0 1 2a 2b<br>proportion 0.95662 0.03438 0.00869 0.00031<br>background w 0.03092 1.00000 0.03092 1.00000<br>foreground w 0.03092 1.00000 4.84030 4.84030	121	-20777.20	0.84<br>*P*=0.36	
Branch b<br>ω_b = 1	site class 0 1 2a 2b<br>proportion 0.94289 0.03387 0.02243 0.00081<br>background w 0.03093 1.00000 0.03093 1.00000<br>foreground w 0.03093 1.00000 1.00000 1.00000	120	-20777.62		
**Branch c**	site class 0 1 2a 2b<br>proportion 0.95770 0.03003 0.01190 0.00037<br>background w 0.03125 1.00000 0.03125 1.00000<br>foreground w 0.03125 1.00000 999.00000 999.00000	121	-20770.74	**13.06**<br>***P*<0.001**	246 I 0.996
Branch c<br>ω_b = 1	site class 0 1 2a 2b<br>proportion 0.92914 0.03005 0.03952 0.00128<br>background w 0.03121 1.00000 0.03121 1.00000<br>foreground w 0.03121 1.00000 1.00000 1.00000	120	-20777.27		
Branch d	site class 0 1 2a 2b	121	-20778.10	0	

	proportion 0.96543 0.03457 0.00001 0.00000<br>background w 0.03102 1.00000 0.03102 1.00000<br>foreground w 0.03102 1.00000 1.00000 1.00000			*P*=1	
Branch d<br>$\omega_b = 1$	site class 0 1 2a 2b<br>proportion 0.96543 0.03457 0.00000 0.00000<br>background w 0.03102 1.00000 0.03102 1.00000<br>foreground w 0.03102 1.00000 1.00000 1.00000	120	-20778.10		

8. ND2

**Model**	**Parameter Estimated**[a]	**p**	**lnL**	**2⊿lnL**[b]<br>***P* value**[c]	**Positively Selected Sites (BEB Analysis)**[d]
**One ratio**	ω =0.0740	118	-27711.69		
**Branch-site Model**					
Branch a	site class 0 1 2a 2b<br>proportion 0.92981 0.07019 0.00000 0.00000<br>background w 0.06727 1.00000 0.06727 1.00000<br>foreground w 0.06727 1.00000 1.00000 1.00000	121	-27527.26	0<br>*P*=1	
Branch a<br>$\omega_b = 1$	site class 0 1 2a 2b<br>proportion 0.92982 0.07018 0.00000 0.00000<br>background w 0.06727 1.00000 0.06727 1.00000<br>foreground w 0.06727 1.00000 1.00000 1.00000	120	-27527.26		
Branch b	site class 0 1 2a 2b<br>proportion 0.90472 0.06819 0.02519 0.00190<br>background w 0.06714 1.00000 0.06714 1.00000<br>foreground w 0.06714 1.00000 1.00000 1.00000	121	-27527.15	0<br>*P*=1	
Branch b<br>$\omega_b = 1$	site class 0 1 2a 2b<br>proportion 0.90472 0.06819 0.02519 0.00190<br>background w 0.06714 1.00000 0.06714 1.00000<br>foreground w 0.06714 1.00000 1.00000 1.00000	120	-27527.15		
Branch c	site class 0 1 2a 2b<br>proportion 0.91963 0.06920 0.01039 0.00078<br>background w 0.06712 1.00000 0.06712 1.00000<br>foreground w 0.06712 1.00000 998.99949 998.99949	121	-27522.57	**8.76**<br>***P*=0.003**	204 N 0.995
Branch c<br>$\omega_b = 1$	site class 0 1 2a 2b<br>proportion 0.90785 0.06839 0.02210 0.00166<br>background w 0.06712 1.00000 0.06712 1.00000<br>foreground w 0.06712 1.00000 1.00000 1.00000	120	-27526.95		
Branch d	site class 0 1 2a 2b<br>proportion 0.82782 0.06255 0.10193 0.00770<br>background w 0.06689 1.00000 0.06689 1.00000<br>foreground w 0.06689 1.00000 1.00000 1.00000	121	-27526.22	0<br>*P*=1	
Branch d<br>$\omega_b = 1$	site class 0 1 2a 2b<br>proportion 0.82782 0.06255 0.10193 0.00770<br>background w 0.06689 1.00000 0.06689 1.00000	120	-27526.22		

	foreground w 0.06689 1.00000 1.00000 1.00000				

9. ND3

Model	Parameter Estimated[a]	p	lnL	2⊿lnL[b] *P* value[c]	Positively Selected Sites (BEB Analysis)[d]
**One ratio**	ω =0.0518	118	-8179.46		
**Branch-site Model**					
Branch a	site class 0 1 2a 2b<br>proportion 0.89313 0.10687 0.00000 0.00000<br>background w 0.04267 1.00000 0.04267 1.00000<br>foreground w 0.04267 1.00000 1.00000 1.00000	121	-8113.62	0<br>*P*=1	
Branch a ω_b = 1	site class 0 1 2a 2b<br>proportion 0.89313 0.10687 0.00000 0.00000<br>background w 0.04267 1.00000 0.04267 1.00000<br>foreground w 0.04267 1.00000 1.00000 1.00000	120	-8113.62		
Branch b	site class 0 1 2a 2b<br>proportion 0.89313 0.10687 0.00000 0.00000<br>background w 0.04267 1.00000 0.04267 1.00000<br>foreground w 0.04267 1.00000 1.00000 1.00000	121	-8113.62	0<br>*P*=1	
Branch b ω_b = 1	site class 0 1 2a 2b<br>proportion 0.89313 0.10687 0.00000 0.00000<br>background w 0.04267 1.00000 0.04267 1.00000<br>foreground w 0.04267 1.00000 1.00000 1.00000	120	-8113.62		
Branch c	site class 0 1 2a 2b<br>proportion 0.89313 0.10687 0.00000 0.00000<br>background w 0.04267 1.00000 0.04267 1.00000<br>foreground w 0.04267 1.00000 1.00000 1.00000	121	-8113.62	0.1<br>*P*=0.75	
Branch c ω_b = 1	site class 0 1 2a 2b<br>proportion 0.76892 0.09186 0.12437 0.01486<br>background w 0.04255 1.00000 0.04255 1.00000<br>foreground w 0.04255 1.00000 1.00000 1.00000	120	-8113.67		
Branch d	site class 0 1 2a 2b<br>proportion 0.89313 0.10687 0.00000 0.00000<br>background w 0.04267 1.00000 0.04267 1.00000<br>foreground w 0.04267 1.00000 1.00000 1.00000	121	-8113.62	0<br>*P*=1	
Branch d ω_b = 1	site class 0 1 2a 2b<br>proportion 0.89313 0.10687 0.00000 0.00000<br>background w 0.04267 1.00000 0.04267 1.00000<br>foreground w 0.04267 1.00000 1.00000 1.00000	120	-8113.62		

10. ND4

Model	Parameter Estimated[a]	p	lnL	2⊿lnL[b] *P* value[c]	Positively Selected Sites (BEB Analysis)[d]

**One ratio**	ω =0.0430	118	-31480.13		
**Branch-site Model**					
Branch a	site class 0 1 2a 2b<br>proportion 0.94541 0.05459 0.00000 0.00000<br>background w 0.03445 1.00000 0.03445 1.00000<br>foreground w 0.03445 1.00000 1.00000 1.00000	121	-31070.92	0<br>*P*=1	
Branch a<br>ω_b = 1	site class 0 1 2a 2b<br>proportion 0.94541 0.05459 0.00000 0.00000<br>background w 0.03445 1.00000 0.03445 1.00000<br>foreground w 0.03445 1.00000 1.00000 1.00000	120	-31070.92		
Branch b	site class 0 1 2a 2b<br>proportion 0.94541 0.05459 0.00000 0.00000<br>background w 0.03445 1.00000 0.03445 1.00000<br>foreground w 0.03445 1.00000 1.00000 1.00000	121	-31070.92	0<br>*P*=1	
Branch b<br>ω_b = 1	site class 0 1 2a 2b<br>proportion 0.94541 0.05459 0.00000 0.00000<br>background w 0.03445 1.00000 0.03445 1.00000<br>foreground w 0.03445 1.00000 1.00000 1.00000	120	-31070.92		
Branch c	site class 0 1 2a 2b<br>proportion 0.94541 0.05459 0.00000 0.00000<br>background w 0.03445 1.00000 0.03445 1.00000<br>foreground w 0.03445 1.00000 1.00000 1.00000	121	-31070.92	0<br>*P*=1	
Branch c<br>ω_b = 1	site class 0 1 2a 2b<br>proportion 0.94541 0.05459 0.00000 0.00000<br>background w 0.03445 1.00000 0.03445 1.00000<br>foreground w 0.03445 1.00000 1.00000 1.00000	120	-31070.92		
Branch d	site class 0 1 2a 2b<br>proportion 0.93738 0.05408 0.00807 0.00047<br>background w 0.03441 1.00000 0.03441 1.00000<br>foreground w 0.03441 1.00000 1.00000 1.00000	121	-31070.87	0<br>*P*=1	
Branch d<br>ω_b = 1	site class 0 1 2a 2b<br>proportion 0.93738 0.05408 0.00807 0.00047<br>background w 0.03441 1.00000 0.03441 1.00000<br>foreground w 0.03441 1.00000 1.00000 1.00000	120	-31070.87		

11. ND4L

Model	Parameter Estimated[a]	p	lnL	2ΔlnL[b]<br>*P* value[c]	Positively Selected Sites (BEB Analysis)[d]
**One ratio**	ω =0.0635	118	-7150.00		
**Branch-site Model**					
Branch a	site class 0 1 2a 2b<br>proportion 0.95183 0.04817 0.00000 0.00000<br>background w 0.05793 1.00000 0.05793 1.00000<br>foreground w 0.05793 1.00000 1.00000 1.00000	121	-7112.97	0<br>*P*=1	

Branch a<br>$\omega_b = 1$	site class 0 1 2a 2b<br>proportion 0.95183 0.04817 0.00000 0.00000<br>background w 0.05793 1.00000 0.05793 1.00000<br>foreground w 0.05793 1.00000 1.00000 1.00000	120	-7112.97		
Branch b	site class 0 1 2a 2b<br>proportion 0.95183 0.04817 0.00000 0.00000<br>background w 0.05793 1.00000 0.05793 1.00000<br>foreground w 0.05793 1.00000 1.00000 1.00000	121	-7112.97	0<br>*P*=1	
Branch b<br>$\omega_b = 1$	site class 0 1 2a 2b<br>proportion 0.95183 0.04817 0.00000 0.00000<br>background w 0.05793 1.00000 0.05793 1.00000<br>foreground w 0.05793 1.00000 1.00000 1.00000	120	-7112.97		
Branch c	site class 0 1 2a 2b<br>proportion 0.95183 0.04817 0.00000 0.00000<br>background w 0.05793 1.00000 0.05793 1.00000<br>foreground w 0.05793 1.00000 1.00000 1.00000	121	-7112.97	0<br>*P*=1	
Branch c<br>$\omega_b = 1$	site class 0 1 2a 2b<br>proportion 0.95183 0.04817 0.00000 0.00000<br>background w 0.05793 1.00000 0.05793 1.00000<br>foreground w 0.05793 1.00000 1.00000 1.00000	120	-7112.97		
Branch d	site class 0 1 2a 2b<br>proportion 0.95183 0.04817 0.00000 0.00000<br>background w 0.05794 1.00000 0.05794 1.00000<br>foreground w 0.05794 1.00000 1.00000 1.00000	121	-7112.97	0<br>*P*=1	
Branch d<br>$\omega_b = 1$	site class 0 1 2a 2b<br>proportion 0.95183 0.04817 0.00000 0.00000<br>background w 0.05793 1.00000 0.05793 1.00000<br>foreground w 0.05793 1.00000 1.00000 1.00000	120	-7112.97		

12. ND5

**Model**	**Parameter Estimated**[a]	**p**	**lnL**	**2⊿lnL**[b]<br>***P* value**[c]	**Positively Selected Sites (BEB Analysis)**[d]
**One ratio**	ω =0.0559	118	-41913.41		
**Branch-site Model**					
Branch a	site class 0 1 2a 2b<br>proportion 0.91818 0.08182 0.00000 0.00000<br>background w 0.04420 1.00000 0.04420 1.00000<br>foreground w 0.04420 1.00000 1.00000 1.00000	121	-41324.81	0<br>*P*=1	
Branch a<br>$\omega_b = 1$	site class 0 1 2a 2b<br>proportion 0.91818 0.08182 0.00000 0.00000<br>background w 0.04420 1.00000 0.04420 1.00000<br>foreground w 0.04420 1.00000 1.00000 1.00000	120	-41324.81		
Branch b	site class 0 1 2a 2b	121	-41323.95	0.32	

	proportion 0.91063 0.07926 0.00930 0.00081<br>background w 0.04411 1.00000 0.04411 1.00000<br>foreground w 0.04411 1.00000 6.63014 6.63014			*P*=0.57	
Branch b<br>ω_b = 1	site class 0 1 2a 2b<br>proportion 0.88887 0.07798 0.03047 0.00267<br>background w 0.04409 1.00000 0.04409 1.00000<br>foreground w 0.04409 1.00000 1.00000 1.00000	120	-41324.11		
Branch c	site class 0 1 2a 2b<br>proportion 0.91559 0.08161 0.00257 0.00023<br>background w 0.04411 1.00000 0.04411 1.00000<br>foreground w 0.04411 1.00000 9.99723 9.99723	121	-41323.58	1.62<br>*P*=0.20	
Branch c<br>ω_b = 1	site class 0 1 2a 2b<br>proportion 0.91239 0.08130 0.00579 0.00052<br>background w 0.04414 1.00000 0.04414 1.00000<br>foreground w 0.04414 1.00000 1.00000 1.00000	120	-41324.39		
Branch d	site class 0 1 2a 2b<br>proportion 0.91818 0.08182 0.00000 0.00000<br>background w 0.04420 1.00000 0.04420 1.00000<br>foreground w 0.04420 1.00000 1.00000 1.00000	121	-41324.81	0<br>*P*=1	
Branch d<br>ω_b = 1	site class 0 1 2a 2b<br>proportion 0.91818 0.08182 0.00000 0.00000<br>background w 0.04420 1.00000 0.04420 1.00000<br>foreground w 0.04420 1.00000 1.00000 1.00000	120	-41324.81		

13. ND6

**Model**	**Parameter Estimated**[a]	**p**	**lnL**	**2⊿lnL**[b]<br>***P* value**[c]	**Positively Selected Sites (BEB Analysis)**[d]
**One ratio**	ω =0.0489	118	-12966.71		
**Branch-site Model**					
Branch a	site class 0 1 2a 2b<br>proportion 0.92747 0.07253 0.00000 0.00000<br>background w 0.04719 1.00000 0.04719 1.00000<br>foreground w 0.04719 1.00000 1.00000 1.00000	121	-12861.41	0<br>*P*=1	
Branch a<br>ω_b = 1	site class 0 1 2a 2b<br>proportion 0.92747 0.07253 0.00000 0.00000<br>background w 0.04719 1.00000 0.04719 1.00000<br>foreground w 0.04719 1.00000 1.00000 1.00000	120	-12861.41		
Branch b	site class 0 1 2a 2b<br>proportion 0.92747 0.07253 0.00000 0.00000<br>background w 0.04719 1.00000 0.04719 1.00000<br>foreground w 0.04719 1.00000 1.00000 1.00000	121	-12861.41	0<br>*P*=1	
Branch b<br>ω_b = 1	site class 0 1 2a 2b<br>proportion 0.92747 0.07253 0.00000 0.00000<br>background w 0.04719 1.00000 0.04719 1.00000	120	-12861.41		

	foreground w 0.04719 1.00000 1.00000 1.00000				
Branch c	site class 0 1 2a 2b<br>proportion 0.92747 0.07253 0.00000 0.00000<br>background w 0.04719 1.00000 0.04719 1.00000<br>foreground w 0.04719 1.00000 1.00000 1.00000	121	-12861.41	0<br>*P*=1	
Branch c<br>$\omega_b = 1$	site class 0 1 2a 2b<br>proportion 0.92747 0.07253 0.00000 0.00000<br>background w 0.04719 1.00000 0.04719 1.00000<br>foreground w 0.04719 1.00000 1.00000 1.00000	120	-12861.41		
Branch d	site class 0 1 2a 2b<br>proportion 0.00000 0.00000 0.92746 0.07254<br>background w 0.04690 1.00000 0.04690 1.00000<br>foreground w 0.04690 1.00000 1.00000 1.00000	121	-12860.63	0<br>*P*=1	
Branch d<br>$\omega_b = 1$	site class 0 1 2a 2b<br>proportion 0.00001 0.00000 0.92745 0.07254<br>background w 0.04690 1.00000 0.04690 1.00000<br>foreground w 0.04690 1.00000 1.00000 1.00000	120	-12860.63		

[a] The proportion of sites estimated to have ω

[b] Twice the difference in log-likelihood values was calculated following a chi-squared ($\chi 2$)

[c] Posterior probability>0.99 of having ω>1

[d] The residues were identified by Bayes Empiriacal Bayes analysis

Supplementary Table 3: Selective pressure analyses for the 13 genes in Beryciformes and Stephanoberyciformes.

1. ATP6

**Model**	**Parameter Estimated**[a]	**p**	**lnL**	**2⊿lnL**[b] ***P* value**[c]	**Positively Selected Sites (BEB Analysis)**[d]
**One ratio**	ω =0.0599	53	-7930.15		
**Branch-site Model**					
Branch e	site class 0 1 2a 2b proportion 0.84989 0.04079 0.10431 0.00501 background w 0.05047 1.00000 0.05047 1.00000 foreground w 0.05047 1.00000 1.05095 1.05095	56	-7869.03	0 *P*=1	
Branch e ω_a =1	site class 0 1 2a 2b proportion 0.87195 0.04185 0.08226 0.00395 background w 0.05047 1.00000 0.05047 1.00000 foreground w 0.05047 1.00000 1.00000 1.00000	55	-7869.03		
Branch f	site class 0 1 2a 2b proportion 0.76300 0.03965 0.18760 0.00975 background w 0.04643 1.00000 0.04643 1.00000 foreground w 0.04643 1.00000 1.75640 1.75640	56	-7851.36	1 *P*=0.32	
Branch f ω_b =1	site class 0 1 2a 2b proportion 0.72481 0.03770 0.22575 0.01174 background w 0.04633 1.00000 0.04633 1.00000 foreground w 0.04633 1.00000 1.00000 1.00000	55	-7851.86		
Branch g	site class 0 1 2a 2b proportion 0.93986 0.04526 0.01420 0.00068 background w 0.04945 1.00000 0.04945 1.00000 foreground w 0.04945 1.00000 80.57827 80.57827	56	-7862.45	**5.8** ***P*=0.016**	179 S 0.992
Branch g ω_c =1	site class 0 1 2a 2b proportion 0.90604 0.04357 0.04807 0.00231 background w 0.04929 1.00000 0.04929 1.00000 foreground w 0.04929 1.00000 1.00000 1.00000	55	-7865.35		

2. ATP8

**Model**	**Parameter Estimated**[a]	**p**	**lnL**	**2⊿lnL**[b] ***P* value**[c]	**Positively Selected Sites (BEB Analysis)**[d]
**One ratio**	ω =0.1521	53	-1984.43		
**Branch-site Model**					
Branch e	site class 0 1 2a 2b proportion 0.71969 0.28031 0.00000 0.00000 background w 0.09810 1.00000 0.09810 1.00000 foreground w 0.09810 1.00000 1.00000 1.00000	56	-1965.04	0 *P*=1	
Branch e	site class 0 1 2a 2b	55	-1965.04		

ω_a =1	proportion 0.71968 0.28032 0.00000 0.00000<br>background w 0.09810 1.00000 0.09810 1.00000<br>foreground w 0.09810 1.00000 1.00000 1.00000				
Branch f	site class 0 1 2a 2b<br>proportion 0.73885 0.19312 0.05393 0.01410<br>background w 0.10957 1.00000 0.10957 1.00000<br>foreground w 0.10957 1.00000 39.68379 39.68379	56	-1962.04	**4.72**<br>***P*=0.030**	49 E 0.974
Branch f<br>ω_b =1	site class 0 1 2a 2b<br>proportion 0.66777 0.22320 0.08172 0.02731<br>background w 0.10026 1.00000 0.10026 1.00000<br>foreground w 0.10026 1.00000 1.00000 1.00000	55	-1964.40		
Branch g	site class 0 1 2a 2b<br>proportion 0.71969 0.28031 0.00000 0.00000<br>background w 0.09810 1.00000 0.09810 1.00000<br>foreground w 0.09810 1.00000 1.00000 1.00000	56	-1965.04	0<br>*P*=1	
Branch g<br>ω_c =1	site class 0 1 2a 2b<br>proportion 0.71969 0.28031 0.00000 0.00000<br>background w 0.09810 1.00000 0.09810 1.00000<br>foreground w 0.09810 1.00000 1.00000 1.00000	55	-1965.04		

3. CoxI

Model	Parameter Estimated[a]	p	lnL	2⊿lnL[b]<br>*P* value[c]	Positively Selected Sites (BEB Analysis)[d]
**One ratio**	ω =0.0269	53	-13890.08		
**Branch-site Model**					
Branch e	site class 0 1 2a 2b<br>proportion 0.96504 0.03160 0.00325 0.00011<br>background w 0.01965 1.00000 0.01965 1.00000<br>foreground w 0.01965 1.00000 1.00000 1.00000	56	-13813.67	0<br>*P*=1	
Branch e<br>ω_a =1	site class 0 1 2a 2b<br>proportion 0.96505 0.03160 0.00325 0.00011<br>background w 0.01965 1.00000 0.01965 1.00000<br>foreground w 0.01965 1.00000 1.00000 1.00000	55	-13813.67		
Branch f	site class 0 1 2a 2b<br>proportion 0.90842 0.03291 0.05662 0.00205<br>background w 0.01722 1.00000 0.01722 1.00000<br>foreground w 0.01722 1.00000 1.13624 1.13624	56	-13796.27	0.02<br>*P*=0.89	
Branch f<br>ω_b =1	site class 0 1 2a 2b<br>proportion 0.90406 0.03274 0.06099 0.00221<br>background w 0.01720 1.00000 0.01720 1.00000<br>foreground w 0.01720 1.00000 1.00000 1.00000	55	-13796.28		
Branch g	site class 0 1 2a 2b<br>proportion 0.96832 0.03168 0.00000 0.00000	56	-13813.68	0<br>*P*=1	

	background w 0.01969 1.00000 0.01969 1.00000<br>foreground w 0.01969 1.00000 1.00000 1.00000				
Branch g<br>ω_c =1	site class 0 1 2a 2b<br>proportion 0.96832 0.03168 0.00000 0.00000<br>background w 0.01969 1.00000 0.01969 1.00000<br>foreground w 0.01969 1.00000 1.00000 1.00000	55	-13813.68		

4. CoxII

Model	Parameter Estimated[a]	p	lnL	2⊿lnL[b]<br>*P* value[c]	Positively Selected Sites (BEB Analysis)[d]
**One ratio**	ω =0.0561	53	-6391.13		
**Branch-site Model**					
Branch e	site class 0 1 2a 2b<br>proportion 0.93697 0.03000 0.03201 0.00102<br>background w 0.04782 1.00000 0.04782 1.00000<br>foreground w 0.04782 1.00000 1.00000 1.00000	56	-6350.96	0<br>*P*=1	
Branch e<br>ω_a =1	site class 0 1 2a 2b<br>proportion 0.93697 0.03000 0.03201 0.00102<br>background w 0.04782 1.00000 0.04782 1.00000<br>foreground w 0.04782 1.00000 1.00000 1.00000	55	-6350.96		
Branch f	site class 0 1 2a 2b<br>proportion 0.67086 0.02265 0.29649 0.01001<br>background w 0.03798 1.00000 0.03798 1.00000<br>foreground w 0.03798 1.00000 2.19846 2.19846	56	-6300.37	**4.24**<br>***P*=0.039**	54 Y 0.997<br>56 L 0.997<br>57 D 0.996<br>61 I 0.991<br>68 L 0.994<br>107 S 0.998<br>126 Q 0.996<br>130 S 0.998<br>187 S 0.998<br>215 P 0.998<br>216 L 0.994<br>223 S 0.996<br>224 S 0.998<br>229 D 0.998
Branch f<br>ω_b =1	site class 0 1 2a 2b<br>proportion 0.60024 0.02037 0.36694 0.01245<br>background w 0.03767 1.00000 0.03767 1.00000<br>foreground w 0.03767 1.00000 1.00000 1.00000	55	-6302.49		
Branch g	site class 0 1 2a 2b<br>proportion 0.96878 0.03122 0.00000 0.00000<br>background w 0.04812 1.00000 0.04812 1.00000<br>foreground w 0.04812 1.00000 1.00000 1.00000	56	-6351.08	0<br>*P*=1	
Branch g<br>ω_c =1	site class 0 1 2a 2b<br>proportion 0.96878 0.03122 0.00000 0.00000<br>background w 0.04812 1.00000 0.04812 1.00000<br>foreground w 0.04812 1.00000 1.00000 1.00000	55	-6351.08		

5. CoxIII

Model	Parameter Estimated[a]	p	lnL	2⊿lnL[b]	Positively

				***P* value**[c]	**Selected Sites**<br>**(BEB Analysis)**[d]
**One ratio**	ω =0.0336	53	-7082.38		
**Branch-site Model**					
Branch e	site class 0 1 2a 2b<br>proportion 0.95899 0.04101 0.00000 0.00000<br>background w 0.02453 1.00000 0.02453 1.00000<br>foreground w 0.02453 1.00000 1.00000 1.00000	56	-7030.47	0<br>*P*=1	
Branch e<br>ω_a =1	site class 0 1 2a 2b<br>proportion 0.95899 0.04101 0.00000 0.00000<br>background w 0.02453 1.00000 0.02453 1.00000<br>foreground w 0.02453 1.00000 1.00000 1.00000	55	-7030.47		
Branch f	site class 0 1 2a 2b<br>proportion 0.92020 0.04110 0.03705 0.00165<br>background w 0.02247 1.00000 0.02247 1.00000<br>foreground w 0.02247 1.00000 17.01597 17.01597	56	-7015.97	**4.76**<br>***P*=0.029**	39 S 0.997<br>52 L 0.987
Branch f<br>ω_b =1	site class 0 1 2a 2b<br>proportion 0.85766 0.03834 0.09956 0.00445<br>background w 0.02225 1.00000 0.02225 1.00000<br>foreground w 0.02225 1.00000 1.00000 1.00000	55	-7018.35		
Branch g	site class 0 1 2a 2b<br>proportion 0.95408 0.03888 0.00676 0.00028<br>background w 0.02433 1.00000 0.02433 1.00000<br>foreground w 0.02433 1.00000 6.81078 6.81078	56	-7028.08	1.84<br>*P*=0.17	213 T 0.968
Branch g<br>ω_c =1	site class 0 1 2a 2b<br>proportion 0.94716 0.03867 0.01361 0.00056<br>background w 0.02434 1.00000 0.02434 1.00000<br>foreground w 0.02434 1.00000 1.00000 1.00000	55	-7029.00		

6. CytB

**Model**	**Parameter Estimated**[a]	**p**	**lnL**	**2⊿lnL**[b]<br>***P* value**[c]	**Positively Selected Sites**<br>**(BEB Analysis)**[d]
**One ratio**	ω =0.0357	53	-11764.12		
**Branch-site Model**					
Branch e	site class 0 1 2a 2b<br>proportion 0.97072 0.02928 0.00000 0.00000<br>background w 0.03096 1.00000 0.03096 1.00000<br>foreground w 0.03096 1.00000 1.00000 1.00000	56	-11692.68	0<br>*P*=1	
Branch e<br>ω_a =1	site class 0 1 2a 2b<br>proportion 0.97072 0.02928 0.00000 0.00000<br>background w 0.03096 1.00000 0.03096 1.00000<br>foreground w 0.03096 1.00000 1.00000 1.00000	55	-11692.68		
Branch f	site class 0 1 2a 2b<br>proportion 0.94755 0.02841 0.02334 0.00070	56	-11689.25	0.14<br>*P*=0.71	

	background w 0.03044 1.00000 0.03044 1.00000<br>foreground w 0.03044 1.00000 1.45204 1.45204				
Branch f<br>ω_b =1	site class 0 1 2a 2b<br>proportion 0.94089 0.02825 0.02996 0.00090<br>background w 0.03039 1.00000 0.03039 1.00000<br>foreground w 0.03039 1.00000 1.00000 1.00000	55	-11689.32		
Branch g	site class 0 1 2a 2b<br>proportion 0.92902 0.02748 0.04225 0.00125<br>background w 0.03013 1.00000 0.03013 1.00000<br>foreground w 0.03013 1.00000 1.00000 1.00000	56	-11689.48	0<br>*P*=1	
Branch g<br>ω_c =1	site class 0 1 2a 2b<br>proportion 0.92902 0.02748 0.04225 0.00125<br>background w 0.03013 1.00000 0.03013 1.00000<br>foreground w 0.03013 1.00000 1.00000 1.00000	55	-11689.48		

7. ND1

**Model**	**Parameter Estimated**[a]	**p**	**lnL**	**2⊿lnL**[b]<br>***P* value**[c]	**Positively Selected Sites (BEB Analysis)**[d]
**One ratio**	ω =0.0341	53	-10596.35		
**Branch-site Model**					
Branch e	site class 0 1 2a 2b<br>proportion 0.81170 0.01899 0.16544 0.00387<br>background w 0.03082 1.00000 0.03082 1.00000<br>foreground w 0.03082 1.00000 2.54310 2.54310	56	-10571.89	1.92<br>*P*=0.17	
Branch e<br>ω_a =1	site class 0 1 2a 2b<br>proportion 0.97712 0.02288 0.00000 0.00000<br>background w 0.03089 1.00000 0.03089 1.00000<br>foreground w 0.03089 1.00000 1.00000 1.00000	55	-10570.93		
Branch f	site class 0 1 2a 2b<br>proportion 0.87543 0.01980 0.10245 0.00232<br>background w 0.02969 1.00000 0.02969 1.00000<br>foreground w 0.02969 1.00000 1.00000 1.00000	56	-10567.87	0<br>*P*=1	
Branch f<br>ω_b =1	site class 0 1 2a 2b<br>proportion 0.87543 0.01980 0.10245 0.00232<br>background w 0.02969 1.00000 0.02969 1.00000<br>foreground w 0.02969 1.00000 1.00000 1.00000	55	-10567.87		
Branch g	site class 0 1 2a 2b<br>proportion 0.97712 0.02288 0.00000 0.00000<br>background w 0.03088 1.00000 0.03088 1.00000<br>foreground w 0.03088 1.00000 1.00000 1.00000	56	-10570.93	0<br>*P*=1	
Branch g<br>ω_c =1	site class 0 1 2a 2b<br>proportion 0.97712 0.02288 0.00000 0.00000<br>background w 0.03088 1.00000 0.03088 1.00000<br>foreground w 0.03088 1.00000 1.00000 1.00000	55	-10570.93		

8. ND2

Model	Parameter Estimated[a]	p	lnL	2⊿lnL[b]<br>*P* value[c]	Positively Selected Sites (BEB Analysis)[d]
**One ratio**	ω =0.0693	53	-12904.82		
**Branch-site Model**					
Branch e	site class 0 1 2a 2b<br>proportion 0.89358 0.05717 0.04629 0.00296<br>background w 0.05821 1.00000 0.05821 1.00000<br>foreground w 0.05821 1.00000 1.00000 1.00000	56	-12806.40	0.24<br>*P*=0.62	
Branch e<br>ω_a =1	site class 0 1 2a 2b<br>proportion 0.93991 0.06009 0.00000 0.00000<br>background w 0.05845 1.00000 0.05845 1.00000<br>foreground w 0.05845 1.00000 1.00000 1.00000	55	-12806.52		
Branch f	site class 0 1 2a 2b<br>proportion 0.80716 0.05142 0.13295 0.00847<br>background w 0.05630 1.00000 0.05630 1.00000<br>foreground w 0.05630 1.00000 1.00000 1.00000	56	-12799.02	0<br>*P*=1	
Branch f<br>ω_b =1	site class 0 1 2a 2b<br>proportion 0.80716 0.05143 0.13295 0.00847<br>background w 0.05630 1.00000 0.05630 1.00000<br>foreground w 0.05630 1.00000 1.00000 1.00000	55	-12799.02		
Branch g	site class 0 1 2a 2b<br>proportion 0.93991 0.06009 0.00000 0.00000<br>background w 0.05845 1.00000 0.05845 1.00000<br>foreground w 0.05845 1.00000 1.00000 1.00000	56	-12806.52	0.4<br>*P*=0.53	
Branch g<br>ω_c =1	site class 0 1 2a 2b<br>proportion 0.93062 0.05970 0.00910 0.00058<br>background w 0.05817 1.00000 0.05817 1.00000<br>foreground w 0.05817 1.00000 1.00000 1.00000	55	-12806.32		

9. ND3

Model	Parameter Estimated[a]	p	lnL	2⊿lnL[b]<br>*P* value[c]	Positively Selected Sites (BEB Analysis)[d]
**One ratio**	ω =0.0658	53	-4191.01		
**Branch-site Model**					
Branch e	site class 0 1 2a 2b<br>proportion 0.72472 0.11473 0.13861 0.02194<br>background w 0.03215 1.00000 0.03215 1.00000<br>foreground w 0.03215 1.00000 1.00000 1.00000	56	-4087.90	1.36<br>*P*=0.24	
Branch e<br>ω_a =1	site class 0 1 2a 2b<br>proportion 0.86275 0.13725 0.00000 0.00000<br>background w 0.03236 1.00000 0.03236 1.00000<br>foreground w 0.03236 1.00000 1.00000 1.00000	55	-4087.22		

Branch f	site class 0 1 2a 2b<br>proportion 0.75756 0.12533 0.10049 0.01662<br>background w 0.02852 1.00000 0.02852 1.00000<br>foreground w 0.02852 1.00000 1.00000 1.00000	56	-4083.08	0<br>*P*=1	
Branch f<br>ω_b =1	site class 0 1 2a 2b<br>proportion 0.75756 0.12533 0.10049 0.01662<br>background w 0.02852 1.00000 0.02852 1.00000<br>foreground w 0.02852 1.00000 1.00000 1.00000	55	-4083.08		
Branch g	site class 0 1 2a 2b<br>proportion 0.86275 0.13725 0.00000 0.00000<br>background w 0.03236 1.00000 0.03236 1.00000<br>foreground w 0.03236 1.00000 1.00000 1.00000	56	-4087.22	0<br>*P*=1	
Branch g<br>ω_c =1	site class 0 1 2a 2b<br>proportion 0.86275 0.13725 0.00000 0.00000<br>background w 0.03236 1.00000 0.03236 1.00000<br>foreground w 0.03236 1.00000 1.00000 1.00000	55	-4087.22		

10. ND4

Model	Parameter Estimated[a]	p	lnL	2⊿lnL[b]<br>*P* value[c]	Positively Selected Sites (BEB Analysis)[d]
**One ratio**	ω =0.0618	53	-16106.77		
**Branch-site Model**					
Branch e	site class 0 1 2a 2b<br>proportion 0.91295 0.04574 0.03934 0.00197<br>background w 0.05039 1.00000 0.05039 1.00000<br>foreground w 0.05039 1.00000 1.00000 1.00000	56	-15961.08	0<br>*P*=1	
Branch e<br>ω_a =1	site class 0 1 2a 2b<br>proportion 0.91308 0.04575 0.03920 0.00196<br>background w 0.05039 1.00000 0.05039 1.00000<br>foreground w 0.05039 1.00000 1.00000 1.00000	55	-15961.08		
Branch f	site class 0 1 2a 2b<br>proportion 0.91431 0.04677 0.03703 0.00189<br>background w 0.04967 1.00000 0.04967 1.00000<br>foreground w 0.04967 1.00000 1.35491 1.35491	56	-15957.68	0.08<br>*P*=0.78	
Branch f<br>ω_b =1	site class 0 1 2a 2b<br>proportion 0.90344 0.04623 0.04788 0.00245<br>background w 0.04961 1.00000 0.04961 1.00000<br>foreground w 0.04961 1.00000 1.00000 1.00000	55	-15957.72		
Branch g	site class 0 1 2a 2b<br>proportion 0.95189 0.04811 0.00000 0.00000<br>background w 0.05065 1.00000 0.05065 1.00000<br>foreground w 0.05065 1.00000 1.00000 1.00000	56	-15961.36	0<br>*P*=1	
Branch g	site class 0 1 2a 2b	55	-15961.36		

ω_c =1	proportion 0.95180 0.04810 0.00010 0.00000<br>background w 0.05065 1.00000 0.05065 1.00000<br>foreground w 0.05065 1.00000 1.00000 1.00000				

11. ND4L

Model	Parameter Estimated[a]	p	lnL	2⊿lnL[b]<br>*P* value[c]	Positively Selected Sites (BEB Analysis)[d]
**One ratio**	ω =0.04331	53	-2986.03		
**Branch-site Model**					
Branch e	site class 0 1 2a 2b<br>proportion 0.77203 0.04866 0.16868 0.01063<br>background w 0.03282 1.00000 0.03282 1.00000<br>foreground w 0.03282 1.00000 1.00000 1.00000	56	-2947.27	0<br>*P*=1	
Branch e<br>ω_a =1	site class 0 1 2a 2b<br>proportion 0.77203 0.04866 0.16868 0.01063<br>background w 0.03282 1.00000 0.03282 1.00000<br>foreground w 0.03282 1.00000 1.00000 1.00000	55	-2947.27		
Branch f	site class 0 1 2a 2b<br>proportion 0.74705 0.05088 0.18919 0.01288<br>background w 0.02898 1.00000 0.02898 1.00000<br>foreground w 0.02898 1.00000 1.00000 1.00000	56	-2945.24	0<br>*P*=1	
Branch f<br>ω_b =1	site class 0 1 2a 2b<br>proportion 0.74705 0.05088 0.18919 0.01288<br>background w 0.02898 1.00000 0.02898 1.00000<br>foreground w 0.02898 1.00000 1.00000 1.00000	55	-2945.24		
Branch g	site class 0 1 2a 2b<br>proportion 0.93982 0.06018 0.00000 0.00000<br>background w 0.03369 1.00000 0.03369 1.00000<br>foreground w 0.03369 1.00000 1.00000 1.00000	56	-2948.25	0<br>*P*=1	
Branch g<br>ω_c =1	site class 0 1 2a 2b<br>proportion 0.93982 0.06018 0.00000 0.00000<br>background w 0.03369 1.00000 0.03369 1.00000<br>foreground w 0.03369 1.00000 1.00000 1.00000	55	-2948.25		

12. ND5

Model	Parameter Estimated[a]	p	lnL	2⊿lnL[b]<br>*P* value[c]	Positively Selected Sites (BEB Analysis)[d]
**One ratio**	ω =0.0687	53	-21363.00		
**Branch-site Model**					
Branch e	site class 0 1 2a 2b<br>proportion 0.92484 0.07516 0.00000 0.00000<br>background w 0.05164 1.00000 0.05164 1.00000<br>foreground w 0.05164 1.00000 1.00000 1.00000	56	-21109.21	0<br>*P*=1	
Branch e	site class 0 1 2a 2b	55	-21109.21		

ω_a =1	proportion 0.92484 0.07516 0.00000 0.00000<br>background w 0.05164 1.00000 0.05164 1.00000<br>foreground w 0.05164 1.00000 1.00000 1.00000				
Branch f	site class 0 1 2a 2b<br>proportion 0.89077 0.06471 0.04150 0.00301<br>background w 0.05118 1.00000 0.05118 1.00000<br>foreground w 0.05118 1.00000 101.61172 101.61172	56	-21089.95	**14.4**<br>***P*<0.001**	342C 0.997<br>349S 0.986<br>443I 0.984<br>444N 0.997
Branch f<br>ω_b =1	site class 0 1 2a 2b<br>proportion 0.85810 0.06471 0.07178 0.00541<br>background w 0.05052 1.00000 0.05052 1.00000<br>foreground w 0.05052 1.00000 1.00000 1.00000	55	-21097.15		
Branch g	site class 0 1 2a 2b<br>proportion 0.92484 0.07516 0.00000 0.00000<br>background w 0.05164 1.00000 0.05164 1.00000<br>foreground w 0.05164 1.00000 2.78619 2.78619	56	-21109.21	0.08<br>*P*=0.78	
Branch g<br>ω_c =1	site class 0 1 2a 2b<br>proportion 0.92300 0.07499 0.00186 0.00015<br>background w 0.05159 1.00000 0.05159 1.00000<br>foreground w 0.05159 1.00000 1.00000 1.00000	55	-21109.17		

13. ND6

**Model**	**Parameter Estimated**[a]	**p**	**lnL**	**2⊿lnL**[b]<br>***P* value**[c]	**Positively Selected Sites (BEB Analysis)**[d]
**One ratio**	ω =0.0492	53	-6511.11		
**Branch-site Model**					
Branch e	site class 0 1 2a 2b<br>proportion 0.87929 0.04378 0.07327 0.00365<br>background w 0.04493 1.00000 0.04493 1.00000<br>foreground w 0.04493 1.00000 1.00000 1.00000	56	-6475.97	0<br>*P*=1	
Branch e<br>ω_a =1	site class 0 1 2a 2b<br>proportion 0.85289 0.04247 0.09968 0.00496<br>background w 0.04493 1.00000 0.04493 1.00000<br>foreground w 0.04493 1.00000 1.00000 1.00000	55	-6475.97		
Branch f	site class 0 1 2a 2b<br>proportion 0.44121 0.02188 0.51154 0.02537<br>background w 0.04117 1.00000 0.04117 1.00000<br>foreground w 0.04117 1.00000 3.16308 3.16308	56	-6443.19	49.18<br>*P*<0.001	3 Y 0.999<br>4 V 0.950<br>5 M 0.991<br>10 F 0.998<br>17 V 0.957<br>18 A 0.960<br>22 N 0.985<br>36 A 0.999<br>44 V 0.998<br>46 H 0.995<br>49 S 0.993
Branch f<br>ω_b =1	site class 0 1 2a 2b<br>proportion 0.00000 0.00000 0.95510 0.04490<br>background w 0.04291 1.00000 0.04291 1.00000<br>foreground w 0.04291 1.00000 1.00000 1.00000	55	-6467.78		

					68 A 0.970<br>81 S 0.964<br>82 W 0.996<br>86 S 0.999<br>87 R 0.997<br>88 M 0.980<br>89 G 0.991<br>90 Y 0.999<br>91 V 0.997<br>94 Y 0.998<br>110 Y 0.999<br>111 E 1.000<br>114 W 0.998<br>132L 0.998<br>134V 0.999<br>175iG 0.999
Branch g	site class 0 1 2a 2b<br>proportion 0.95015 0.04740 0.00234 0.00012<br>background w 0.04488 1.00000 0.04488 1.00000<br>foreground w 0.04488 1.00000 1.00000 1.00000	56	-6475.97	0<br>*P*=1	
Branch g<br>ω_c =1	site class 0 1 2a 2b<br>proportion 0.95015 0.04740 0.00234 0.00012<br>background w 0.04488 1.00000 0.04488 1.00000<br>foreground w 0.04488 1.00000 1.00000 1.00000	55	-6475.97		

[a] The proportion of sites estimated to have ω

[b] Twice the difference in log-likelihood values was calculated following a chi-squared (χ2)

[c] Posterior probability>0.99 of having ω>1

[d] The residues were identified by Bayes Empiriacal Bayes analysis

Supplementary Table 4: Selective pressure analyses for the 13 genes in Ophidiiformes.

1. ATP6

**Model**	**Parameter Estimated[a]**	**p**	**lnL**	**2⊿lnL[b]**<br>***P* value[c]**	**Positively Selected Sites (BEB Analysis)[d]**
**One ratio**	ω =0.0655	12	-3427.87		
**Branch-site Model**					
Branch h	site class 0 1 2a 2b<br>proportion 0.83321 0.04239 0.11837 0.00602<br>background w 0.05416 1.00000 0.05416 1.00000<br>foreground w 0.05416 1.00000 999.00000 999.00000	15	-3412.35	**6.52**<br>***P*=0.011**	
Branch h<br>$\omega_a = 1$	site class 0 1 2a 2b<br>proportion 0.00000 0.00000 0.94385 0.05615<br>background w 0.05809 1.00000 0.05809 1.00000<br>foreground w 0.05809 1.00000 1.00000 1.00000	14	-3415.61		

2. ATP8

**Model**	**Parameter Estimated[a]**	**p**	**lnL**	**2⊿lnL[b]**<br>***P* value[c]**	**Positively Selected Sites (BEB Analysis)[d]**
**One ratio**	ω =0.0822	12	-856.71		
**Branch-site Model**					
Branch h	site class 0 1 2a 2b<br>proportion 0.68491 0.00000 0.31509 0.00000<br>background w 0.07236 1.00000 0.07236 1.00000<br>foreground w 0.07236 1.00000 481.56590 481.56590	15	-855.25	0.66<br>*P*=0.42	
Branch h<br>$\omega_a = 1$	site class 0 1 2a 2b<br>proportion 0.71824 0.00000 0.28176 0.00000<br>background w 0.07382 1.00000 0.07382 1.00000<br>foreground w 0.07382 1.00000 1.00000 1.00000	14	-855.58		

3. CoxI

**Model**	**Parameter Estimated[a]**	**p**	**lnL**	**2⊿lnL[b]**<br>***P* value[c]**	**Positively Selected Sites (BEB Analysis)[d]**
**One ratio**	ω =0.0165	12	-5331.74		
**Branch-site Model**					
Branch h	site class 0 1 2a 2b<br>proportion 0.97762 0.00838 0.01388 0.00012<br>background w 0.01385 1.00000 0.01385 1.00000<br>foreground w 0.01385 1.00000 3.32788 3.32788	15	-5311.15	0.94<br>*P*=0.33	
Branch h<br>$\omega_a = 1$	site class 0 1 2a 2b<br>proportion 0.97004 0.00831 0.02147 0.00018<br>background w 0.01373 1.00000 0.01373 1.00000<br>foreground w 0.01373 1.00000 1.00000 1.00000	14	-5311.62		

4. CoxII

Model	Parameter Estimated[a]	p	lnL	2⊿lnL[b]<br>*P* value[c]	Positively Selected Sites (BEB Analysis)[d]
**One ratio**	ω =0.0307	12	-2558.79		
**Branch-site Model**					
Branch h	site class 0 1 2a 2b<br>proportion 0.97868 0.01548 0.00575 0.00009<br>background w 0.02720 1.00000 0.02720 1.00000<br>foreground w 0.02720 1.00000 1.68869 1.68869	15	-2555.52	0.12<br>*P*=0.73	
Branch h<br>ω_a = 1	site class 0 1 2a 2b<br>proportion 0.98426 0.01574 0.00000 0.00000<br>background w 0.02738 1.00000 0.02738 1.00000<br>foreground w 0.02738 1.00000 1.00000 1.00000	14	-2555.58		

5. CoxIII

Model	Parameter Estimated[a]	p	lnL	2⊿lnL[b]<br>*P* value[c]	Positively Selected Sites (BEB Analysis)[d]
**One ratio**	ω =0.0238	12	-2777.17		
**Branch-site Model**					
Branch h	site class 0 1 2a 2b<br>proportion 0.00000 0.00000 0.96679 0.03321<br>background w 0.01718 1.00000 0.01718 1.00000<br>foreground w 0.01718 1.00000 1.00000 1.00000	15	-2766.76	0<br>*P*=1	
Branch h<br>ω_a = 1	site class 0 1 2a 2b<br>proportion 0.00000 0.00000 0.96679 0.03321<br>background w 0.01718 1.00000 0.01718 1.00000<br>foreground w 0.01718 1.00000 1.00000 1.00000	14	-2766.76		

6. CytB

Model	Parameter Estimated[a]	p	lnL	2⊿lnL[b]<br>*P* value[c]	Positively Selected Sites (BEB Analysis)[d]
**One ratio**	ω =0.0272	12	-4928.38		
**Branch-site Model**					
Branch h	site class 0 1 2a 2b<br>proportion 0.86877 0.02131 0.10729 0.00263<br>background w 0.02310 1.00000 0.02310 1.00000<br>foreground w 0.02310 1.00000 12.47132 12.47132	15	-4913.17	0.14<br>*P*=0.71	
Branch h<br>ω_a = 1	site class 0 1 2a 2b<br>proportion 0.88965 0.02205 0.08616 0.00214<br>background w 0.02317 1.00000 0.02317 1.00000<br>foreground w 0.02317 1.00000 1.00000 1.00000	14	-4913.24		

7. ND1

Model	Parameter Estimated[a]	p	lnL	2⊿lnL[b]<br>*P* value[c]	Positively Selected Sites (BEB Analysis)[d]

**One ratio**	ω =0.0276	12	-4153.15		
**Branch-site Model**					
Branch h	site class 0 1 2a 2b<br>proportion 0.92996 0.06312 0.00648 0.00044<br>background w 0.02005 1.00000 0.02005 1.00000<br>foreground w 0.02005 1.00000 14.40980 14.40980	15	-4116.35	0.98<br>*P*=0.32	
Branch h<br>$\omega_a = 1$	site class 0 1 2a 2b<br>proportion 0.92618 0.06585 0.00744 0.00053<br>background w 0.02011 1.00000 0.02011 1.00000<br>foreground w 0.02011 1.00000 1.00000 1.00000	14	-4116.84		

8. ND2

Model	Parameter Estimated[a]	p	lnL	2⊿lnL[b]<br>*P* value[c]	Positively Selected Sites (BEB Analysis)[d]
**One ratio**	ω =0.0435	12	-5377.08		
**Branch-site Model**					
Branch h	site class 0 1 2a 2b<br>proportion 0.84231 0.07151 0.07944 0.00674<br>background w 0.03378 1.00000 0.03378 1.00000<br>foreground w 0.03378 1.00000 5.45510 5.45510	15	-5340.41	1.06<br>*P*=0.30	
Branch h<br>$\omega_a = 1$	site class 0 1 2a 2b<br>proportion 0.85223 0.07233 0.06954 0.00590<br>background w 0.03338 1.00000 0.03338 1.00000<br>foreground w 0.03338 1.00000 1.00000 1.00000	14	-5340.94		

9. ND3

Model	Parameter Estimated[a]	p	lnL	2⊿lnL[b]<br>*P* value[c]	Positively Selected Sites (BEB Analysis)[d]
**One ratio**	ω =0.0227	12	-1508.93		
**Branch-site Model**					
Branch h	site class 0 1 2a 2b<br>proportion 0.00000 0.00000 0.92427 0.07573<br>background w 0.01065 1.00000 0.01065 1.00000<br>foreground w 0.01065 1.00000 1.00000 1.00000	15	-1499.02	0<br>*P*=1	
Branch h<br>$\omega_a = 1$	site class 0 1 2a 2b<br>proportion 0.00010 0.00001 0.92417 0.07572<br>background w 0.01065 1.00000 0.01065 1.00000<br>foreground w 0.01065 1.00000 1.00000 1.00000	14	-1499.02		

10. ND4

Model	Parameter Estimated[a]	p	lnL	2⊿lnL[b]<br>*P* value[c]	Positively Selected Sites (BEB Analysis)[d]
**One ratio**	ω =0.0355	12	-6004.53		
**Branch-site Model**					
Branch h	site class 0 1 2a 2b	15	-5984.90	1.3	

	proportion 0.00000 0.00000 0.96715 0.03285<br>background w 0.02950 1.00000 0.02950 1.00000<br>foreground w 0.02950 1.00000 138.16853 138.16853			*P*=0.25	
Branch h<br>ω_a = 1	site class 0 1 2a 2b<br>proportion 0.83661 0.02743 0.13164 0.00432<br>background w 0.03355 1.00000 0.03355 1.00000<br>foreground w 0.03355 1.00000 1.00000 1.00000	14	-5985.55		

11. ND4L

Model	Parameter Estimated[a]	p	lnL	2⊿lnL[b]<br>*P* value[c]	Positively Selected Sites (BEB Analysis)[d]
**One ratio**	ω =0.0244	12	-1245.78		
**Branch-site Model**					
Branch h	site class 0 1 2a 2b<br>proportion 0.00000 0.00000 0.98578 0.01422<br>background w 0.02353 1.00000 0.02353 1.00000<br>foreground w 0.02353 1.00000 999.00000 999.00000	15	-1243.16	0.04<br>*P*=0.84	
Branch h<br>ω_a = 1	site class 0 1 2a 2b<br>proportion 0.00000 0.00000 0.98580 0.01420<br>background w 0.02344 1.00000 0.02344 1.00000<br>foreground w 0.02344 1.00000 1.00000 1.00000	14	-1243.18		

12. ND5

Model	Parameter Estimated[a]	p	lnL	2⊿lnL[b]<br>*P* value[c]	Positively Selected Sites (BEB Analysis)[d]
**One ratio**	ω =0.0538	12	-8310.33		
**Branch-site Model**					
Branch h	site class 0 1 2a 2b<br>proportion 0.38781 0.03224 0.53545 0.04451<br>background w 0.03376 1.00000 0.03376 1.00000<br>foreground w 0.03376 1.00000 999.00000 999.00000	15	-8216.16	0.12<br>*P*=0.73	
Branch h<br>ω_a = 1	site class 0 1 2a 2b<br>proportion 0.00000 0.00000 0.92326 0.07674<br>background w 0.03384 1.00000 0.03384 1.00000<br>foreground w 0.03384 1.00000 1.00000 1.00000	14	-8216.22		

13. ND6

Model	Parameter Estimated[a]	p	lnL	2⊿lnL[b]<br>*P* value[c]	Positively Selected Sites (BEB Analysis)[d]
**One ratio**	ω =0.0377	12	-2508.04		
**Branch-site Model**					
Branch h	site class 0 1 2a 2b<br>proportion 0.86204 0.05362 0.07940 0.00494<br>background w 0.02950 1.00000 0.02950 1.00000<br>foreground w 0.02950 1.00000 999.00000 999.00000	15	-2495.35	**5.54**<br>***P*=0.019**	

Branch h<br>$\omega_a = 1$	site class 0 1 2a 2b<br>proportion 0.00000 0.00000 0.94807 0.05193<br>background w 0.03001 1.00000 0.03001 1.00000<br>foreground w 0.03001 1.00000 1.00000 1.00000	14	-2498.12		

[a] The proportion of sites estimated to have ω

[b] Twice the difference in log-likelihood values was calculated following a chi-squared ($\chi 2$)

[c] Posterior probability>0.99 of having ω>1

[d] The residues were identified by Bayes Empiriacal Bayes analysis

Supplementary Table 5: Selective pressure analyses for the 13 genes in Osmeriformes and Stomiiformes.

1. ATP6

**Model**	**Parameter Estimated**[a]	**p**	**lnL**	**2⊿lnL**[b]<br>***P* value**[c]	**Positively Selected Sites (BEB Analysis)**[d]
**One ratio**	0.0403	86	-12019.85		
**Branch-site Model**					
Branch i	site class 0 1 2a 2b<br>proportion 0.94624 0.05376 0.00000 0.00000<br>background w 0.03159 1.00000 0.03159 1.00000<br>foreground w 0.03159 1.00000 1.00000 1.00000	89	-11955.86	0	
Branch i<br>ω_a =1	site class 0 1 2a 2b<br>proportion 0.94624 0.05376 0.00000 0.00000<br>background w 0.03159 1.00000 0.03159 1.00000<br>foreground w 0.03159 1.00000 1.00000 1.00000	88	-11955.86		
Branch j	site class 0 1 2a 2b<br>proportion 0.92780 0.05330 0.01787 0.00103<br>background w 0.03146 1.00000 0.03146 1.00000<br>foreground w 0.03146 1.00000 690.28875 690.28875	89	-11952.75	3.34<br>*P*=0.068	172G 0.980<br>175 S 0.519
Branch j<br>ω_b =1	site class 0 1 2a 2b<br>proportion 0.90975 0.05191 0.03626 0.00207<br>background w 0.03129 1.00000 0.03129 1.00000<br>foreground w 0.03129 1.00000 1.00000 1.00000	88	-11954.42		

2. ATP8

**Model**	**Parameter Estimated**[a]	**p**	**lnL**	**2⊿lnL**[b]<br>***P* value**[c]	**Positively Selected Sites (BEB Analysis)**[d]
**One ratio**	0.0994	86	-2650.91		
**Branch-site Model**					
Branch i	site class 0 1 2a 2b<br>proportion 0.91131 0.08869 0.00000 0.00000<br>background w 0.08815 1.00000 0.08815 1.00000<br>foreground w 0.08815 1.00000 1.00000 1.00000	89	-2641.39	0	
Branch i<br>ω_a =1	site class 0 1 2a 2b<br>proportion 0.91131 0.08869 0.00000 0.00000<br>background w 0.08815 1.00000 0.08815 1.00000<br>foreground w 0.08815 1.00000 1.00000 1.00000	88	-2641.39		
Branch j	site class 0 1 2a 2b<br>proportion 0.00000 0.00000 0.92093 0.07907<br>background w 0.08844 1.00000 0.08844 1.00000<br>foreground w 0.08844 1.00000 92.28273 92.28273	89	-2641.25	0.08<br>*P*=0.78	
Branch j<br>ω_b =1	site class 0 1 2a 2b<br>proportion 0.00000 0.00000 0.91877 0.08123<br>background w 0.08836 1.00000 0.08836 1.00000	88	-2641.29		

	foreground w 0.08836 1.00000 1.00000 1.00000				

3. CoxI

Model	Parameter Estimated[a]	p	lnL	2⊿lnL[b]<br>*P* value[c]	Positively Selected Sites (BEB Analysis)[d]
**One ratio**	0.0178	86	-21297.85		
**Branch-site Model**					
Branch i	site class 0 1 2a 2b<br>proportion 0.98896 0.01104 0.00000 0.00000<br>background w 0.01561 1.00000 0.01561 1.00000<br>foreground w 0.01561 1.00000 1.00000 1.00000	89	-21241.58	0<br>*P*=1	
Branch i<br>ω_a =1	site class 0 1 2a 2b<br>proportion 0.98896 0.01104 0.00000 0.00000<br>background w 0.01561 1.00000 0.01561 1.00000<br>foreground w 0.01561 1.00000 1.00000 1.00000	88	-21241.58		
Branch j	site class 0 1 2a 2b<br>proportion 0.80480 0.00899 0.18416 0.00206<br>background w 0.01561 1.00000 0.01561 1.00000<br>foreground w 0.01561 1.00000 4.09075 4.09075	89	-21241.58	0<br>*P*=1	
Branch j<br>ω_b =1	site class 0 1 2a 2b<br>proportion 0.90962 0.01016 0.07934 0.00089<br>background w 0.01561 1.00000 0.01561 1.00000<br>foreground w 0.01561 1.00000 1.00000 1.00000	88	-21241.58		

4. CoxII

Model	Parameter Estimated[a]	p	lnL	2⊿lnL[b]<br>*P* value[c]	Positively Selected Sites (BEB Analysis)[d]
**One ratio**	0.0328	86	-9169.45		
**Branch-site Model**					
Branch i	site class 0 1 2a 2b<br>proportion 0.88962 0.01569 0.09304 0.00164<br>background w 0.02727 1.00000 0.02727 1.00000<br>foreground w 0.02727 1.00000 1.00000 1.00000	89	-9126.45	0<br>*P*=1	
Branch i<br>ω_a =1	site class 0 1 2a 2b<br>proportion 0.88962 0.01569 0.09304 0.00164<br>background w 0.02727 1.00000 0.02727 1.00000<br>foreground w 0.02727 1.00000 1.00000 1.00000	88	-9126.45		
Branch j	site class 0 1 2a 2b<br>proportion 0.00000 0.00000 0.98278 0.01722<br>background w 0.02839 1.00000 0.02839 1.00000<br>foreground w 0.02839 1.00000 14.15526 14.15526	89	-9129.03	0.02<br>*P*=0.89	
Branch j<br>ω_b =1	site class 0 1 2a 2b<br>proportion 0.00000 0.00000 0.98277 0.01723<br>background w 0.02839 1.00000 0.02839 1.00000	88	-9129.04		

	foreground w 0.02839 1.00000 1.00000 1.00000				

5. CoxIII

Model	Parameter Estimated[a]	p	lnL	2⊿lnL[b]<br>*P* value[c]	Positively Selected Sites (BEB Analysis)[d]
**One ratio**	0.0303	86	-11342.06		
**Branch-site Model**					
Branch i	site class 0 1 2a 2b<br>proportion 0.94420 0.05121 0.00435 0.00024<br>background w 0.02029 1.00000 0.02029 1.00000<br>foreground w 0.02029 1.00000 199.31269 199.31269	89	-11198.58	**4.58**<br>***P*=0.032**	31 L 0.994
Branch i<br>ω_a =1	site class 0 1 2a 2b<br>proportion 0.94043 0.05057 0.00854 0.00046<br>background w 0.02033 1.00000 0.02033 1.00000<br>foreground w 0.02033 1.00000 1.00000 1.00000	88	-11200.87		
Branch j	site class 0 1 2a 2b<br>proportion 0.92303 0.04985 0.02573 0.00139<br>background w 0.02034 1.00000 0.02034 1.00000<br>foreground w 0.02034 1.00000 1.00000 1.00000	89	-11202.16	0<br>*P*=1	
Branch j<br>ω_b =1	site class 0 1 2a 2b<br>proportion 0.92303 0.04985 0.02573 0.00139<br>background w 0.02034 1.00000 0.02034 1.00000<br>foreground w 0.02034 1.00000 1.00000 1.00000	88	-11202.16		

6. CytB

Model	Parameter Estimated[a]	p	lnL	2⊿lnL[b]<br>*P* value[c]	Positively Selected Sites (BEB Analysis)[d]
**One ratio**	0.0486	86	-19811.22		
**Branch-site Model**					
Branch i	site class 0 1 2a 2b<br>proportion 0.93772 0.05392 0.00790 0.00045<br>background w 0.04187 1.00000 0.04187 1.00000<br>foreground w 0.04187 1.00000 12.21007 12.21007	89	-19664.54	**4.72**<br>***P*=0.0298**	12 K 0.990
Branch i<br>ω_a =1	site class 0 1 2a 2b<br>proportion 0.92719 0.05329 0.01846 0.00106<br>background w 0.04183 1.00000 0.04183 1.00000<br>foreground w 0.04183 1.00000 1.00000 1.00000	88	-19666.90		
Branch j	site class 0 1 2a 2b<br>proportion 0.93053 0.05364 0.01496 0.00086<br>background w 0.04191 1.00000 0.04191 1.00000<br>foreground w 0.04191 1.00000 5.14313 5.14313	89	-19665.42	-0.04	123 T 0.968<br>372 I 0.957
Branch j<br>ω_b =1	site class 0 1 2a 2b<br>proportion 0.90752 0.05230 0.03799 0.00219<br>background w 0.04188 1.00000 0.04188 1.00000	88	-19666.40		

	foreground w 0.04188 1.00000 1.00000 1.00000				

7. ND1

Model	Parameter Estimated[a]	p	lnL	2⊿lnL[b]<br>*P* value[c]	Positively Selected Sites (BEB Analysis)[d]
**One ratio**	0.0287	86	-15652.39		
**Branch-site Model**					
Branch i	site class 0 1 2a 2b<br>proportion 0.96952 0.03048 0.00000 0.00000<br>background w 0.02338 1.00000 0.02338 1.00000<br>foreground w 0.02338 1.00000 1.00000 1.00000	89	-15548.17	0<br>*P*=1	
Branch i<br>ω_a =1	site class 0 1 2a 2b<br>proportion 0.96952 0.03048 0.00000 0.00000<br>background w 0.02338 1.00000 0.02338 1.00000<br>foreground w 0.02338 1.00000 1.00000 1.00000	88	-15548.17		
Branch j	site class 0 1 2a 2b<br>proportion 0.96952 0.03048 0.00000 0.00000<br>background w 0.02338 1.00000 0.02338 1.00000<br>foreground w 0.02338 1.00000 1.00000 1.00000	89	-15548.17	0<br>*P*=1	
Branch j<br>ω_b =1	site class 0 1 2a 2b<br>proportion 0.96952 0.03048 0.00000 0.00000<br>background w 0.02338 1.00000 0.02338 1.00000<br>foreground w 0.02338 1.00000 1.00000 1.00000	88	-15548.17		

8. ND2

Model	Parameter Estimated[a]	p	lnL	2⊿lnL[b]<br>*P* value[c]	Positively Selected Sites (BEB Analysis)[d]
**One ratio**	0.0669	86	-20896.95		
**Branch-site Model**					
Branch i	site class 0 1 2a 2b<br>proportion 0.91828 0.06976 0.01111 0.00084<br>background w 0.05435 1.00000 0.05435 1.00000<br>foreground w 0.05435 1.00000 2.26117 2.26117	89	-20723.90	1<br>*P*=0.32	
Branch i<br>ω_a =1	site class 0 1 2a 2b<br>proportion 0.92715 0.07285 0.00000 0.00000<br>background w 0.05445 1.00000 0.05445 1.00000<br>foreground w 0.05445 1.00000 1.00000 1.00000	88	-20724.40		
Branch j	site class 0 1 2a 2b<br>proportion 0.78543 0.05866 0.14508 0.01083<br>background w 0.05324 1.00000 0.05324 1.00000<br>foreground w 0.05324 1.00000 999.00000 999.00000	89	-20694.94	**22.38**<br>**P<0.001**	2 H 0.996<br>8 C 0.977<br>12 A 0.961<br>41 L 0.995<br>75 L 1.000<br>100 L 0.976<br>150 D 0.987
Branch j<br>ω_b =1	site class 0 1 2a 2b<br>proportion 0.73542 0.05585 0.19400 0.01473<br>background w 0.05318 1.00000 0.05318 1.00000	88	-20706.13		

	foreground w 0.05318 1.00000 1.00000 1.00000				224 C 0.997<br>229 G 0.983<br>244 L 1.000<br>246 P 0.977<br>267 I 0.995<br>268 D 0.998<br>271 V 1.000<br>285 S 0.996<br>304 S 0.999<br>311 K 0.998<br>322 G 0.968<br>330 S 0.996

9. ND3

**Model**	**Parameter Estimated**[a]	**p**	**lnL**	**2⊿lnL**[b]<br>***P* value**[c]	**Positively Selected Sites (BEB Analysis)**[d]
**One ratio**	0.0560	86	-6080.65		
**Branch-site Model**					
Branch i	site class 0 1 2a 2b<br>proportion 0.81936 0.18064 0.00000 0.00000<br>background w 0.02102 1.00000 0.02102 1.00000<br>foreground w 0.02102 1.00000 1.00000 1.00000	89	-5957.50	0<br>*P*=1	
Branch i<br>ω_a =1	site class 0 1 2a 2b<br>proportion 0.81936 0.18064 0.00000 0.00000<br>background w 0.02102 1.00000 0.02102 1.00000<br>foreground w 0.02102 1.00000 1.00000 1.00000	88	-5957.50		
Branch j	site class 0 1 2a 2b<br>proportion 0.66993 0.14769 0.14943 0.03294<br>background w 0.02102 1.00000 0.02102 1.00000<br>foreground w 0.02102 1.00000 1.09013 1.09013	89	-5957.50	0<br>*P*=1	
Branch j<br>ω_b =1	site class 0 1 2a 2b<br>proportion 0.66492 0.14659 0.15445 0.03405<br>background w 0.02102 1.00000 0.02102 1.00000<br>foreground w 0.02102 1.00000 1.00000 1.00000	88	-5957.50		

10. ND4

**Model**	**Parameter Estimated**[a]	**p**	**lnL**	**2⊿lnL**[b]<br>***P* value**[c]	**Positively Selected Sites (BEB Analysis)**[d]
**One ratio**	0.0432	86	-24293.39		
**Branch-site Model**					
Branch i	site class 0 1 2a 2b<br>proportion 0.96035 0.03965 0.00000 0.00000<br>background w 0.03752 1.00000 0.03752 1.00000<br>foreground w 0.03752 1.00000 1.00000 1.00000	89	-24195.20	0<br>*P*=1	
Branch i	site class 0 1 2a 2b	88	-24195.20		

ω_a =1	proportion 0.96035 0.03965 0.00000 0.00000<br>background w 0.03752 1.00000 0.03752 1.00000<br>foreground w 0.03752 1.00000 1.00000 1.00000				
Branch j	site class 0 1 2a 2b<br>proportion 0.91946 0.03709 0.04176 0.00168<br>background w 0.03707 1.00000 0.03707 1.00000<br>foreground w 0.03707 1.00000 4.28674 4.28674	89	-24185.81	3.58<br>*P*=0.058	256 N 0.983<br>345 M 0.990
Branch j<br>ω_b =1	site class 0 1 2a 2b<br>proportion 0.88984 0.03623 0.07104 0.00289<br>background w 0.03698 1.00000 0.03698 1.00000<br>foreground w 0.03698 1.00000 1.00000 1.00000	88	-24187.60		

11. ND4L

**Model**	**Parameter Estimated[a]**	**p**	**lnL**	**2⊿lnL[b]**<br>***P* value[c]**	**Positively Selected Sites (BEB Analysis)[d]**
**One ratio**	0.0359	86	-4600.04		
**Branch-site Model**					
Branch i	site class 0 1 2a 2b<br>proportion 0.95180 0.04820 0.00000 0.00000<br>background w 0.02982 1.00000 0.02982 1.00000<br>foreground w 0.02982 1.00000 1.00000 1.00000	89	-4562.04	0<br>*P*=1	
Branch i<br>ω_a =1	site class 0 1 2a 2b<br>proportion 0.95180 0.04820 0.00000 0.00000<br>background w 0.02982 1.00000 0.02982 1.00000<br>foreground w 0.02982 1.00000 1.00000 1.00000	88	-4562.04		
Branch j	site class 0 1 2a 2b<br>proportion 0.90996 0.04637 0.04156 0.00212<br>background w 0.02942 1.00000 0.02942 1.00000<br>foreground w 0.02942 1.00000 1.00000 1.00000	89	-4561.89	0<br>*P*=1	
Branch j<br>ω_b =1	site class 0 1 2a 2b<br>proportion 0.90996 0.04637 0.04156 0.00212<br>background w 0.02942 1.00000 0.02942 1.00000<br>foreground w 0.02942 1.00000 1.00000 1.00000	88	-4561.89		

12. ND5

**Model**	**Parameter Estimated[a]**	**p**	**lnL**	**2⊿lnL[b]**<br>***P* value[c]**	**Positively Selected Sites (BEB Analysis)[d]**
**One ratio**	0.0514	86	-32238.87		
**Branch-site Model**					
Branch i	site class 0 1 2a 2b<br>proportion 0.95280 0.04720 0.00000 0.00000<br>background w 0.04202 1.00000 0.04202 1.00000<br>foreground w 0.04202 1.00000 1.00000 1.00000	89	-31944.51	0<br>*P*=1	
Branch i	site class 0 1 2a 2b	88	-31944.51		

ω_a =1	proportion 0.95280 0.04720 0.00000 0.00000<br>background w 0.04202 1.00000 0.04202 1.00000<br>foreground w 0.04202 1.00000 1.00000 1.00000				
Branch j	site class 0 1 2a 2b<br>proportion 0.83032 0.04198 0.12155 0.00615<br>background w 0.04132 1.00000 0.04132 1.00000<br>foreground w 0.04132 1.00000 9.05791 9.05791	89	-31935.98	**5.18**<br>***P*=0.023**	17L 0.999<br>424 T 0.971<br>555 L 0.957<br>589G 0.954
Branch j<br>ω_b =1	site class 0 1 2a 2b<br>proportion 0.00055 0.00003 0.95113 0.04830<br>background w 0.04133 1.00000 0.04133 1.00000<br>foreground w 0.04133 1.00000 1.00000 1.00000	88	-31938.57		

13. ND6

**Model**	**Parameter Estimated**[a]	**p**	**lnL**	**2⊿lnL**[b]<br>***P* value**[c]	**Positively Selected Sites (BEB Analysis)**[d]
**One ratio**	0.0586	86	-9473.88		
**Branch-site Model**					
Branch i	site class 0 1 2a 2b<br>proportion 0.89609 0.06435 0.03691 0.00265<br>background w 0.04709 1.00000 0.04709 1.00000<br>foreground w 0.04709 1.00000 29.70776 29.70776	89	-9381.84	**15.06**<br>***P*<0.001**	105 F 0.995<br>107 G 0.998<br>113 P 0.998
Branch i<br>ω_a =1	site class 0 1 2a 2b<br>proportion 0.85865 0.06159 0.07442 0.00534<br>background w 0.04689 1.00000 0.04689 1.00000<br>foreground w 0.04689 1.00000 1.00000 1.00000	88	-9389.37		
Branch j	site class 0 1 2a 2b<br>proportion 0.91341 0.06666 0.01857 0.00136<br>background w 0.04838 1.00000 0.04838 1.00000<br>foreground w 0.04838 1.00000 26.33134 26.33134	89	-9394.84	2.32<br>*P*=0.13	
Branch j<br>ω_b =1	site class 0 1 2a 2b<br>proportion 0.87965 0.06398 0.05255 0.00382<br>background w 0.04827 1.00000 0.04827 1.00000<br>foreground w 0.04827 1.00000 1.00000 1.00000	88	-9396.00		

[a] The proportion of sites estimated to have ω

[b] Twice the difference in log-likelihood values was calculated following a chi-squared ($\chi 2$)

[c] Posterior probability>0.99 of having ω>1

[d] The residues were identified by Bayes Empiriacal Bayes analysis

Supplementary Table 6: Selective pressure analyses for the 13 genes in Perciformes.

1. ATP6

Model	Parameter Estimated[a]	p	lnL	2⊿lnL[b]<br>*P* value[c]	Positively Selected Sites (BEB Analysis)[d]
**One ratio**	ω =0.0368	14	-968.14		
**Branch-site Model**					
Branch k	site class 0 1 2a 2b<br>proportion 1.00000 0.00000 0.00000 0.00000<br>background w 0.03675 1.00000 0.03675 1.00000<br>foreground w 0.03675 1.00000 1.06005 1.06005	17	-968.14	0<br>*P*=1	
Branch k<br>ω_a = 1	site class 0 1 2a 2b<br>proportion 1.00000 0.00000 0.00000 0.00000<br>background w 0.03675 1.00000 0.03675 1.00000<br>foreground w 0.03675 1.00000 1.00000 1.00000	16	-968.14		
**One ratio**	ω =0.0304	16	-2720.92		
**Branch-site Model**					
Branch l	site class 0 1 2a 2b<br>proportion 0.98255 0.01745 0.00000 0.00000<br>background w 0.02596 1.00000 0.02596 1.00000<br>foreground w 0.02596 1.00000 1.00000 1.00000	19	-2717.12	0<br>*P*=1	
Branch l<br>ω_a = 1	site class 0 1 2a 2b<br>proportion 0.98255 0.01745 0.00000 0.00000<br>background w 0.02596 1.00000 0.02596 1.00000<br>foreground w 0.02596 1.00000 1.00000 1.00000	18	-2717.12		
**One ratio**	ω =0.0145	12	-1884.80		
**Branch-site Model**					
Branch m	site class 0 1 2a 2b<br>proportion 0.96274 0.03726 0.00000 0.00000<br>background w 0.00656 1.00000 0.00656 1.00000<br>foreground w 0.00656 1.00000 1.00000 1.00000	15	-1872.28	0<br>*P*=1	
Branch m<br>ω_a = 1	site class 0 1 2a 2b<br>proportion 0.96274 0.03726 0.00000 0.00000<br>background w 0.00656 1.00000 0.00656 1.00000<br>foreground w 0.00656 1.00000 1.00000 1.00000	14	-1872.28		

2. ATP8

Model	Parameter Estimated[a]	p	lnL	2⊿lnL[b]<br>*P* value[c]	Positively Selected Sites (BEB Analysis)[d]
**One ratio**	ω =0.0001	14	-198.23		
**Branch-site Model**					
Branch k	site class 0 1 2a 2b<br>proportion 0.56800 0.22275 0.15031 0.05894<br>background w 0.24647 1.00000 0.24647 1.00000	17	-198.23	0<br>*P*=1	

	foreground w 0.24647 1.00000 1.06630 1.06630				
Branch k<br>ω_a = 1	site class 0 1 2a 2b<br>proportion 0.70797 0.29196 0.00005 0.00002<br>background w 0.00000 1.00000 0.00000 1.00000<br>foreground w 0.00000 1.00000 1.00000 1.00000	16	-198.23		
**One ratio**	ω =0.1021	18	-607.55		
**Branch-site Model**					
Branch l	site class 0 1 2a 2b<br>proportion 0.94166 0.05834 0.00000 0.00000<br>background w 0.07604 1.00000 0.07604 1.00000<br>foreground w 0.07604 1.00000 1.00000 1.00000	21	-604.55	0<br>*P*=1	
Branch l<br>ω_a = 1	site class 0 1 2a 2b<br>proportion 0.94166 0.05834 0.00000 0.00000<br>background w 0.07604 1.00000 0.07604 1.00000<br>foreground w 0.07604 1.00000 1.00000 1.00000	20	-604.55		
**One ratio**	ω =0.0832	12	-409.37		
**Branch-site Model**					
Branch m	site class 0 1 2a 2b<br>proportion 1.00000 0.00000 0.00000 0.00000<br>background w 0.08315 1.00000 0.08315 1.00000<br>foreground w 0.08315 1.00000 1.00000 1.00000	15	-409.37	0<br>*P*=1	
Branch m<br>ω_a = 1	site class 0 1 2a 2b<br>proportion 1.00000 0.00000 0.00000 0.00000<br>background w 0.08315 1.00000 0.08315 1.00000<br>foreground w 0.08315 1.00000 1.00000 1.00000	14	-409.37		

3. CoxI

**Model**	**Parameter Estimated**[a]	**p**	**lnL**	**2⊿lnL**[b]<br>***P* value**[c]	**Positively Selected Sites (BEB Analysis)**[d]
**One ratio**	ω =0.0604	14	-2161.85		
**Branch-site Model**					
Branch k	site class 0 1 2a 2b<br>proportion 0.95156 0.04790 0.00052 0.00003<br>background w 0.00000 1.00000 0.00000 1.00000<br>foreground w 0.00000 1.00000 1.00000 1.00000	17	-2159.09	0<br>*P*=1	
Branch k<br>ω_a = 1	site class 0 1 2a 2b<br>proportion 0.84101 0.04233 0.11107 0.00559<br>background w 0.00000 1.00000 0.00000 1.00000<br>foreground w 0.00000 1.00000 1.00000 1.00000	16	-2159.09		
**One ratio**	ω = 0.0151	18	-5486.16		
**Branch-site Model**					
Branch l	site class 0 1 2a 2b<br>proportion 0.99642 0.00358 0.00000 0.00000<br>background w 0.01364 1.00000 0.01364 1.00000	21	-5481.18	0<br>*P*=1	

	foreground w 0.01364 1.00000 1.00000 1.00000				
Branch l<br>ω_a = 1	site class 0 1 2a 2b<br>proportion 0.99642 0.00358 0.00000 0.00000<br>background w 0.01364 1.00000 0.01364 1.00000<br>foreground w 0.01364 1.00000 1.00000 1.00000	20	-5481.18		
**One ratio**	ω =0.0113	12	-3817.42		
**Branch-site Model**					
Branch m	site class 0 1 2a 2b<br>proportion 0.97838 0.00000 0.02162 0.00000<br>background w 0.01071 1.00000 0.01071 1.00000<br>foreground w 0.01071 1.00000 1.00000 1.00000	15	-3816.71	0<br>*P*=1	
Branch m<br>ω_a = 1	site class 0 1 2a 2b<br>proportion 0.97838 0.00000 0.02162 0.00000<br>background w 0.01071 1.00000 0.01071 1.00000<br>foreground w 0.01071 1.00000 1.00000 1.00000	14	-3816.71		

4. CoxII

**Model**	**Parameter Estimated**[a]	**p**	**lnL**	**2⊿lnL**[b]<br>***P* value**[c]	**Positively Selected Sites (BEB Analysis)**[d]
**One ratio**	ω =0.0572	14	-947.15		
**Branch-site Model**					
Branch k	site class 0 1 2a 2b<br>proportion 1.00000 0.00000 0.00000 0.00000<br>background w 0.05725 1.00000 0.05725 1.00000<br>foreground w 0.05725 1.00000 1.05899 1.05899	17	-947.15	0.02<br>*P*= 0.89	
Branch k<br>ω_a = 1	site class 0 1 2a 2b<br>proportion 1.00000 0.00000 0.00000 0.00000<br>background w 0.05731 1.00000 0.05731 1.00000<br>foreground w 0.05731 1.00000 1.00000 1.00000	16	-947.14		
**One ratio**	ω =0.0206	18	-2298.65		
**Branch-site Model**					
Branch l	site class 0 1 2a 2b<br>proportion 0.98944 0.01056 0.00000 0.00000<br>background w 0.01667 1.00000 0.01667 1.00000<br>foreground w 0.01667 1.00000 1.00000 1.00000	21	-2296.82	0<br>*P*=1	
Branch l<br>ω_a = 1	site class 0 1 2a 2b<br>proportion 0.98944 0.01056 0.00000 0.00000<br>background w 0.01667 1.00000 0.01667 1.00000<br>foreground w 0.01667 1.00000 1.00000 1.00000	20	-2296.82		
**One ratio**	ω =0.0087	12	-1558.01		
**Branch-site Model**					
Branch m	site class 0 1 2a 2b<br>proportion 1.00000 0.00000 0.00000 0.00000<br>background w 0.00870 1.00000 0.00870 1.00000	15	-1558.01	0<br>*P*=1	

	foreground w 0.00870 1.00000 1.00000 1.00000				
Branch m<br>$\omega_a = 1$	site class 0 1 2a 2b<br>proportion 1.00000 0.00000 0.00000 0.00000<br>background w 0.00870 1.00000 0.00870 1.00000<br>foreground w 0.00870 1.00000 1.00000 1.00000	14	-1558.01		

5. CoxIII

Model	Parameter Estimated[a]	p	lnL	2⊿lnL[b]<br>*P* value[c]	Positively Selected Sites (BEB Analysis)[d]
**One ratio**	ω =0.0598	14	-1123.14		
**Branch-site Model**					
Branch k	site class 0 1 2a 2b<br>proportion 0.99998 0.00000 0.00002 0.00000<br>background w 0.05983 1.00000 0.05983 1.00000<br>foreground w 0.05983 1.00000 1.10996 1.10996	17	-1123.14	0<br>*P*=1	
Branch k<br>$\omega_a = 1$	site class 0 1 2a 2b<br>proportion 0.99999 0.00000 0.00001 0.00000<br>background w 0.05983 1.00000 0.05983 1.00000<br>foreground w 0.05983 1.00000 1.00000 1.00000	16	-1123.14		
**One ratio**	ω =0.0217	8	-2867.18		
**Branch-site Model**					
Branch l	site class 0 1 2a 2b<br>proportion 0.68791 0.00651 0.30272 0.00286<br>background w 0.01800 1.00000 0.01800 1.00000<br>foreground w 0.01800 1.00000 1.00000 1.00000	21	-2860.72	0<br>*P*=1	
Branch l<br>$\omega_a = 1$	site class 0 1 2a 2b<br>proportion 0.68791 0.00651 0.30272 0.00286<br>background w 0.01800 1.00000 0.01800 1.00000<br>foreground w 0.01800 1.00000 1.00000 1.00000	20	-2860.72		
**One ratio**	ω =0.0187	12	-2037.56		
**Branch-site Model**					
Branch m	site class 0 1 2a 2b<br>proportion 0.98245 0.01755 0.00000 0.00000<br>background w 0.01397 1.00000 0.01397 1.00000<br>foreground w 0.01397 1.00000 1.00000 1.00000	15	-2025.19	0<br>*P*=1	
Branch m<br>$\omega_a = 1$	site class 0 1 2a 2b<br>proportion 0.98245 0.01755 0.00000 0.00000<br>background w 0.01397 1.00000 0.01397 1.00000<br>foreground w 0.01397 1.00000 1.00000 1.00000	14	-2025.19		

6. CytB

Model	Parameter Estimated[a]	p	lnL	2⊿lnL[b]<br>*P* value[c]	Positively Selected Sites (BEB Analysis)[d]
**One ratio**	ω =0.0201	14	-1595.84		

**Branch-site Model**					
Branch k	site class 0 1 2a 2b<br>proportion 1.00000 0.00000 0.00000 0.00000<br>background w 0.02008 1.00000 0.02008 1.00000<br>foreground w 0.02008 1.00000 1.06219 1.06219	17	-1595.84	0<br>*P*=1	
Branch k<br>ω_a = 1	site class 0 1 2a 2b<br>proportion 0.99999 0.00000 0.00001 0.00000<br>background w 0.02008 1.00000 0.02008 1.00000<br>foreground w 0.02008 1.00000 1.00000 1.00000	16	-1595.84		
**One ratio**	ω =0.0080	12	-3006.98		
**Branch-site Model**					
Branch m	site class 0 1 2a 2b<br>proportion 0.97835 0.02165 0.00000 0.00000<br>background w 0.00403 1.00000 0.00403 1.00000<br>foreground w 0.00403 1.00000 1.00000 1.00000	15	-2987.97	0<br>*P*=1	
Branch m<br>ω_a = 1	site class 0 1 2a 2b<br>proportion 0.97835 0.02165 0.00000 0.00000<br>background w 0.00403 1.00000 0.00403 1.00000<br>foreground w 0.00403 1.00000 1.00000 1.00000	14	-2987.97		

7. ND1

**Model**	**Parameter Estimated**[a]	**p**	**lnL**	**2⊿lnL**[b]<br>***P* value**[c]	**Positively Selected Sites (BEB Analysis)**[d]
**One ratio**	ω =0.0285	14	-1657.67		
**Branch-site Model**					
Branch k	site class 0 1 2a 2b<br>proportion 0.98136 0.01864 0.00000 0.00000<br>background w 0.00000 1.00000 0.00000 1.00000<br>foreground w 0.00000 1.00000 1.00000 1.00000	17	-1643.17	0<br>*P*=1	
Branch k<br>ω_a = 1	site class 0 1 2a 2b<br>proportion 0.98136 0.01864 0.00000 0.00000<br>background w 0.00000 1.00000 0.00000 1.00000<br>foreground w 0.00000 1.00000 1.00000 1.00000	16	-1643.17		
**One ratio**	ω =0.0154	18	-4132.89		
**Branch-site Model**					
Branch l	site class 0 1 2a 2b<br>proportion 0.98368 0.01632 0.00000 0.00000<br>background w 0.01021 1.00000 0.01021 1.00000<br>foreground w 0.01021 1.00000 7.89625 7.89625	21	-4122.38	0<br>*P*=1	
Branch l<br>ω_a = 1	site class 0 1 2a 2b<br>proportion 0.00000 0.00000 0.98368 0.01632<br>background w 0.01021 1.00000 0.01021 1.00000<br>foreground w 0.01021 1.00000 1.00000 1.00000	20	-4122.38		
**One ratio**	ω =0.0095	12	-2700.62		

**Branch-site Model**					
Branch m	site class 0 1 2a 2b<br>proportion 0.96035 0.03965 0.00000 0.00000<br>background w 0.00343 1.00000 0.00343 1.00000<br>foreground w 0.00343 1.00000 1.00000 1.00000	15	-2686.40	0<br>*P*=1	
Branch m<br>$\omega_a = 1$	site class 0 1 2a 2b<br>proportion 0.96035 0.03965 0.00000 0.00000<br>background w 0.00343 1.00000 0.00343 1.00000<br>foreground w 0.00343 1.00000 1.00000 1.00000	14	-2686.40		

8. ND2

Model	Parameter Estimated[a]	p	lnL	2⊿lnL[b]<br>*P* value[c]	Positively Selected Sites (BEB Analysis)[d]
**One ratio**	ω =0.0547	14	-1784.74		
**Branch-site Model**					
Branch k	site class 0 1 2a 2b<br>proportion 0.94431 0.05569 0.00000 0.00000<br>background w 0.00361 1.00000 0.00361 1.00000<br>foreground w 0.00361 1.00000 1.00000 1.00000	17	-1779.50	0<br>*P*=1	
Branch k<br>$\omega_a = 1$	site class 0 1 2a 2b<br>proportion 0.94431 0.05569 0.00000 0.00000<br>background w 0.00361 1.00000 0.00361 1.00000<br>foreground w 0.00361 1.00000 1.00000 1.00000	16	-1779.50		
**One ratio**	ω =0.0422	18	-4725.73		
**Branch-site Model**					
Branch l	site class 0 1 2a 2b<br>proportion 0.97327 0.02673 0.00000 0.00000<br>background w 0.03424 1.00000 0.03424 1.00000<br>foreground w 0.03424 1.00000 1.00000 1.00000	21	-4704.78	0<br>*P*=1	
Branch l<br>$\omega_a = 1$	site class 0 1 2a 2b<br>proportion 0.97327 0.02673 0.00000 0.00000<br>background w 0.03424 1.00000 0.03424 1.00000<br>foreground w 0.03424 1.00000 1.00000 1.00000	20	-4704.78		
**One ratio**	ω =0.0422	12	-3263.51		
**Branch-site Model**					
Branch m	site class 0 1 2a 2b<br>proportion 0.95245 0.04755 0.00000 0.00000<br>background w 0.02536 1.00000 0.02536 1.00000<br>foreground w 0.02536 1.00000 1.00000 1.00000	15	-3248.10	0<br>*P*=1	
Branch m<br>$\omega_a = 1$	site class 0 1 2a 2b<br>proportion 0.95245 0.04755 0.00000 0.00000<br>background w 0.02536 1.00000 0.02536 1.00000<br>foreground w 0.02536 1.00000 1.00000 1.00000	14	-3248.10		

9. ND3

Model	Parameter Estimated[a]	p	lnL	2⊿lnL[b]<br>*P* value[c]	Positively Selected Sites (BEB Analysis)[d]
**One ratio**	ω =0.0532	14	-501.56		
**Branch-site Model**					
Branch k	site class 0 1 2a 2b<br>proportion 0.88038 0.06967 0.04629 0.00366<br>background w 0.00000 1.00000 0.00000 1.00000<br>foreground w 0.00000 1.00000 1.00000 1.00000	17	-501.35	0<br>*P*=1	
Branch k<br>ω_a = 1	site class 0 1 2a 2b<br>proportion 0.80333 0.06357 0.12334 0.00976<br>background w 0.00000 1.00000 0.00000 1.00000<br>foreground w 0.00000 1.00000 1.00000 1.00000	16	-501.35		
**One ratio**	ω =0.0311	12	-969.26		
**Branch-site Model**					
Branch m	site class 0 1 2a 2b<br>proportion 0.90626 0.09374 0.00000 0.00000<br>background w 0.01411 1.00000 0.01411 1.00000<br>foreground w 0.01411 1.00000 1.00000 1.00000	15	-962.29	0<br>*P*=1	
Branch m<br>ω_a = 1	site class 0 1 2a 2b<br>proportion 0.90626 0.09374 0.00000 0.00000<br>background w 0.01411 1.00000 0.01411 1.00000<br>foreground w 0.01411 1.00000 1.00000 1.00000	14	-962.29		

10. ND4

Model	Parameter Estimated[a]	p	lnL	2⊿lnL[b]<br>*P* value[c]	Positively Selected Sites (BEB Analysis)[d]
**One ratio**	ω =0.0308	14	-2359.75		
**Branch-site Model**					
Branch k	site class 0 1 2a 2b<br>proportion 1.00000 0.00000 0.00000 0.00000<br>background w 0.03082 1.00000 0.03082 1.00000<br>foreground w 0.03082 1.00000 1.00000 1.00000	17	-2359.75	0<br>*P*=1	
Branch k<br>ω_a = 1	site class 0 1 2a 2b<br>proportion 1.00000 0.00000 0.00000 0.00000<br>background w 0.03082 1.00000 0.03082 1.00000<br>foreground w 0.03082 1.00000 1.00000 1.00000	16	-2359.75		
**One ratio**	ω =0.0454	18	-6059.05		
**Branch-site Model**					
Branch l	site class 0 1 2a 2b<br>proportion 0.96914 0.03086 0.00000 0.00000<br>background w 0.03564 1.00000 0.03564 1.00000<br>foreground w 0.03564 1.00000 1.00000 1.00000	21	-6027.58	0<br>*P*=1	
Branch l<br>ω_a = 1	site class 0 1 2a 2b<br>proportion 0.96914 0.03086 0.00000 0.00000	20	-6027.58		

	background w 0.03564 1.00000 0.03564 1.00000<br>foreground w 0.03564 1.00000 1.00000 1.00000				
**One ratio**	ω =0.0276	12	-4043.47		
**Branch-site Model**					
Branch m	site class 0 1 2a 2b<br>proportion 0.94286 0.05714 0.00000 0.00000<br>background w 0.01113 1.00000 0.01113 1.00000<br>foreground w 0.01113 1.00000 1.00000 1.00000	15	-4013.96	0<br>*P*=1	
Branch m<br>ω_a = 1	site class 0 1 2a 2b<br>proportion 0.94286 0.05714 0.00000 0.00000<br>background w 0.01113 1.00000 0.01113 1.00000<br>foreground w 0.01113 1.00000 1.00000 1.00000	14	-4013.96		

11. ND4L

Model	Parameter Estimated[a]	p	lnL	2⊿lnL[b]<br>*P* value[c]	Positively Selected Sites (BEB Analysis)[d]
**One ratio**	ω =0.1641	14	-467.50		
**Branch-site Model**					
Branch k	site class 0 1 2a 2b<br>proportion 0.99999 0.00000 0.00001 0.00000<br>background w 0.16413 1.00000 0.16413 1.00000<br>foreground w 0.16413 1.00000 1.07646 1.07646	17	-467.50	0<br>*P*=1	
Branch k<br>ω_a = 1	site class 0 1 2a 2b<br>proportion 0.99999 0.00000 0.00001 0.00000<br>background w 0.16413 1.00000 0.16413 1.00000<br>foreground w 0.16413 1.00000 1.00000 1.00000	16	-467.50		
**One ratio**	ω =0.0420	18	-1133.98		
**Branch-site Model**					
Branch l	site class 0 1 2a 2b<br>proportion 0.96732 0.03268 0.00000 0.00000<br>background w 0.03169 1.00000 0.03169 1.00000<br>foreground w 0.03169 1.00000 1.00000 1.00000	21	-1130.04	0<br>*P*=1	
Branch l<br>ω_a = 1	site class 0 1 2a 2b<br>proportion 0.96732 0.03268 0.00000 0.00000<br>background w 0.03169 1.00000 0.03169 1.00000<br>foreground w 0.03169 1.00000 1.00000 1.00000	20	-1130.04		
**One ratio**	ω =0.0403	12	-746.41		
**Branch-site Model**					
Branch m	site class 0 1 2a 2b<br>proportion 0.92871 0.07129 0.00000 0.00000<br>background w 0.01814 1.00000 0.01814 1.00000<br>foreground w 0.01814 1.00000 1.00000 1.00000	15	-744.05	0<br>*P*=1	
Branch m<br>ω_a = 1	site class 0 1 2a 2b<br>proportion 0.92870 0.07130 0.00000 0.00000	14	-744.05		

	background w 0.01814 1.00000 0.01814 1.00000<br>foreground w 0.01814 1.00000 1.00000 1.00000				

12. ND5

Model	Parameter Estimated[a]	p	lnL	2⊿lnL[b]<br>*P* value[c]	Positively Selected Sites (BEB Analysis)[d]
**One ratio**	ω =0.0575	14	-3098.61		
**Branch-site Model**					
Branch k	site class 0 1 2a 2b<br>proportion 0.94231 0.05588 0.00171 0.00010<br>background w 0.00196 1.00000 0.00196 1.00000<br>foreground w 0.00196 1.00000 999.00000 999.00000	17	-3089.29	2.98<br>*P*= 0.08	
Branch k<br>ω_a = 1	site class 0 1 2a 2b<br>proportion 0.87186 0.05200 0.07186 0.00429<br>background w 0.00277 1.00000 0.00277 1.00000<br>foreground w 0.00277 1.00000 1.00000 1.00000	16	-3090.78		
**One ratio**	ω =0.0512	18	-7929.81		
**Branch-site Model**					
Branch l	site class 0 1 2a 2b<br>proportion 0.90146 0.04538 0.05061 0.00255<br>background w 0.03556 1.00000 0.03556 1.00000<br>foreground w 0.03556 1.00000 1.00000 1.00000	21	-7868.87	0<br>*P*=1	
Branch l<br>ω_a = 1	site class 0 1 2a 2b<br>proportion 0.90146 0.04538 0.05061 0.00255<br>background w 0.03556 1.00000 0.03556 1.00000<br>foreground w 0.03556 1.00000 1.00000 1.00000	20	-7868.87		
**One ratio**	ω =0.0280	12	-5283.39		
**Branch-site Model**					
Branch m	site class 0 1 2a 2b<br>proportion 0.92971 0.05045 0.01882 0.00102<br>background w 0.01198 1.00000 0.01198 1.00000<br>foreground w 0.01198 1.00000 1.00000 1.00000	15	-5251.13	1.62<br>*P*=0.203	
Branch m<br>ω_a = 1	site class 0 1 2a 2b<br>proportion 0.94837 0.05163 0.00000 0.00000<br>background w 0.01309 1.00000 0.01309 1.00000<br>foreground w 0.01309 1.00000 1.00000 1.00000	14	-5251.94		

13. ND6

Model	Parameter Estimated[a]	p	lnL	2⊿lnL[b]<br>*P* value[c]	Positively Selected Sites (BEB Analysis)[d]
**One ratio**	ω =0.0539	14	-830.72		
**Branch-site Model**					
Branch k	site class 0 1 2a 2b<br>proportion 1.00000 0.00000 0.00000 0.00000	17	-830.72	0.18<br>*P*=0.67	

	background w 0.05395 1.00000 0.05395 1.00000<br>foreground w 0.05395 1.00000 1.00000 1.00000				
Branch k<br>ω_a = 1	site class 0 1 2a 2b<br>proportion 1.00000 0.00000 0.00000 0.00000<br>background w 0.05392 1.00000 0.05392 1.00000<br>foreground w 0.05392 1.00000 1.00000 1.00000	16	-830.81		
**One ratio**	ω =0.0412	18	-2381.69		
**Branch-site Model**					
Branch l	site class 0 1 2a 2b<br>proportion 0.93045 0.06955 0.00000 0.00000<br>background w 0.03088 1.00000 0.03088 1.00000<br>foreground w 0.03088 1.00000 1.00000 1.00000	21	-2368.74	0<br>*P*=1	
Branch l<br>ω_a = 1	site class 0 1 2a 2b<br>proportion 0.93045 0.06955 0.00000 0.00000<br>background w 0.03088 1.00000 0.03088 1.00000<br>foreground w 0.03088 1.00000 1.00000 1.00000	20	-2368.74		
**One ratio**	ω =0.0226	12	-1490.92		
**Branch-site Model**					
Branch m	site class 0 1 2a 2b<br>proportion 0.99001 0.00999 0.00000 0.00000<br>background w 0.01995 1.00000 0.01995 1.00000<br>foreground w 0.01995 1.00000 1.00000 1.00000	15	-1489.54	0<br>*P*=1	
Branch m<br>ω_a = 1	site class 0 1 2a 2b<br>proportion 0.99001 0.00999 0.00000 0.00000<br>background w 0.01995 1.00000 0.01995 1.00000<br>foreground w 0.01995 1.00000 1.00000 1.00000	14	-1489.54		
**One ratio**	ω =0.0214	13	-1668.45		
**Branch-site Model**					
Branch n	site class 0 1 2a 2b<br>proportion 0.98256 0.01744 0.00000 0.00000<br>background w 0.01710 1.00000 0.01710 1.00000<br>foreground w 0.01710 1.00000 1.00000 1.00000	16	-1663.54	0<br>*P*=1	
Branch n<br>ω_a = 1	site class 0 1 2a 2b<br>proportion 0.98256 0.01744 0.00000 0.00000<br>background w 0.01710 1.00000 0.01710 1.00000<br>foreground w 0.01710 1.00000 1.00000 1.00000	15	-1663.54		

[a] The proportion of sites estimated to have ω

[b] Twice the difference in log-likelihood values was calculated following a chi-squared ($\chi 2$)

[c] Posterior probability>0.99 of having ω>1

[d] The residues were identified by Bayes Empiriacal Bayes analysis

Supplementary Table 7: Selective pressure analyses for the 13 genes in Carangimorpha.

1. ATP6

**Model**	**Parameter Estimated**[a]	**p**	**lnL**	**2⊿lnL**[b] ***P* value**[c]	**Positively Selected Sites (BEB Analysis)**[d]
**One ratio**	ω =0.0130	13	-2265.00		
**Branch-site Model**					
Branch n	site class 0 1 2a 2b<br>proportion 1.00000 0.00000 0.00000 0.00000<br>background w 0.01295 1.00000 0.01295 1.00000<br>foreground w 0.01295 1.00000 1.00000 1.00000	16	-2265.00	0<br>*P*=1	
Branch n $\omega_a = 1$	site class 0 1 2a 2b<br>proportion 0.05766 0.00000 0.94234 0.00000<br>background w 0.01295 1.00000 0.01295 1.00000<br>foreground w 0.01295 1.00000 1.00000 1.00000	15	-2265.00		

2. ATP8

**Model**	**Parameter Estimated**[a]	**p**	**lnL**	**2⊿lnL**[b] ***P* value**[c]	**Positively Selected Sites (BEB Analysis)**[d]
**One ratio**	ω =0.0623	13	-486.38		
**Branch-site Model**					
Branch n	site class 0 1 2a 2b<br>proportion 0.73112 0.02710 0.23314 0.00864<br>background w 0.03657 1.00000 0.03657 1.00000<br>foreground w 0.03657 1.00000 1.00000 1.00000	16	-480.30	0<br>*P*=1	
Branch n $\omega_a = 1$	site class 0 1 2a 2b<br>proportion 0.73112 0.02710 0.23314 0.00864<br>background w 0.03657 1.00000 0.03657 1.00000<br>foreground w 0.03657 1.00000 1.00000 1.00000	15	-480.30		

3. CoxI

**Model**	**Parameter Estimated**[a]	**p**	**lnL**	**2⊿lnL**[b] ***P* value**[c]	**Positively Selected Sites (BEB Analysis)**[d]
**One ratio**	ω =0.0125	13	-4351.99		
**Branch-site Model**					
Branch n	site class 0 1 2a 2b<br>proportion 0.99534 0.00466 0.00000 0.00000<br>background w 0.01079 1.00000 0.01079 1.00000<br>foreground w 0.01079 1.00000 1.00000 1.00000	16	-4349.90	0<br>*P*=1	
Branch n $\omega_a = 1$	site class 0 1 2a 2b<br>proportion 0.99534 0.00466 0.00000 0.00000<br>background w 0.01079 1.00000 0.01079 1.00000<br>foreground w 0.01079 1.00000 1.00000 1.00000	15	-4349.90		

4. CoxII

Model	Parameter Estimated[a]	p	lnL	2⊿lnL[b]<br>*P* value[c]	Positively Selected Sites (BEB Analysis)[d]
**One ratio**	ω =0.0162	13	-1837.11		
**Branch-site Model**					
Branch n	site class 0 1 2a 2b<br>proportion 1.00000 0.00000 0.00000 0.00000<br>background w 0.01618 1.00000 0.01618 1.00000<br>foreground w 0.01618 1.00000 1.00000 1.00000	16	-1837.11	0<br>*P*=1	
Branch n<br>ω_a = 1	site class 0 1 2a 2b<br>proportion 1.00000 0.00000 0.00000 0.00000<br>background w 0.01618 1.00000 0.01618 1.00000<br>foreground w 0.01618 1.00000 1.00000 1.00000	15	-1837.11		

5. CoxIII

Model	Parameter Estimated[a]	p	lnL	2⊿lnL[b]<br>*P* value[c]	Positively Selected Sites (BEB Analysis)[d]
**One ratio**	ω =0.0145	13	-2303.52		
**Branch-site Model**					
Branch n	site class 0 1 2a 2b<br>proportion 0.93074 0.00575 0.06312 0.00039<br>background w 0.01265 1.00000 0.01265 1.00000<br>foreground w 0.01265 1.00000 1.00000 1.00000	16	-2301.92	0<br>*P*=1	
Branch n<br>ω_a = 1	site class 0 1 2a 2b<br>proportion 0.93074 0.00575 0.06312 0.00039<br>background w 0.01265 1.00000 0.01265 1.00000<br>foreground w 0.01265 1.00000 1.00000 1.00000	15	-2301.92		

6. CytB

Model	Parameter Estimated[a]	p	lnL	2⊿lnL[b]<br>*P* value[c]	Positively Selected Sites (BEB Analysis)[d]
**One ratio**	ω =0.0119	13	-3521.32		
**Branch-site Model**					
Branch n	site class 0 1 2a 2b<br>proportion 0.95138 0.00890 0.03935 0.00037<br>background w 0.00946 1.00000 0.00946 1.00000<br>foreground w 0.00946 1.00000 1.00000 1.00000	16	-3514.44	1.98<br>*P*=0.159	
Branch n<br>ω_a = 1	site class 0 1 2a 2b<br>proportion 0.00000 0.00000 0.99120 0.00880<br>background w 0.00918 1.00000 0.00918 1.00000<br>foreground w 0.00918 1.00000 1.00000 1.00000	15	-3515.43		

7. ND1

Model	Parameter Estimated[a]	p	lnL	2⊿lnL[b]<br>*P* value[c]	Positively Selected Sites (BEB Analysis)[d]

**One ratio**	ω =0.0096	13	-3011.12		
**Branch-site Model**					
Branch n	site class 0 1 2a 2b<br>proportion 0.00000 0.00000 0.99061 0.00939<br>background w 0.00641 1.00000 0.00641 1.00000<br>foreground w 0.00641 1.00000 114.71367 114.71367	16	-3002.17	0.14<br>*P*= 0.71	
Branch n<br>ω_a = 1	site class 0 1 2a 2b<br>proportion 0.00000 0.00000 0.99060 0.00940<br>background w 0.00642 1.00000 0.00642 1.00000<br>foreground w 0.00642 1.00000 1.00000 1.00000	15	-3002.24		

8. ND2

Model	Parameter Estimated[a]	p	lnL	2⊿lnL[b]<br>*P* value[c]	Positively Selected Sites (BEB Analysis)[d]
**One ratio**	ω =0.0274	13	-3617.49		
**Branch-site Model**					
Branch n	site class 0 1 2a 2b<br>proportion 0.99101 0.00899 0.00000 0.00000<br>background w 0.02498 1.00000 0.02498 1.00000<br>foreground w 0.02498 1.00000 1.00000 1.00000	16	-3614.35	0<br>*P*=1	
Branch n<br>ω_a = 1	site class 0 1 2a 2b<br>proportion 0.99101 0.00899 0.00000 0.00000<br>background w 0.02498 1.00000 0.02498 1.00000<br>foreground w 0.02498 1.00000 1.00000 1.00000	15	-3614.35		

9. ND3

Model	Parameter Estimated[a]	p	lnL	2⊿lnL[b]<br>*P* value[c]	Positively Selected Sites (BEB Analysis)[d]
**One ratio**	ω =0.0131	13	-1058.21		
**Branch-site Model**					
Branch n	site class 0 1 2a 2b<br>proportion 0.97444 0.02556 0.00000 0.00000<br>background w 0.00618 1.00000 0.00618 1.00000<br>foreground w 0.00618 1.00000 1.00000 1.00000	16	-1046.11	0<br>*P*=1	
Branch n<br>ω_a = 1	site class 0 1 2a 2b<br>proportion 0.97444 0.02556 0.00000 0.00000<br>background w 0.00618 1.00000 0.00618 1.00000<br>foreground w 0.00618 1.00000 1.00000 1.00000	15	-1046.11		

10. ND4

Model	Parameter Estimated[a]	p	lnL	2⊿lnL[b]<br>*P* value[c]	Positively Selected Sites (BEB Analysis)[d]
**One ratio**	ω =0.0181	13	-4424.05		
**Branch-site Model**					
Branch n	site class 0 1 2a 2b	16	-4417.58	0	

	proportion 0.97940 0.00719 0.01331 0.00010<br>background w 0.01536 1.00000 0.01536 1.00000<br>foreground w 0.01536 1.00000 1.00000 1.00000			*P*=1	
Branch n<br>ω_a = 1	site class 0 1 2a 2b<br>proportion 0.97940 0.00719 0.01331 0.00010<br>background w 0.01536 1.00000 0.01536 1.00000<br>foreground w 0.01536 1.00000 1.00000 1.00000	15	-4417.58		

11. ND4L

Model	Parameter Estimated[a]	p	lnL	2⊿lnL[b]<br>*P* value[c]	Positively Selected Sites (BEB Analysis)[d]
**One ratio**	ω =0.0113	13	-861.36		
**Branch-site Model**					
Branch n	site class 0 1 2a 2b<br>proportion 0.71293 0.00000 0.28707 0.00000<br>background w 0.00965 1.00000 0.00965 1.00000<br>foreground w 0.00965 1.00000 1.00000 1.00000	16	-859.52	0<br>*P*=1	
Branch n<br>ω_a = 1	site class 0 1 2a 2b<br>proportion 0.71293 0.00000 0.28707 0.00000<br>background w 0.00965 1.00000 0.00965 1.00000<br>foreground w 0.00965 1.00000 1.00000 1.00000	15	-859.52		

12. ND5

Model	Parameter Estimated[a]	p	lnL	2⊿lnL[b]<br>*P* value[c]	Positively Selected Sites (BEB Analysis)[d]
**One ratio**	ω =0.0415	13	0.0415		
**Branch-site Model**					
Branch n	site class 0 1 2a 2b<br>proportion 0.96372 0.02366 0.01232 0.00030<br>background w 0.03245 1.00000 0.03245 1.00000<br>foreground w 0.03245 1.00000 1.00000 1.00000	16	-6186.16	0<br>*P*=1	
Branch n<br>ω_a = 1	site class 0 1 2a 2b<br>proportion 0.96372 0.02366 0.01232 0.00030<br>background w 0.03245 1.00000 0.03245 1.00000<br>foreground w 0.03245 1.00000 1.00000 1.00000	15	-6186.16		

13. ND6

Model	Parameter Estimated[a]	p	lnL	2⊿lnL[b]<br>*P* value[c]	Positively Selected Sites (BEB Analysis)[d]
**One ratio**	ω =0.0214	13	-1668.45		
**Branch-site Model**					
Branch n	site class 0 1 2a 2b<br>proportion 0.98256 0.01744 0.00000 0.00000<br>background w 0.01710 1.00000 0.01710 1.00000<br>foreground w 0.01710 1.00000 1.00000 1.00000	16	-1663.54	0<br>*P*=1	

Branch n $\omega_a = 1$	site class 0 1 2a 2b<br>proportion 0.98256 0.01744 0.00000 0.00000<br>background w 0.01710 1.00000 0.01710 1.00000<br>foreground w 0.01710 1.00000 1.00000 1.00000	15	-1663.54		

[a] The proportion of sites estimated to have ω

[b] Twice the difference in log-likelihood values was calculated following a chi-squared ($\chi 2$)

[c] Posterior probability>0.99 of having ω>1

[d] The residues were identified by Bayes Empiriacal Bayes analysis

Supplementary Table 8: Selective pressure analyses for the 13 genes in Scombriformes.

1. ATP6

Model	Parameter Estimated[a]	p	lnL	2⊿lnL[b]<br>*P* value[c]	Positively Selected Sites (BEB Analysis)[d]
**One ratio**	ω =0.0188	12	-1640.17		
**Branch-site Model**					
Branch o	site class 0 1 2a 2b<br>proportion 0.96986 0.00385 0.02619 0.00010<br>background w 0.01501 1.00000 0.01501 1.00000<br>foreground w 0.01501 1.00000 1.00000 1.00000	15	-1639.77	0<br>*P*=1	
Branch o<br>ω_a = 1	site class 0 1 2a 2b<br>proportion 0.96986 0.00385 0.02619 0.00010<br>background w 0.01501 1.00000 0.01501 1.00000<br>foreground w 0.01501 1.00000 1.00000 1.00000	14	-1639.77		

2. ATP8

Model	Parameter Estimated[a]	p	lnL	2⊿lnL[b]<br>*P* value[c]	Positively Selected Sites (BEB Analysis)[d]
**One ratio**	ω =0.1340	12	-351.88		
**Branch-site Model**					
Branch o	site class 0 1 2a 2b<br>proportion 1.00000 0.00000 0.00000 0.00000<br>background w 0.13403 1.00000 0.13403 1.00000<br>foreground w 0.13403 1.00000 1.00000 1.00000	15	-351.88	0<br>*P*=1	
Branch o<br>ω_a = 1	site class 0 1 2a 2b<br>proportion 1.00000 0.00000 0.00000 0.00000<br>background w 0.13403 1.00000 0.13403 1.00000<br>foreground w 0.13403 1.00000 1.00000 1.00000	14	-351.88		

3. CoxI

Model	Parameter Estimated[a]	p	lnL	2⊿lnL[b]<br>*P* value[c]	Positively Selected Sites (BEB Analysis)[d]
**One ratio**	ω =0.0386	12	-3520.72		
**Branch-site Model**					
Branch o	site class 0 1 2a 2b<br>proportion 0.97897 0.02103 0.00000 0.00000<br>background w 0.02753 1.00000 0.02753 1.00000<br>foreground w 0.02753 1.00000 1.00000 1.00000	15	-3516.60	0<br>*P*=1	
Branch o<br>ω_a = 1	site class 0 1 2a 2b<br>proportion 0.97897 0.02103 0.00000 0.00000<br>background w 0.02753 1.00000 0.02753 1.00000<br>foreground w 0.02753 1.00000 1.00000 1.00000	14	-3516.60		

4. CoxII

Model	Parameter Estimated[a]	p	lnL	2⊿lnL[b]<br>*P* value[c]	Positively Selected Sites (BEB Analysis)[d]
**One ratio**	ω =0.0262	12	-1457.63		
**Branch-site Model**					
Branch o	site class 0 1 2a 2b<br>proportion 0.98340 0.01660 0.00000 0.00000<br>background w 0.01826 1.00000 0.01826 1.00000<br>foreground w 0.01826 1.00000 1.00000 1.00000	15	-1456.10	0<br>*P*=1	
Branch o<br>$\omega_a = 1$	site class 0 1 2a 2b<br>proportion 0.98340 0.01660 0.00000 0.00000<br>background w 0.01826 1.00000 0.01826 1.00000<br>foreground w 0.01826 1.00000 1.00000 1.00000	14	-1456.10		

5. CoxIII

Model	Parameter Estimated[a]	p	lnL	2⊿lnL[b]<br>*P* value[c]	Positively Selected Sites (BEB Analysis)[d]
**One ratio**	ω =0.0236	12	-1687.20		
**Branch-site Model**					
Branch o	site class 0 1 2a 2b<br>proportion 0.93838 0.03442 0.02623 0.00096<br>background w 0.00269 1.00000 0.00269 1.00000<br>foreground w 0.00269 1.00000 1.00000 1.00000	15	-1677.34	0<br>*P*=1	
Branch o<br>$\omega_a = 1$	site class 0 1 2a 2b<br>proportion 0.93838 0.03442 0.02623 0.00096<br>background w 0.00269 1.00000 0.00269 1.00000<br>foreground w 0.00269 1.00000 1.00000 1.00000	14	-1677.34		

6. CytB

Model	Parameter Estimated[a]	p	lnL	2⊿lnL[b]<br>*P* value[c]	Positively Selected Sites (BEB Analysis)[d]
**One ratio**	ω =0.0278	12	-2738.25		
**Branch-site Model**					
Branch o	site class 0 1 2a 2b<br>proportion 0.94437 0.01831 0.03661 0.00071<br>background w 0.01597 1.00000 0.01597 1.00000<br>foreground w 0.01597 1.00000 1.23020 1.23020	15	-2732.71	0.02<br>*P*=0.89	
Branch o<br>$\omega_a = 1$	site class 0 1 2a 2b<br>proportion 0.93919 0.01831 0.04169 0.00081<br>background w 0.01582 1.00000 0.01582 1.00000<br>foreground w 0.01582 1.00000 1.00000 1.00000	14	-2732.72		

7. ND1

Model	Parameter Estimated[a]	p	lnL	2⊿lnL[b]<br>*P* value[c]	Positively Selected Sites (BEB Analysis)[d]

**One ratio**	ω =0.0150	12	-2382.49		
**Branch-site Model**					
Branch o	site class 0 1 2a 2b<br>proportion 0.97522 0.00983 0.01479 0.00015<br>background w 0.00926 1.00000 0.00926 1.00000<br>foreground w 0.00926 1.00000 2.86228 2.86228	15	-2376.94	0.36<br>*P*=0.55	
Branch o<br>ω_a = 1	site class 0 1 2a 2b<br>proportion 0.96385 0.00949 0.02640 0.00026<br>background w 0.00909 1.00000 0.00909 1.00000<br>foreground w 0.00909 1.00000 1.00000 1.00000	14	-2377.12		

8. ND2

**Model**	**Parameter Estimated[a]**	**p**	**lnL**	**2⊿lnL[b]**<br>***P* value[c]**	**Positively Selected Sites (BEB Analysis)[d]**
**One ratio**	ω =0.0193	12	-2657.02		
**Branch-site Model**					
Branch o	site class 0 1 2a 2b<br>proportion 0.98249 0.01751 0.00000 0.00000<br>background w 0.01336 1.00000 0.01336 1.00000<br>foreground w 0.01336 1.00000 1.00000 1.00000	15	-2653.64	0<br>*P*=1	
Branch o<br>ω_a = 1	site class 0 1 2a 2b<br>proportion 0.98249 0.01751 0.00000 0.00000<br>background w 0.01336 1.00000 0.01336 1.00000<br>foreground w 0.01336 1.00000 1.00000 1.00000	14	-2653.64		

9. ND3

**Model**	**Parameter Estimated[a]**	**p**	**lnL**	**2⊿lnL[b]**<br>***P* value[c]**	**Positively Selected Sites (BEB Analysis)[d]**
**One ratio**	ω =0.0219	12	-763.56		
**Branch-site Model**					
Branch o	site class 0 1 2a 2b<br>proportion 0.92713 0.03976 0.03175 0.00136<br>background w 0.00543 1.00000 0.00543 1.00000<br>foreground w 0.00543 1.00000 999.00000 999.00000	15	-758.41	0.56<br>*P*= 0.45	
Branch o<br>ω_a = 1	site class 0 1 2a 2b<br>proportion 0.41343 0.01788 0.54511 0.02357<br>background w 0.00556 1.00000 0.00556 1.00000<br>foreground w 0.00556 1.00000 1.00000 1.00000	14	-758.69		

10. ND4

**Model**	**Parameter Estimated[a]**	**p**	**lnL**	**2⊿lnL[b]**<br>***P* value[c]**	**Positively Selected Sites (BEB Analysis)[d]**
**One ratio**	ω =0.0181	12	-3481.45		
**Branch-site Model**					
Branch o	site class 0 1 2a 2b	15	-3479.76	0	

	proportion 0.98445 0.01555 0.00000 0.00000<br>background w 0.01361 1.00000 0.01361 1.00000<br>foreground w 0.01361 1.00000 1.00000 1.00000			*P*=1	
Branch o<br>ω_a = 1	site class 0 1 2a 2b<br>proportion 0.98445 0.01555 0.00000 0.00000<br>background w 0.01361 1.00000 0.01361 1.00000<br>foreground w 0.01361 1.00000 1.00000 1.00000	14	-3479.76		

11. ND4L

Model	Parameter Estimated[a]	p	lnL	2⊿lnL[b]<br>*P* value[c]	Positively Selected Sites (BEB Analysis)[d]
**One ratio**	ω =0.0067	12	-630.30		
**Branch-site Model**					
Branch o	site class 0 1 2a 2b<br>proportion 1.00000 0.00000 0.00000 0.00000<br>background w 0.00669 1.00000 0.00669 1.00000<br>foreground w 0.00669 1.00000 1.00000 1.00000	15	-630.30	0<br>*P*=1	
Branch o<br>ω_a = 1	site class 0 1 2a 2b<br>proportion 1.00000 0.00000 0.00000 0.00000<br>background w 0.00669 1.00000 0.00669 1.00000<br>foreground w 0.00669 1.00000 1.00000 1.00000	14	-630.30		

12. ND5

Model	Parameter Estimated[a]	p	lnL	2⊿lnL[b]<br>*P* value[c]	Positively Selected Sites (BEB Analysis)[d]
**One ratio**	ω =0.0256	12	-4397.81		
**Branch-site Model**					
Branch o	site class 0 1 2a 2b<br>proportion 0.99403 0.00457 0.00140 0.00001<br>background w 0.02380 1.00000 0.02380 1.00000<br>foreground w 0.02380 1.00000 1.00000 1.00000	15	-4397.55	0<br>*P*=1	
Branch o<br>ω_a = 1	site class 0 1 2a 2b<br>proportion 0.99403 0.00457 0.00140 0.00001<br>background w 0.02380 1.00000 0.02380 1.00000<br>foreground w 0.02380 1.00000 1.00000 1.00000	14	-4397.55		

13. ND6

Model	Parameter Estimated[a]	p	lnL	2⊿lnL[b]<br>*P* value[c]	Positively Selected Sites (BEB Analysis)[d]
**One ratio**	ω =0.0202	12	-1221.22		
**Branch-site Model**					
Branch o	site class 0 1 2a 2b<br>proportion 0.96061 0.03939 0.00000 0.00000<br>background w 0.00722 1.00000 0.00722 1.00000<br>foreground w 0.00722 1.00000 1.00000 1.00000	15	-1214.36	0<br>*P*=1	

Branch o<br>ω_a = 1	site class 0 1 2a 2b<br>proportion 0.96061 0.03939 0.00000 0.00000<br>background w 0.00722 1.00000 0.00722 1.00000<br>foreground w 0.00722 1.00000 1.00000 1.00000	14	-1214.36		

[a] The proportion of sites estimated to have ω

[b] Twice the difference in log-likelihood values was calculated following a chi-squared ($\chi 2$)

[c] Posterior probability>0.99 of having ω>1

[d] The residues were identified by Bayes Empiriacal Bayes analysis

Supplementary Table 9: Selective pressure analyses for the 13 genes in Pleuronectiformes.

1. ATP6

Model	Parameter Estimated[a]	p	lnL	2⊿lnL[b]<br>*P* value[c]	Positively Selected Sites (BEB Analysis)[d]
**One ratio**	ω =0.0634	27	-5073.41		
**Branch-site Model**					
Branch p	site class 0 1 2a 2b<br>proportion 0.90286 0.09714 0.00000 0.00000<br>background w 0.04468 1.00000 0.04468 1.00000<br>foreground w 0.04468 1.00000 1.00000 1.00000	30	-5009.93	0.92<br>*P*=0.34	
Branch p<br>ω_a = 1	site class 0 1 2a 2b<br>proportion 0.66387 0.07140 0.23902 0.02571<br>background w 0.04449 1.00000 0.04449 1.00000<br>foreground w 0.04449 1.00000 1.00000 1.00000	29	-5010.39		

2. ATP8

Model	Parameter Estimated[a]	p	lnL	2⊿lnL[b]<br>*P* value[c]	Positively Selected Sites (BEB Analysis)[d]
**One ratio**	ω =0.0977	27	-1058.19		
**Branch-site Model**					
Branch p	site class 0 1 2a 2b<br>proportion 1.00000 0.00000 0.00000 0.00000<br>background w 0.09768 1.00000 0.09768 1.00000<br>foreground w 0.09768 1.00000 1.00000 1.00000	30	-1058.19	0<br>*P*=1	
Branch p<br>ω_a = 1	site class 0 1 2a 2b<br>proportion 1.00000 0.00000 0.00000 0.00000<br>background w 0.09768 1.00000 0.09768 1.00000<br>foreground w 0.09768 1.00000 1.00000 1.00000	29	-1058.19		

3. CoxI

Model	Parameter Estimated[a]	p	lnL	2⊿lnL[b]<br>*P* value[c]	Positively Selected Sites (BEB Analysis)[d]
**One ratio**	ω =0.0246	27	-8419.00		
**Branch-site Model**					
Branch p	site class 0 1 2a 2b<br>proportion 0.97932 0.02068 0.00000 0.00000<br>background w 0.01991 1.00000 0.01991 1.00000<br>foreground w 0.01991 1.00000 1.00000 1.00000	30	-8389.59	0<br>*P*=1	
Branch p<br>ω_a = 1	site class 0 1 2a 2b<br>proportion 0.97933 0.02067 0.00000 0.00000<br>background w 0.01991 1.00000 0.01991 1.00000<br>foreground w 0.01991 1.00000 1.00000 1.00000	29	-8389.59		

4. CoxII

Model	Parameter Estimated[a]	p	lnL	2⊿lnL[b]<br>*P* value[c]	Positively Selected Sites (BEB Analysis)[d]
**One ratio**	ω =0.0367	27	-4022.46		
**Branch-site Model**					
Branch p	site class 0 1 2a 2b<br>proportion 0.98305 0.01695 0.00000 0.00000<br>background w 0.03263 1.00000 0.03263 1.00000<br>foreground w 0.03263 1.00000 1.00000 1.00000	30	-4011.14	0<br>*P*=1	
Branch p<br>ω_a = 1	site class 0 1 2a 2b<br>proportion 0.98305 0.01695 0.00000 0.00000<br>background w 0.03264 1.00000 0.03264 1.00000<br>foreground w 0.03264 1.00000 1.00000 1.00000	29	-4011.14		

5. CoxIII

Model	Parameter Estimated[a]	p	lnL	2⊿lnL[b]<br>*P* value[c]	Positively Selected Sites (BEB Analysis)[d]
**One ratio**	ω =0.0273	27	-4463.92		
**Branch-site Model**					
Branch p	site class 0 1 2a 2b<br>proportion 0.87028 0.02485 0.10196 0.00291<br>background w 0.02244 1.00000 0.02244 1.00000<br>foreground w 0.02244 1.00000 1.00000 1.00000	30	-4450.31	0<br>*P*=1	
Branch p<br>ω_a = 1	site class 0 1 2a 2b<br>proportion 0.87028 0.02485 0.10196 0.00291<br>background w 0.02244 1.00000 0.02244 1.00000<br>foreground w 0.02244 1.00000 1.00000 1.00000	29	-4450.31		

6. CytB

Model	Parameter Estimated[a]	p	lnL	2⊿lnL[b]<br>*P* value[c]	Positively Selected Sites (BEB Analysis)[d]
**One ratio**	ω =0.0279	27	-7067.21		
**Branch-site Model**					
Branch p	site class 0 1 2a 2b<br>proportion 0.96887 0.03113 0.00000 0.00000<br>background w 0.02290 1.00000 0.02290 1.00000<br>foreground w 0.02290 1.00000 1.00000 1.00000	30	-7036.14	0<br>*P*=1	
Branch p<br>ω_a = 1	site class 0 1 2a 2b<br>proportion 0.96887 0.03113 0.00000 0.00000<br>background w 0.02290 1.00000 0.02290 1.00000<br>foreground w 0.02290 1.00000 1.00000 1.00000	29	-7036.14		

7. ND1

Model	Parameter Estimated[a]	p	lnL	2⊿lnL[b]<br>*P* value[c]	Positively Selected Sites

					(BEB Analysis)[d]
**One ratio**	ω =0.0262	27	-6342.83		
**Branch-site Model**					
Branch p	site class 0 1 2a 2b<br>proportion 1.00000 0.00000 0.00000 0.00000<br>background w 0.02622 1.00000 0.02622 1.00000<br>foreground w 0.02622 1.00000 1.00000 1.00000	30	-6342.83	0<br>*P*=1	
Branch p<br>$\omega_a = 1$	site class 0 1 2a 2b<br>proportion 1.00000 0.00000 0.00000 0.00000<br>background w 0.02622 1.00000 0.02622 1.00000<br>foreground w 0.02622 1.00000 1.00000 1.00000	29	-6342.83		

8. ND2

Model	Parameter Estimated[a]	p	lnL	2⊿lnL[b]<br>*P* value[c]	Positively Selected Sites (BEB Analysis)[d]
**One ratio**	ω =0.0472	27	-8012.40		
**Branch-site Model**					
Branch p	site class 0 1 2a 2b<br>proportion 0.96205 0.03460 0.00324 0.00012<br>background w 0.04046 1.00000 0.04046 1.00000<br>foreground w 0.04046 1.00000 999.00000 999.00000	30	-7980.51	**5.64**<br>***P*=0.018**	201 T 0.983
Branch p<br>$\omega_a = 1$	site class 0 1 2a 2b<br>proportion 0.91777 0.03297 0.04755 0.00171<br>background w 0.04023 1.00000 0.04023 1.00000<br>foreground w 0.04023 1.00000 1.00000 1.00000	29	-7983.33		

9. ND3

Model	Parameter Estimated[a]	p	lnL	2⊿lnL[b]<br>*P* value[c]	Positively Selected Sites (BEB Analysis)[d]
**One ratio**	ω =0.0378	27	-2393.11		
**Branch-site Model**					
Branch p	site class 0 1 2a 2b<br>proportion 0.73426 0.08469 0.16233 0.01872<br>background w 0.02005 1.00000 0.02005 1.00000<br>foreground w 0.02005 1.00000 1.00000 1.00000	30	-2356.86	0<br>*P*=1	
Branch p<br>$\omega_a = 1$	site class 0 1 2a 2b<br>proportion 0.56297 0.06493 0.33362 0.03848<br>background w 0.02005 1.00000 0.02005 1.00000<br>foreground w 0.02005 1.00000 1.00000 1.00000	29	-2356.86		

10. ND4

Model	Parameter Estimated[a]	p	lnL	2⊿lnL[b]<br>*P* value[c]	Positively Selected Sites (BEB Analysis)[d]
**One ratio**	ω =0.0380	27	-9249.77		
**Branch-site Model**					

Branch p	site class 0 1 2a 2b<br>proportion 0.96672 0.03328 0.00000 0.00000<br>background w 0.03079 1.00000 0.03079 1.00000<br>foreground w 0.03079 1.00000 1.00000 1.00000	30	-9202.60	0<br>*P*=1	
Branch p<br>$\omega_a = 1$	site class 0 1 2a 2b<br>proportion 0.96672 0.03328 0.00000 0.00000<br>background w 0.03079 1.00000 0.03079 1.00000<br>foreground w 0.03079 1.00000 1.00000 1.00000	29	-9202.60		

11. ND4L

Model	Parameter Estimated[a]	p	lnL	2⊿lnL[b]<br>*P* value[c]	Positively Selected Sites (BEB Analysis)[d]
**One ratio**	ω =0.0322	27	-1875.30		
**Branch-site Model**					
Branch p	site class 0 1 2a 2b<br>proportion 0.95965 0.04035 0.00000 0.00000<br>background w 0.02718 1.00000 0.02718 1.00000<br>foreground w 0.02718 1.00000 1.00000 1.00000	30	-1872.73	0<br>*P*=1	
Branch p<br>$\omega_a = 1$	site class 0 1 2a 2b<br>proportion 0.95965 0.04035 0.00000 0.00000<br>background w 0.02718 1.00000 0.02718 1.00000<br>foreground w 0.02718 1.00000 1.00000 1.00000	29	-1872.73		

12. ND5

Model	Parameter Estimated[a]	p	lnL	2⊿lnL[b]<br>*P* value[c]	Positively Selected Sites (BEB Analysis)[d]
**One ratio**	ω =0.0617	27	-13282.93		
**Branch-site Model**					
Branch p	site class 0 1 2a 2b<br>proportion 0.90890 0.09110 0.00000 0.00000<br>background w 0.04193 1.00000 0.04193 1.00000<br>foreground w 0.04193 1.00000 1.00000 1.00000	30	-13117.04	3.74<br>*P*=0.053	
Branch p<br>$\omega_a = 1$	site class 0 1 2a 2b<br>proportion 0.03986 0.00395 0.86991 0.08628<br>background w 0.04117 1.00000 0.04117 1.00000<br>foreground w 0.04117 1.00000 1.00000 1.00000	29	-13115.17		

13. ND6

Model	Parameter Estimated[a]	p	lnL	2⊿lnL[b]<br>*P* value[c]	Positively Selected Sites (BEB Analysis)[d]
**One ratio**	ω =0.0489	27	-3843.31		
**Branch-site Model**					
Branch p	site class 0 1 2a 2b<br>proportion 0.00000 0.00000 0.93357 0.06643<br>background w 0.03612 1.00000 0.03612 1.00000	30	-3818.49	0<br>*P*=1	

	foreground w 0.03612 1.00000 1.00000 1.00000				
Branch p<br>$\omega_a = 1$	site class 0 1 2a 2b<br>proportion 0.00000 0.00000 0.93357 0.06643<br>background w 0.03612 1.00000 0.03612 1.00000<br>foreground w 0.03612 1.00000 1.00000 1.00000	29	-3818.49		

[a] The proportion of sites estimated to have ω

[b] Twice the difference in log-likelihood values was calculated following a chi-squared ($\chi 2$)

[c] Posterior probability>0.99 of having ω>1

[d] The residues were identified by Bayes Empiriacal Bayes analysis

Supplementary Table 10: Selective pressure analyses for the 13 genes in Scorpaeniformes.

1. ATP6

**Model**	**Parameter Estimated**[a]	**p**	**lnL**	**2⊿lnL**[b]<br>***P* value**[c]	**Positively Selected Sites (BEB Analysis)**[d]
**One ratio**	ω =0.0284	18	-3246.52		
**Branch-site Model**					
Branch q	site class 0 1 2a 2b<br>proportion 0.99379 0.00621 0.00000 0.00000<br>background w 0.02676 1.00000 0.02676 1.00000<br>foreground w 0.02676 1.00000 1.00000 1.00000	21	-3244.73	0<br>*P*=1	
Branch q<br>ω_a = 1	site class 0 1 2a 2b<br>proportion 0.99379 0.00621 0.00000 0.00000<br>background w 0.02676 1.00000 0.02676 1.00000<br>foreground w 0.02676 1.00000 1.00000 1.00000	20	-3244.73		

2. ATP8

**Model**	**Parameter Estimated**[a]	**p**	**lnL**	**2⊿lnL**[b]<br>***P* value**[c]	**Positively Selected Sites (BEB Analysis)**[d]
**One ratio**	ω =0.1176	18	-734.49		
**Branch-site Model**					
Branch q	site class 0 1 2a 2b<br>proportion 0.93873 0.06127 0.00000 0.00000<br>background w 0.09380 1.00000 0.09380 1.00000<br>foreground w 0.09380 1.00000 1.00000 1.00000	21	-731.15	0<br>*P*=1	
Branch q<br>ω_a = 1	site class 0 1 2a 2b<br>proportion 0.93873 0.06127 0.00000 0.00000<br>background w 0.09380 1.00000 0.09380 1.00000<br>foreground w 0.09380 1.00000 1.00000 1.00000	20	-731.15		

3. CoxI

**Model**	**Parameter Estimated**[a]	**p**	**lnL**	**2⊿lnL**[b]<br>***P* value**[c]	**Positively Selected Sites (BEB Analysis)**[d]
**One ratio**	ω =0.0184	18	-6073.85		
**Branch-site Model**					
Branch q	site class 0 1 2a 2b<br>proportion 0.98338 0.01662 0.00000 0.00000<br>background w 0.01350 1.00000 0.01350 1.00000<br>foreground w 0.01350 1.00000 1.00000 1.00000	21	-6055.21	0<br>*P*=1	
Branch q<br>ω_a = 1	site class 0 1 2a 2b<br>proportion 0.98338 0.01662 0.00000 0.00000<br>background w 0.01350 1.00000 0.01350 1.00000<br>foreground w 0.01350 1.00000 1.00000 1.00000	20	-6055.21		

4. CoxII

Model	Parameter Estimated[a]	p	lnL	2⊿lnL[b] *P* value[c]	Positively Selected Sites (BEB Analysis)[d]
**One ratio**	ω =0.0282	18	-2673.84		
**Branch-site Model**					
Branch q	site class 0 1 2a 2b<br>proportion 0.99035 0.00965 0.00000 0.00000<br>background w 0.02580 1.00000 0.02580 1.00000<br>foreground w 0.02580 1.00000 1.00000 1.00000	21	-2672.39	0<br>*P*=1	
Branch q<br>ω_a = 1	site class 0 1 2a 2b<br>proportion 0.99035 0.00965 0.00000 0.00000<br>background w 0.02580 1.00000 0.02580 1.00000<br>foreground w 0.02580 1.00000 1.00000 1.00000	20	-2672.39		

5. CoxIII

Model	Parameter Estimated[a]	p	lnL	2⊿lnL[b] *P* value[c]	Positively Selected Sites (BEB Analysis)[d]
**One ratio**	ω =0.0227	18	-3143.99		
**Branch-site Model**					
Branch q	site class 0 1 2a 2b<br>proportion 0.93541 0.01374 0.05011 0.00074<br>background w 0.01641 1.00000 0.01641 1.00000<br>foreground w 0.01641 1.00000 1.00000 1.00000	21	-3135.37	0<br>*P*=1	
Branch q<br>ω_a = 1	site class 0 1 2a 2b<br>proportion 0.93541 0.01374 0.05011 0.00074<br>background w 0.01641 1.00000 0.01641 1.00000<br>foreground w 0.01641 1.00000 1.00000 1.00000	20	-3135.37		

6. CytB

Model	Parameter Estimated[a]	p	lnL	2⊿lnL[b] *P* value[c]	Positively Selected Sites (BEB Analysis)[d]
**One ratio**	ω =0.0299	18	-5310.50		
**Branch-site Model**					
Branch q	site class 0 1 2a 2b<br>proportion 0.87866 0.03544 0.08257 0.00333<br>background w 0.01843 1.00000 0.01843 1.00000<br>foreground w 0.01843 1.00000 1.00000 1.00000	21	-5269.78	0<br>*P*=1	
Branch q<br>ω_a = 1	site class 0 1 2a 2b<br>proportion 0.87866 0.03544 0.08257 0.00333<br>background w 0.01843 1.00000 0.01843 1.00000<br>foreground w 0.01843 1.00000 1.00000 1.00000	20	-5269.78		

7. ND1

Model	Parameter Estimated[a]	p	lnL	2⊿lnL[b] *P* value[c]	Positively Selected Sites

					(BEB Analysis)[d]
**One ratio**	ω =0.0277	18	-4511.12		
**Branch-site Model**					
Branch q	site class 0 1 2a 2b<br>proportion 0.78924 0.00883 0.19969 0.00223<br>background w 0.02098 1.00000 0.02098 1.00000<br>foreground w 0.02098 1.00000 1.00000 1.00000	21	-4507.41	0<br>*P*=1	
Branch q<br>$\omega_a = 1$	site class 0 1 2a 2b<br>proportion 0.78924 0.00883 0.19969 0.00223<br>background w 0.02098 1.00000 0.02098 1.00000<br>foreground w 0.02098 1.00000 1.00000 1.00000	20	-4507.41		

8. ND2

Model	Parameter Estimated[a]	p	lnL	2⊿lnL[b]<br>*P* value[c]	Positively Selected Sites (BEB Analysis)[d]
**One ratio**	ω =0.0548	18	-5706.05		
**Branch-site Model**					
Branch q	site class 0 1 2a 2b<br>proportion 0.95307 0.04693 0.00000 0.00000<br>background w 0.04747 1.00000 0.04747 1.00000<br>foreground w 0.04747 1.00000 1.00000 1.00000	21	-5688.49	0<br>*P*=1	
Branch q<br>$\omega_a = 1$	site class 0 1 2a 2b<br>proportion 0.95307 0.04693 0.00000 0.00000<br>background w 0.04747 1.00000 0.04747 1.00000<br>foreground w 0.04747 1.00000 1.00000 1.00000	20	-5688.49		

9. ND3

Model	Parameter Estimated[a]	p	lnL	2⊿lnL[b]<br>*P* value[c]	Positively Selected Sites (BEB Analysis)[d]
**One ratio**	ω =0.0460	18	-1731.99		
**Branch-site Model**					
Branch q	site class 0 1 2a 2b<br>proportion 0.74186 0.09823 0.14121 0.01870<br>background w 0.01853 1.00000 0.01853 1.00000<br>foreground w 0.01853 1.00000 1.00000 1.00000	21	-1720.81	0<br>*P*=1	
Branch q<br>$\omega_a = 1$	site class 0 1 2a 2b<br>proportion 0.74186 0.09823 0.14121 0.01870<br>background w 0.01853 1.00000 0.01853 1.00000<br>foreground w 0.01853 1.00000 1.00000 1.00000	20	-1720.81		

10. ND4

Model	Parameter Estimated[a]	p	lnL	2⊿lnL[b]<br>*P* value[c]	Positively Selected Sites (BEB Analysis)[d]
**One ratio**	ω =0.0552	18	-6960.04		
**Branch-site Model**					

Branch q	site class 0 1 2a 2b<br>proportion 0.96652 0.02202 0.01120 0.00026<br>background w 0.04674 1.00000 0.04674 1.00000<br>foreground w 0.04674 1.00000 2.29577 2.29577	21	-6928.51	0.18<br>*P*=0.67	
Branch q<br>ω_a = 1	site class 0 1 2a 2b<br>proportion 0.95900 0.02213 0.01845 0.00043<br>background w 0.04652 1.00000 0.04652 1.00000<br>foreground w 0.04652 1.00000 1.00000 1.00000	20	-6928.60		

11. ND4L

**Model**	**Parameter Estimated**[a]	**p**	**lnL**	**2⊿lnL**[b]<br>***P* value**[c]	**Positively Selected Sites (BEB Analysis)**[d]
**One ratio**	ω =0.0728	18	-1307.80		
**Branch-site Model**					
Branch q	site class 0 1 2a 2b<br>proportion 0.84149 0.10050 0.05182 0.00619<br>background w 0.01694 1.00000 0.01694 1.00000<br>foreground w 0.01694 1.00000 1.24996 1.24996	21	-1274.24	0.04<br>*P*=0.84	
Branch q<br>ω_a = 1	site class 0 1 2a 2b<br>proportion 0.83290 0.09971 0.06019 0.00721<br>background w 0.01672 1.00000 0.01672 1.00000<br>foreground w 0.01672 1.00000 1.00000 1.00000	20	-1274.26		

12. ND5

**Model**	**Parameter Estimated**[a]	**p**	**lnL**	**2⊿lnL**[b]<br>***P* value**[c]	**Positively Selected Sites (BEB Analysis)**[d]
**One ratio**	ω =0.0523	18	-9165.47		
**Branch-site Model**					
Branch q	site class 0 1 2a 2b<br>proportion 0.94838 0.03089 0.02008 0.00065<br>background w 0.04317 1.00000 0.04317 1.00000<br>foreground w 0.04317 1.00000 1.00000 1.00000	21	-9131.05	0<br>*P*=1	
Branch q<br>ω_a = 1	site class 0 1 2a 2b<br>proportion 0.94838 0.03089 0.02008 0.00065<br>background w 0.04317 1.00000 0.04317 1.00000<br>foreground w 0.04317 1.00000 1.00000 1.00000	20	-9131.05		

13. ND6

**Model**	**Parameter Estimated**[a]	**p**	**lnL**	**2⊿lnL**[b]<br>***P* value**[c]	**Positively Selected Sites (BEB Analysis)**[d]
**One ratio**	ω =0.0479	18	-2713.11		
**Branch-site Model**					
Branch q	site class 0 1 2a 2b<br>proportion 0.95067 0.04933 0.00000 0.00000<br>background w 0.04112 1.00000 0.04112 1.00000	21	-2704.99	0<br>*P*=1	

	foreground w 0.04112 1.00000 1.00000 1.00000				
Branch q<br>ω_a = 1	site class 0 1 2a 2b<br>proportion 0.95067 0.04933 0.00000 0.00000<br>background w 0.04112 1.00000 0.04112 1.00000<br>foreground w 0.04112 1.00000 1.00000 1.00000	20	-2704.99		

[a] The proportion of sites estimated to have ω

[b] Twice the difference in log-likelihood values was calculated following a chi-squared ($\chi 2$)

[c] Posterior probability>0.99 of having ω>1

[d] The residues were identified by Bayes Empiriacal Bayes analysis
